# Recent Advances in the Oxone-Mediated Synthesis of Heterocyclic Compounds

**DOI:** 10.3390/molecules26247523

**Published:** 2021-12-12

**Authors:** Helen A. Goulart, Daniela R. Araujo, Filipe Penteado, Raquel G. Jacob, Gelson Perin, Eder J. Lenardão

**Affiliations:** Laboratório de Síntese Orgânica Limpa-LASOL-CCQFA, Universidade Federal de Pelotas-UFPel, P.O. Box 354, Pelotas 96010-900, RS, Brazil; helen-goulart@hotmail.com (H.A.G.); daniela.roodriguues@gmail.com (D.R.A.); penteado.filipe@gmail.com (F.P.); raquel.jacob@ufpel.edu.br (R.G.J.)

**Keywords:** Oxone, oxidant, heterocycles, green chemistry

## Abstract

Oxone is a commercially available oxidant, composed of a mixture of three inorganic species, being the potassium peroxymonosulfate (KHSO_5_) the reactive one. Over the past few decades, this cheap and environmentally friendly oxidant has become a powerful tool in organic synthesis, being extensively employed to mediate the construction of a plethora of important compounds. This review summarizes the recent advances in the Oxone-mediated synthesis of N-, O- and chalcogen-containing heterocyclic compounds, through a wide diversity of reactions, starting from several kinds of substrate, highlighting the main synthetic differences, advantages, the scope and limitations.

## 1. Introduction

The 12 Principles of Green Chemistry, introduced by Anastas and Warner at the end of the 1990s, set guidelines for the development of sustainable and environmentally friendly chemical processes, aiming to reduce and/or eliminate the chemical pollution by improving design, instead of residue treatment. Among them, Principle #12 (Inherently Safer Chemistry for Accident Prevention) is very important, once it encourages the use of safe substrates and reaction conditions, as well as seeking to eliminate the generation of hazardous substances, reducing the risk of accidents involving explosions, leaks and fires [1,2,3].

To satisfy this premise, the development of selective protocols, employing green oxidant species and circumventing the use of hazardous oxidants (e.g.,: KMnO_4_, K_2_Cr_2_O_7_, CrO_3_, OsO_4_, *^t^*BuOCl, *m*-CPBA, NBS), are welcome. Besides being operationally dangerous, some oxidants also produce toxic organic- and heavy metal-based byproducts, enhancing the environmental and health impact [4]. On the other hand, molecular oxygen (O_2_) and hydrogen peroxide (H_2_O_2_) are, undoubtedly, important green oxidant reagents, due to the formation of non-toxic and environmentally benign byproducts, besides presenting an excellent atom efficiency. However, transportation and handling of these species are hugely faced problems [5].

In this context, Oxone (a white solid, molar mass = 307 g mol^−1^) plays an important role, being a cheap and commercially available compound, composed of three inorganic salts (KHSO_5_/0.5KHSO_4_/0.5K_2_SO_4_), among which potassium peroxymonosulfate (KHSO_5_) is the reactive species. Oxone has been widely used as a green oxidant in organic transformations, generating the nontoxic KHSO_4_ as the oxidant-derived byproduct. Additionally, it is a very safe species, presenting an impressive bench-stability, which makes its transportation and storage not dangerous [6].

Heterocycles are considered one of the most important class of compounds, widely present in nature. They are also valuable in the manufacturing industry (including pharmaceuticals, agrochemicals and materials), in which they play a pivotal role [7,8]. This importance is evident regarding that around 60% of the small-molecule-approved drugs, registered in the U.S. FDA database, are composed of at least one heterocycle unit [9,10]. For this reason, the search for simple and efficient methods to access these compounds is constant and intense, with hundreds of new strategies emerging each year.

**Scheme 1:**
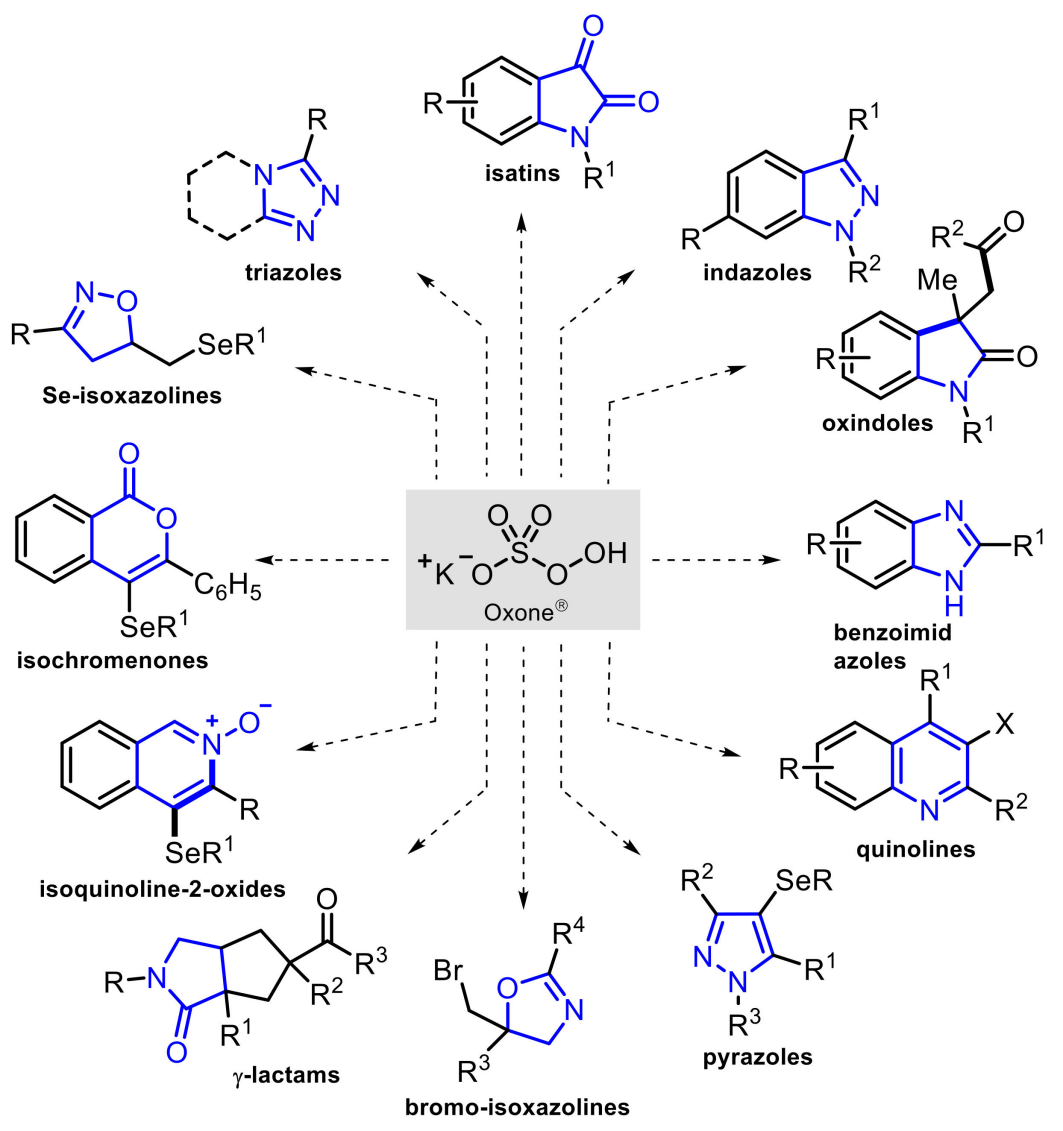
Oxone-mediated synthesis of heterocyclic compounds.

Regarding the synthetic versatility of Oxone as a green oxidant, and the massive search for the development of innovative and efficient methods to prepare heterocyclic compounds, several studies have been published describing the Oxone-mediated synthesis of heterocycles through a wide diversity of strategies (Scheme 1). Thus, this review aims to emerge a comprehensive discussion about the recent advances in this field, covering the relevant works published between 2013 and 2021. For a better understanding, it is divided in four major sections, which involve the synthesis of nitrogen-containing heterocycles, oxygen-containing heterocycles, chalcogen-containing heterocycles and miscellaneous cyclizations.

## 2. Nitrogen-Containing Heterocycles

*N*-based heterocycles are the most abundant among this important class of compounds [11]. This importance is closely related to some impressive properties, including a wide range of biological activities [12,13], being present in several worldwide marketed drugs [9] and agrochemicals, and are important in the development of functional materials [14]. As a consequence, there is an increasing search, by the scientific community, for the development of efficient methods to access these compounds. Among the recent advances, several Oxone-promoted protocols have emerged as efficient and greener alternatives to prepare *N*-based heterocycles.

**Scheme 2:**
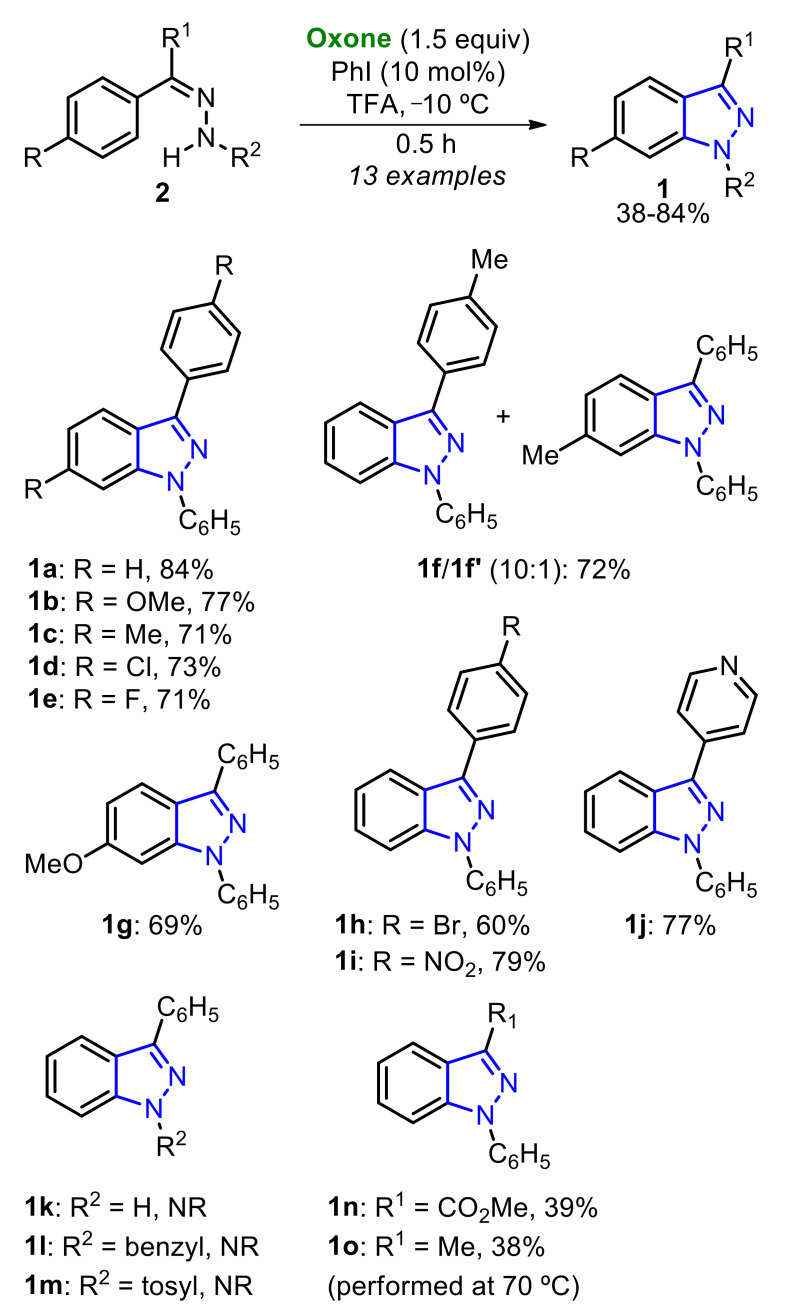
Oxone/PhI-promoted annulation of *N*-substituted hydrazones **2**.

In 2014, Tanimori and co-workers [15] described a transition metal (TM)-free protocol for the synthesis of *N*-arylsubstituted 1*H*-indazole and derivatives **1** (Scheme 2). The optimized reaction condition was reached after stirring a mixture of hydrazones **2**, iodobenzene (10 mol%), Oxone (1.5 equiv) and trifluoroacetic acid (TFA), at −10 °C for 0.5 h. The cyclization reaction scope was extended to several substituted hydrazones **2**, affording the respective 1*H*-indazoles **1** at up to an 84% yield. Electron-rich disubstituted hydrazones **2b** and **2c** (R = Me and OMe), gave the corresponding substituted 1*H*-indazoles **1b** and **1c** at a 77% and 71% yield, respectively. Halogen-substituted hydrazones **2d** and **2e** (R = Cl and F) also reacted smoothly to produce the desired products **1d** and **1e** in 73% and 71% yield, respectively. It is worth to mention that *p*-methyl monosubstituted hydrazone **2f** provided a 10:1 mixture of the regioisomeric products **1f** and **1f’** at a 72% yield. This problem was not observed when the *p*-methoxy-substituted substrate **2g** was reacted, yielding the respective product **1g** (69% yield) with high regioselectivity, through the annulative process undergoing in the electron-rich ring. Satisfactorily, *p*-NO_2_ and pyridinyl hydrazones **2i** and **2j** reacted successfully to afford the products **1i** and **1j** at a 79% and 77% yield, respectively. Nevertheless, limitations were also faced when *N*-H, *N*-benzyl and *N*-tosyl hydrazones **2k m** were employed as substrate. In these cases, the desired 1*H*-indazoles **1k**–**m** could not be obtained, probably due to the low stability of the nitrenium ion, the key intermediate of the reaction. Hydrazones containing CO_2_Me- and Me groups **2n** and **2o** were suitable substrates for this reaction; however, the respective indazoles **1n** and **1o** were obtained in poor yields (Scheme 2). 

**Scheme 3:**
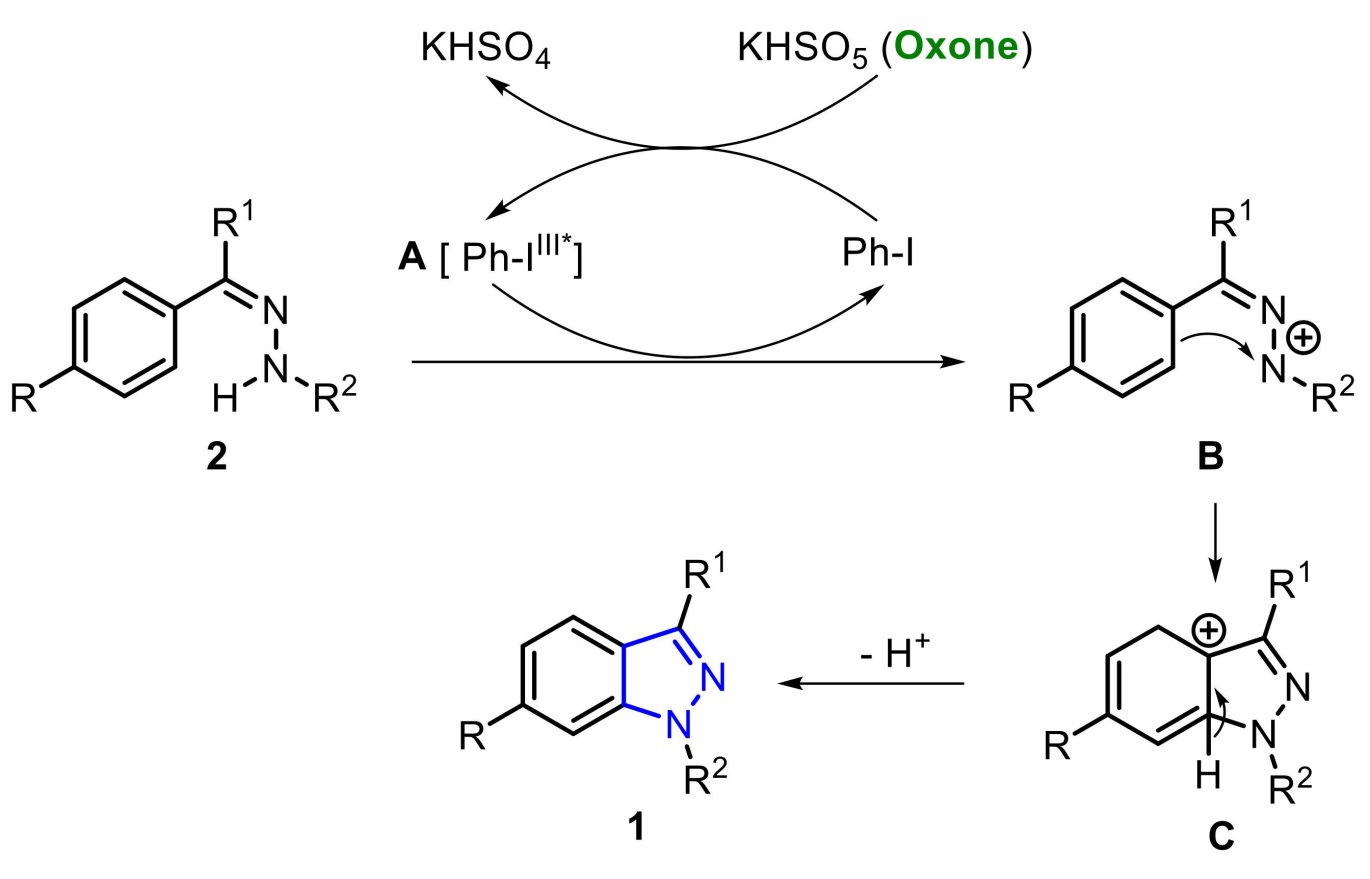
Plausible reaction mechanism for the Oxone/PhI-promoted annulation process.

Based on control experiments, a plausible reaction mechanism was proposed (Scheme 3). Initially, the oxidation of iodobenzene by Oxone affords the hypervalent iodine(III) species **A**, which reacts with **2** to form the nitrenium ion intermediate **B**. Then, an intramolecular electrophilic substitution provides the carbocation **C**, which is finally converted to the aromatic product **1**. 

**Scheme 4:**
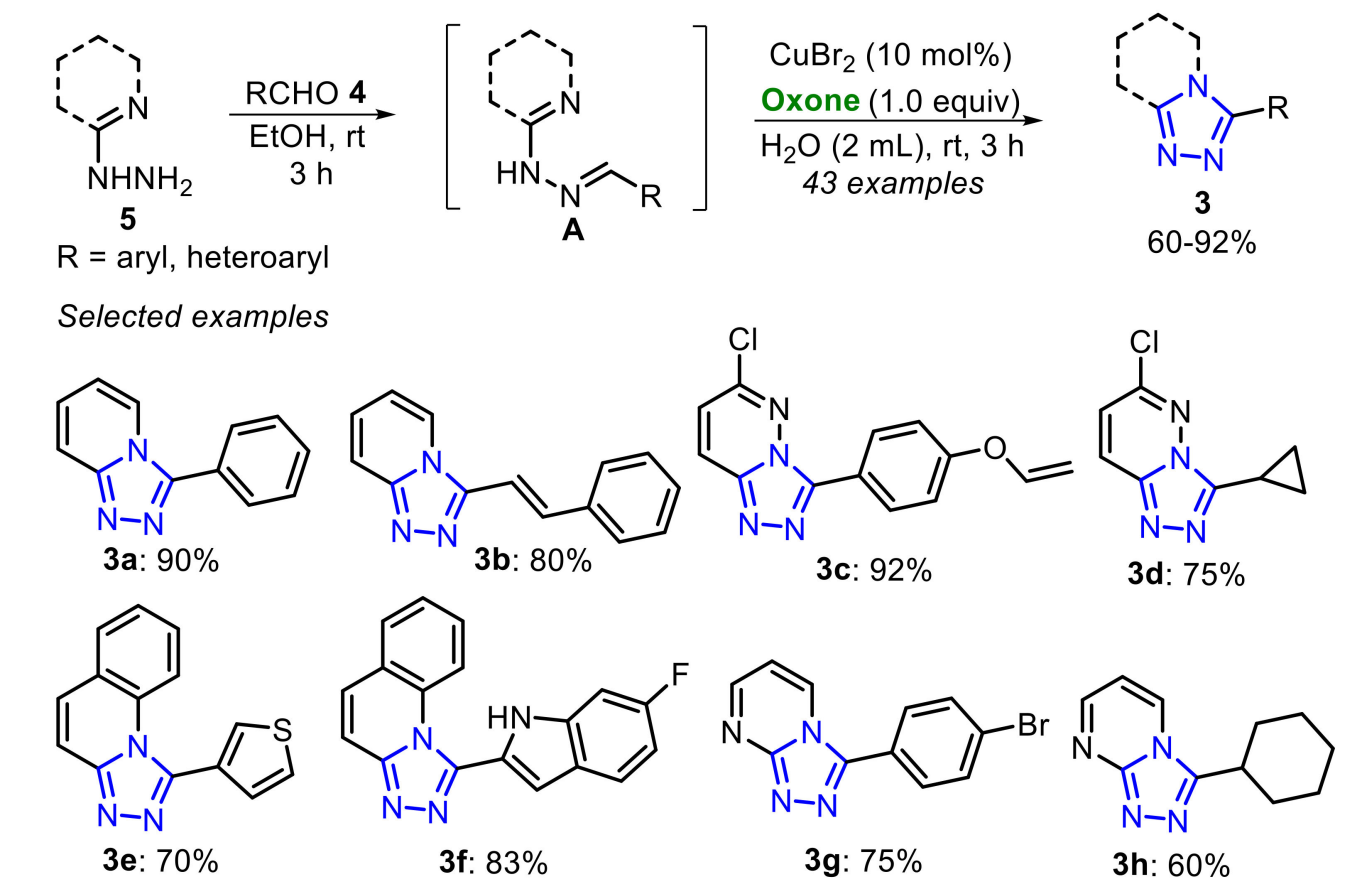
Oxone/CuBr-mediated synthesis of triazole-fused heterocycles **3**.

In 2015, Nagarajan and co-workers reported the synthesis of triazole-fused heterocycles **3** through a one-pot strategy, starting from aldehydes **4** and heteroarylhydrazines **5**. The reaction involves the formation in situ of the hydrazone **A**, which in the sequence is submitted to an Oxone/CuBr-mediated intramolecular oxidative cyclization (Scheme 4) [16]. Through this simple and selective system, several substituted aldehydes bearing a wide range of functional groups were employed as substrate, affording a diversity of triazole-fused pyridines, pyridazines, pyrimidine and quinolines **3**, in good yields and short reaction times. 

**Scheme 5:**
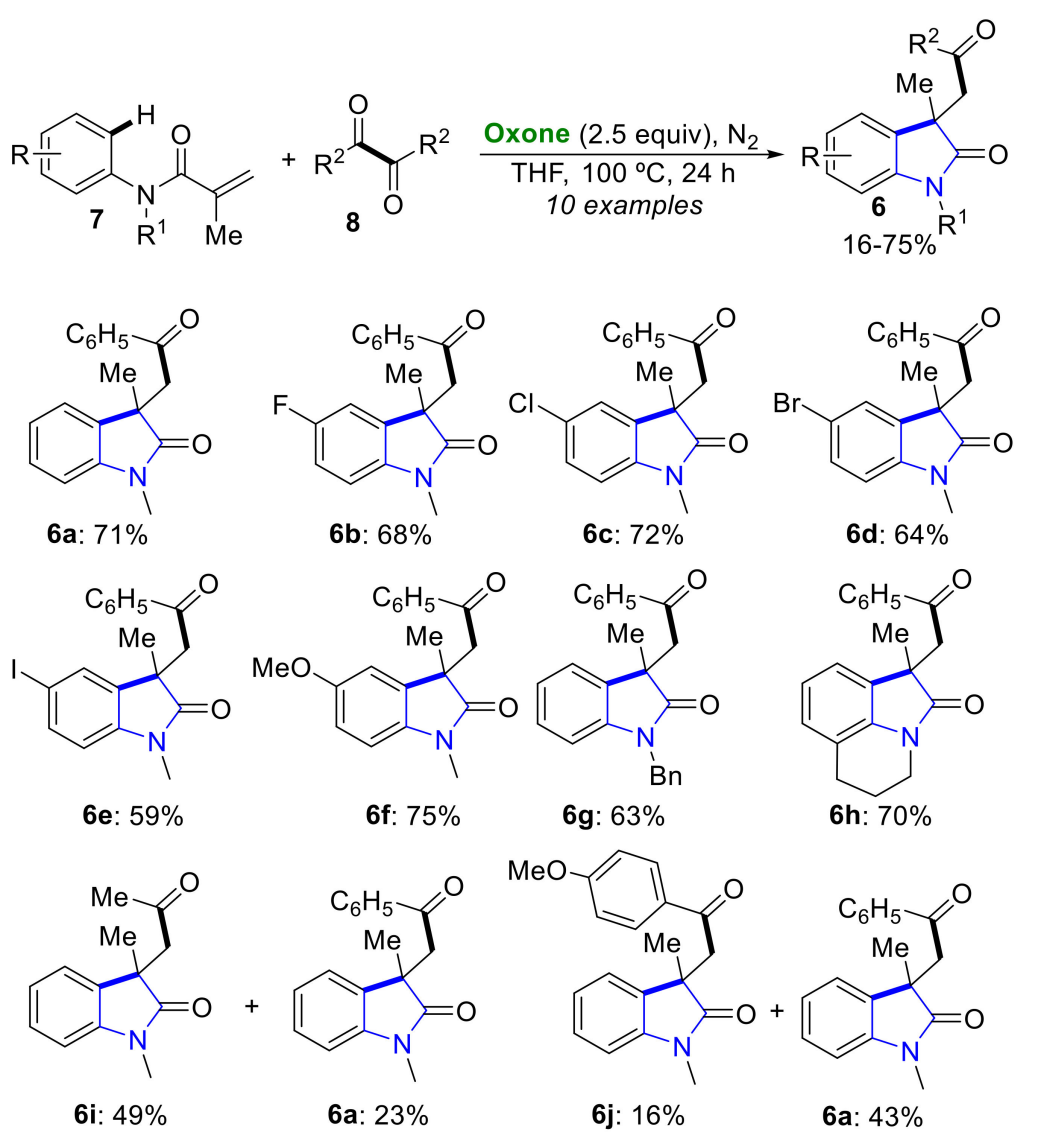
Oxone-mediated synthesis of oxindoles **6**.

Additionally, in 2015, Guo and co-workers described the synthesis of oxindoles **6** through an Oxone-mediated reaction between *N*-arylacrylamides **7** and *α*-diketones **8**, through a C*sp*^2^−C*sp*^2^ cleavage and the formation of a new C*sp*^2^−C*sp*^3^ bond (Scheme 5) [17]. The optimized reaction condition was set by using *N*-arylacrylamina **7**, *α*-diketone **8**, and Oxone (2.5 equiv) in THF (3 mL) as solvent. The resulting mixture was stirred at 100 °C under N_2_ atmosphere for 24 h. Under this condition, a total of ten 3-(2-oxoethyl)indolin-2-ones **6** was obtained in poor to good yields, employing several electron-rich and electron-deficient substrates **7** and **8**. It is worth to mention that asymmetric *α*-diketones, including 1-phenylpropane-1,2-dione **8a** and 1-(4-methoxyphenyl)-2-phenylethane-1,2-dione **8b**, were also suitable substrates, resulting a mixture of products **6a** + **6i** (23% and 49% yield) and **6a** + **6j** (43% and 16% yield), respectively (Scheme 5).

**Scheme 6:**
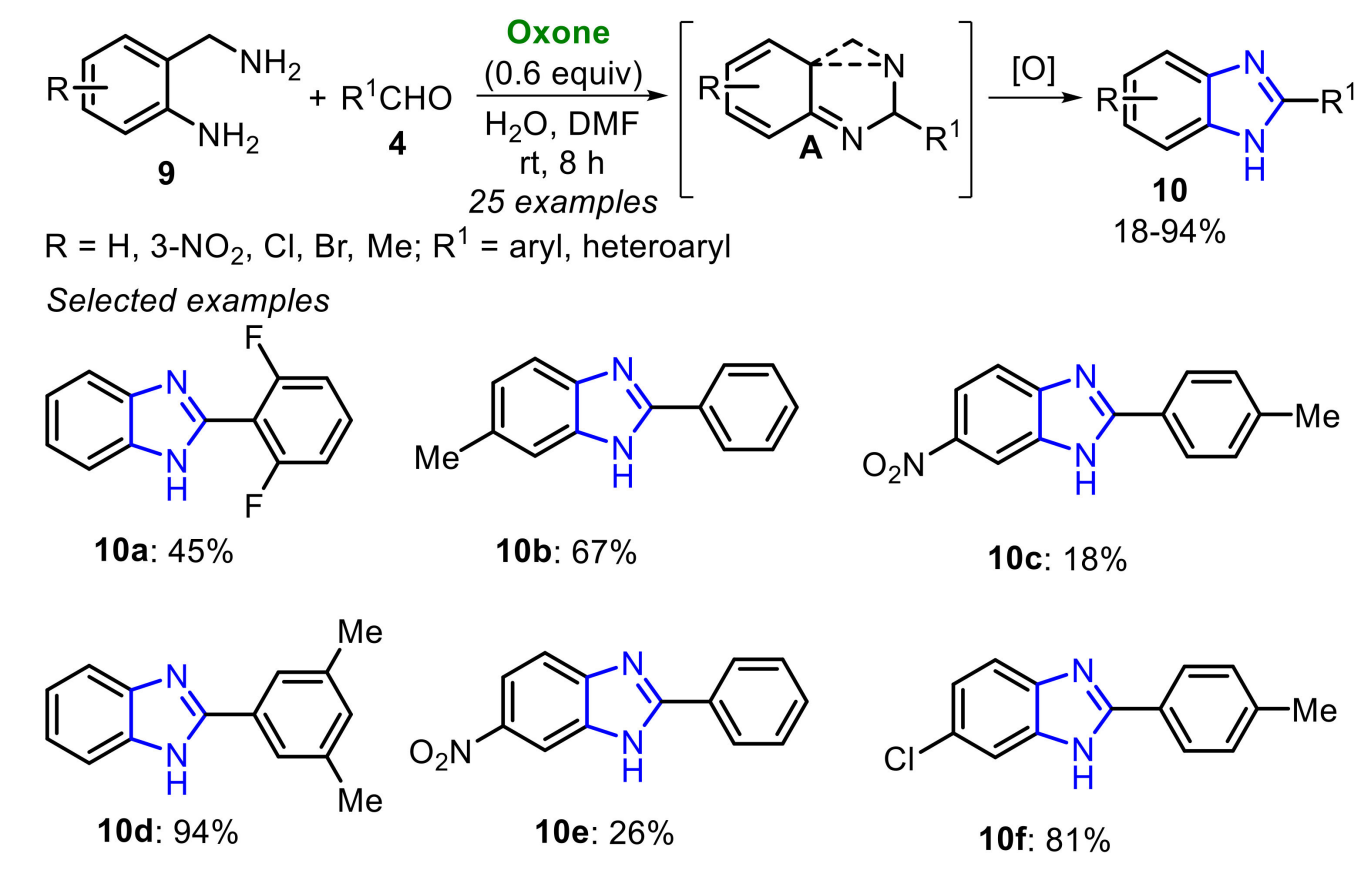
Oxone-mediated synthesis of 2-substituted benzimidazoles **10**.

In 2016, Sen and co-workers employed Oxone as an oxidant in the tandem annulation between 2-aminobenzylamines **9** and aldehydes **4**, in order to access 2-substituted benzimidazoles **10** (Scheme 6) [18]. The reaction is conducted at room temperature for 8 h, in the presence of Oxone (0.6 equiv), H_2_O (2 mL) and DMF (10 mL), and was suitable to several aliphatic, aromatic and heteroaromatic aldehydes **4**, in the presence of substituted 2-aminobenzylamines **9**, giving the products **10** in poor to excellent yields. Limitations were faced when electron-deficient 2-aminobenzylamines (R = 3-NO_2_) were employed as substrate, affording the products **10c** and **10e** in 18% and 26%, respectively. The process involves the initial condensation between 2-aminobenzylamine **9** and aldehydes **4**, providing the tetrahydroquinazoline intermediate **A**, which undergoes a radical ring distortion to afford the desired products **10**, under oxidative conditions (Scheme 6).

**Scheme 7:**
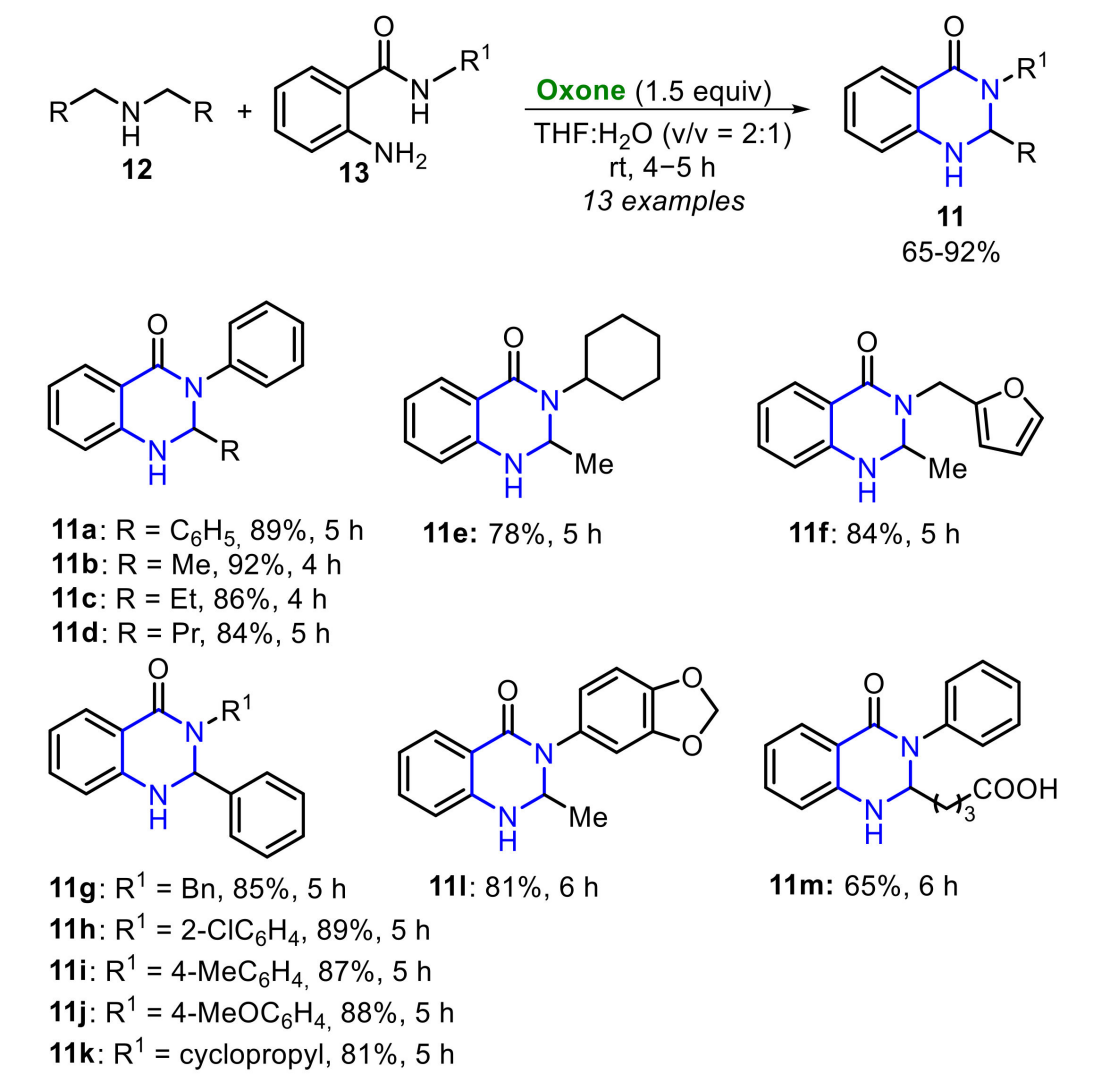
Oxone-promoted synthesis of 2,3-dihydroquinazoline-4(1*H*)-ones **11a**–**m**.

In 2018, Madabhushi and co-workers described an efficient Oxone-mediated protocol for the preparation of 2,3-dihydroquinazolin-4(1*H*)-ones **11** and 1*H*-benzimidazoles **10** (Scheme 7) [19]. The reaction involves stirring a mixture of *sec*-amines **12**, via imine-*N*-oxides, and substituted 2-amino-*N*-benzamides **13** in the presence of Oxone (3.54 mmol), in a mixture of THF/water (2:1, 10 mL) as solvent, affording the respective 2,3-dihydroquinazoline-4(1*H*)-ones **11a** m in moderate to excellent yields. The reaction scope was satisfactorily expanded, and several alkyl and aryl *sec*-amines **12** were employed, as well as *N*-substituted benzamides **13**, bearing aliphatic, aromatic and heteroaromatic groups.

**Scheme 8:**
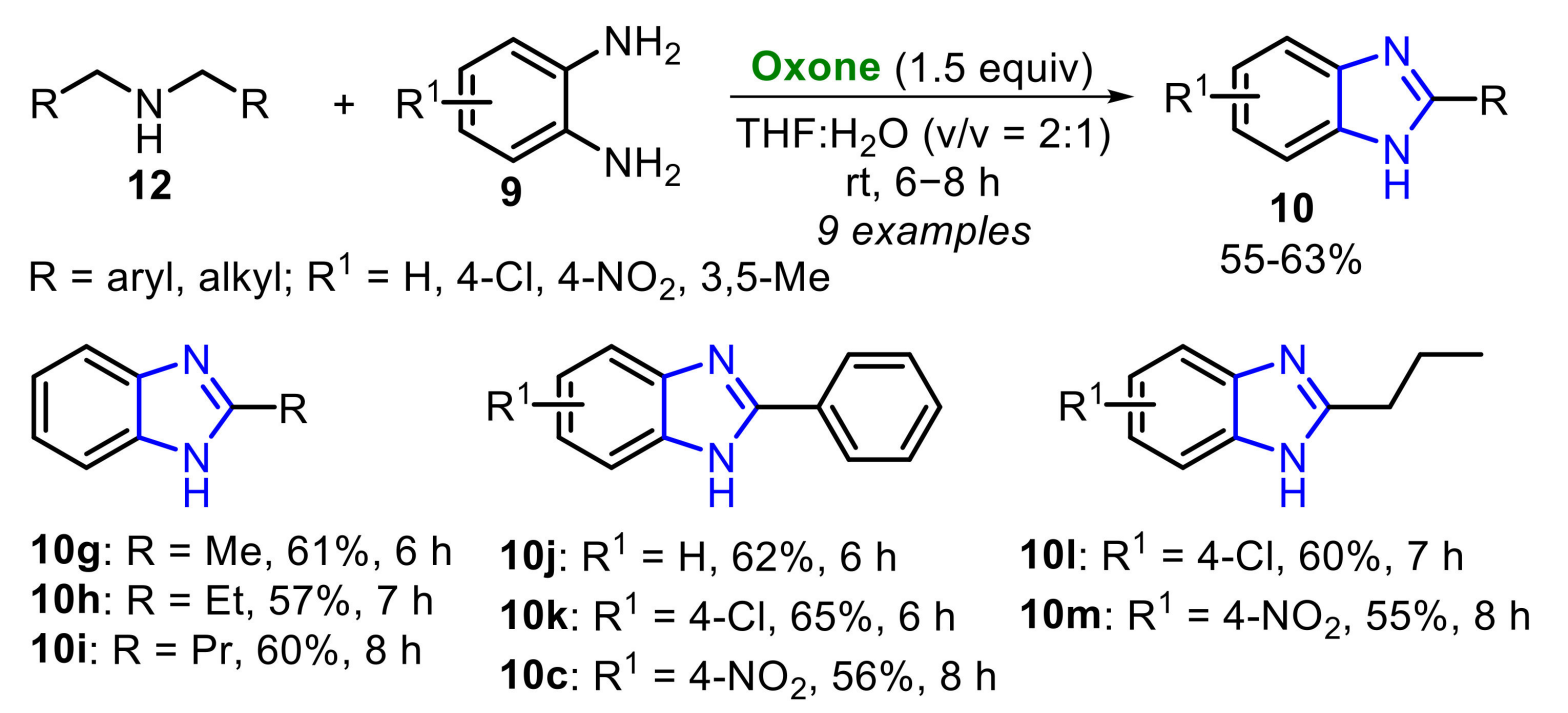
Oxone-promoted synthesis of 1*H*-benzimidazoles **10**.

Additionally, under the optimized reaction condition, *sec*-amines **12** reacted with 1,2-diaminobenzenes **9**, affording 1*H*-benzimidazoles **10** in poor to moderate yields (Scheme 8). The reaction scope was also investigated, presenting an acceptable substrate tolerance, including the use of substrates bearing the strong electron-withdrawing nitro group (R^1^ = 4-NO_2_).

**Scheme 9:**
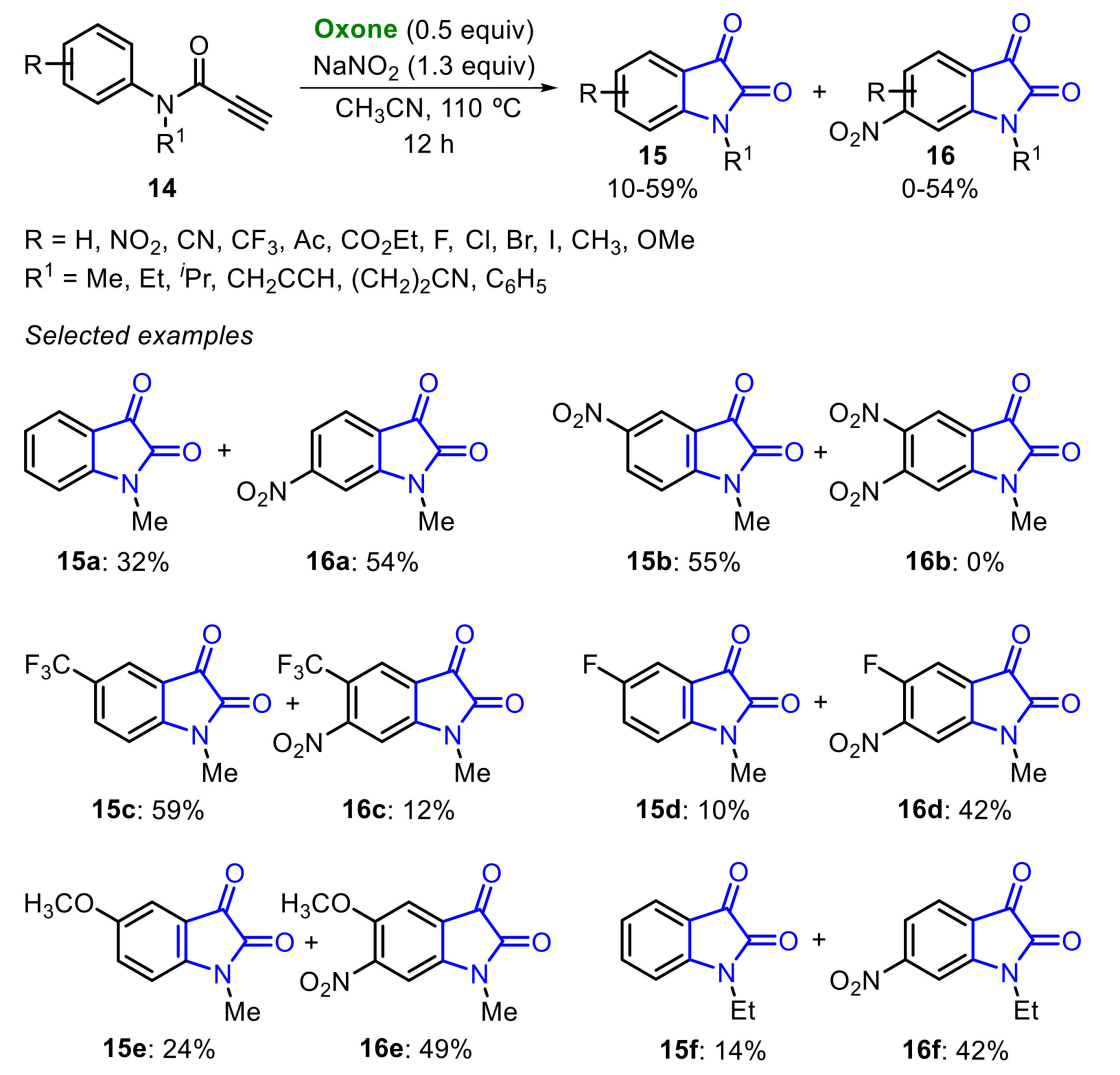
Oxone/NaNO_2_-promoted oxidative radical cyclization of the propylamide **14**.

In 2018, Tang, Zheng and co-workers [20] described an Oxone/NaNO_2_-mediated oxidative radical cyclization of *N*-methyl-*N*-arylpropiolamide **14** to the respective isatins **15** and **16**, through the oxidative cleavage of the C≡C bond, affording the desired products in moderate to good yields (Scheme 9). Despite the low reaction selectivity, the present protocol is important, once NO_2_-containing isatin derivatives **16** are satisfactorily afforded, which is not an easy task, from the synthetic point of view.

**Scheme 10:**
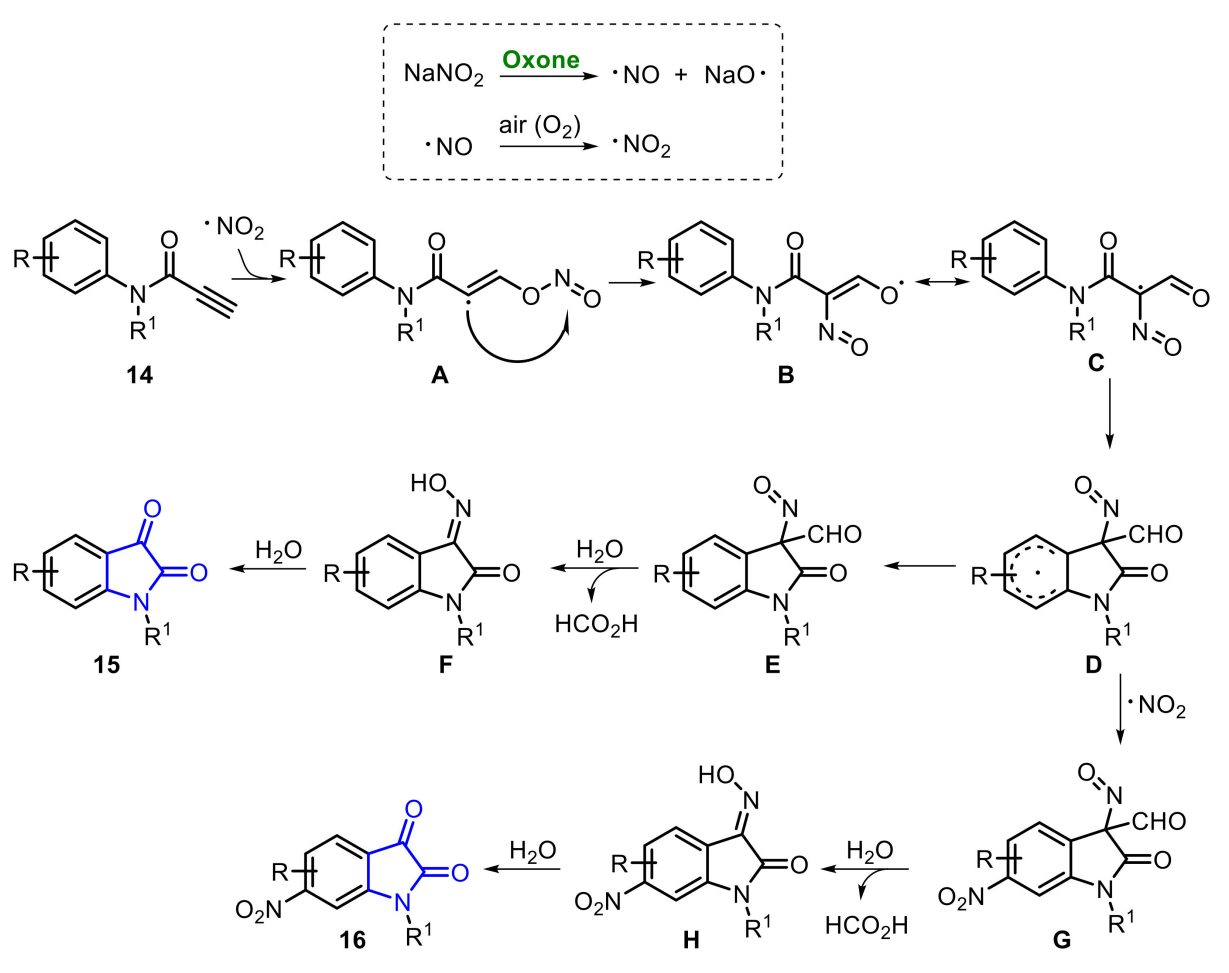
Reaction mechanism for the Oxone/NaNO_2_-promoted oxidative annulation.

The proposed reaction mechanism involves an initial Oxone-promoted oxidation of NaNO_2_ to the •NO radical species, under thermal condition. Thus, •NO radical is oxidized in the presence of O_2_ to •NO_2_, which is trapped by the C≡C triple bond, to afford the intermediate **A**. Following, a radical isomerization produces the intermediate **B**, which is quickly converted by electron delocalization to the tertiary radical species **C**, that undergoes an intramolecular radical annulation to produce the cyclized intermediate **D**. A hydrogen radical elimination converts the aryl radical **D** to the intermediate **E**, which after a decarbonylation, affords the intermediate **F**. The intermediate **D** can also be trapped by 1 equivalent of •NO_2_ radical to produce the intermediate **G**, which is subsequently decarbonylated to generate the intermediate **H**. Finally, the intermediates **F** and **H** are hydrolyzed and converted to the respective products **15** and **16** (Scheme 10).

**Scheme 11:**
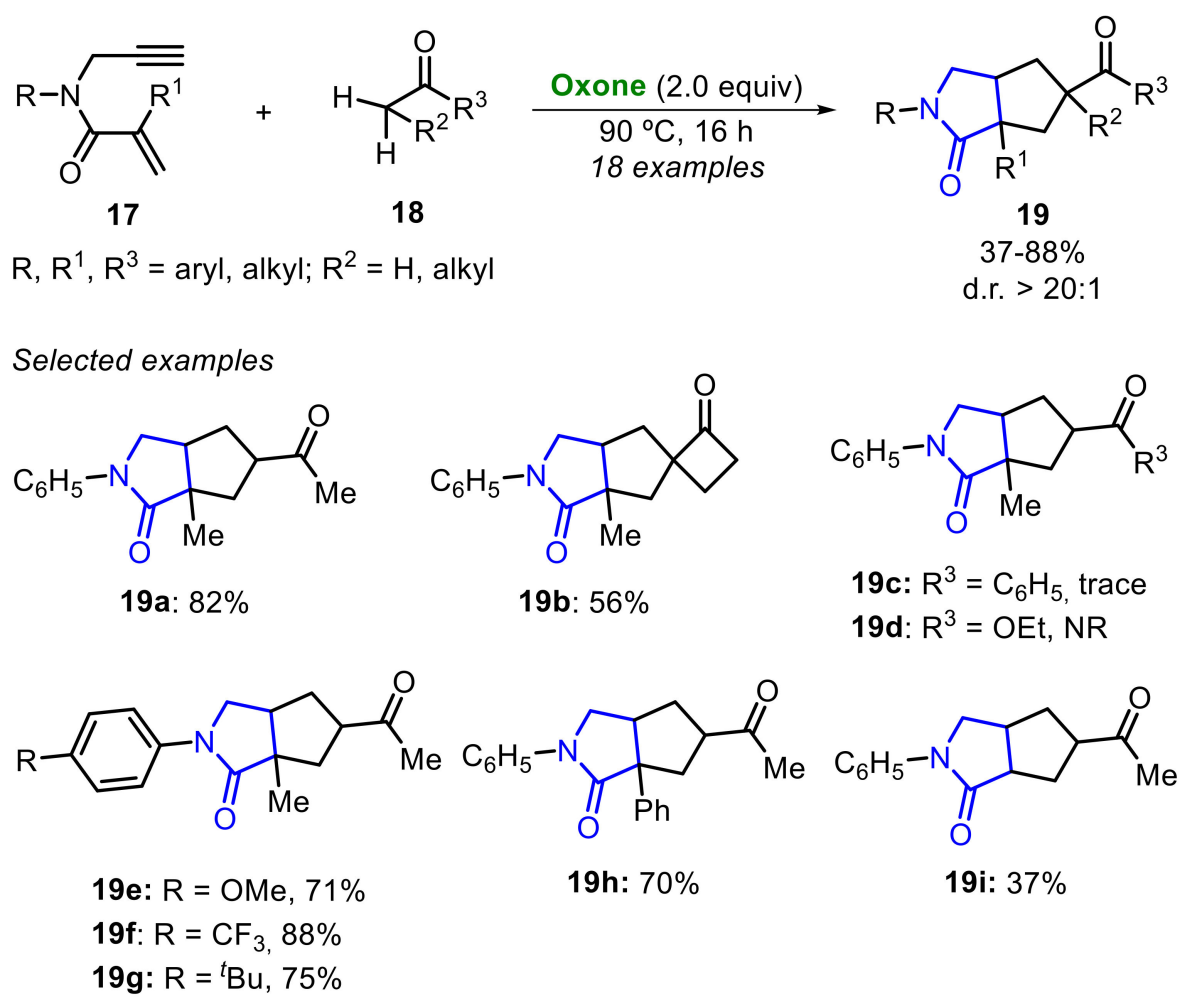
Oxone-mediated radical bicyclization of 1,6-enynes **17** and ketones **18**.

In 2019, Wei and co-workers reported an Oxone-mediated base-free radical bicyclization of 1,6-enynes **17**, in the presence of ketones **18**, affording several functionalized polycyclic *γ*-lactams **19** (Scheme 11) [21]. Under the optimal conditions, aliphatic ketones, such as acetone **18a** and cyclobutanone **18b**, reacted satisfactorily with 1,6-enyne **17a**, yielding the respective products **19a** and **19b** in 82% and 56% yield, respectively. On the other hand, acetophenone **18c** and ethyl acetate **18d** were not suitable substrates, which may be presumably attributed to the inertness of the *α*-C*sp*^3^−H bond to generate the corresponding *α*-carbonyl radical species. Additionally, a range of 1,6-enynes were well tolerated as the substrate, giving the respective polycyclic *γ*-lactams **19e**–**i** at up to an 88% yield (Scheme 11).

**Scheme 12:**
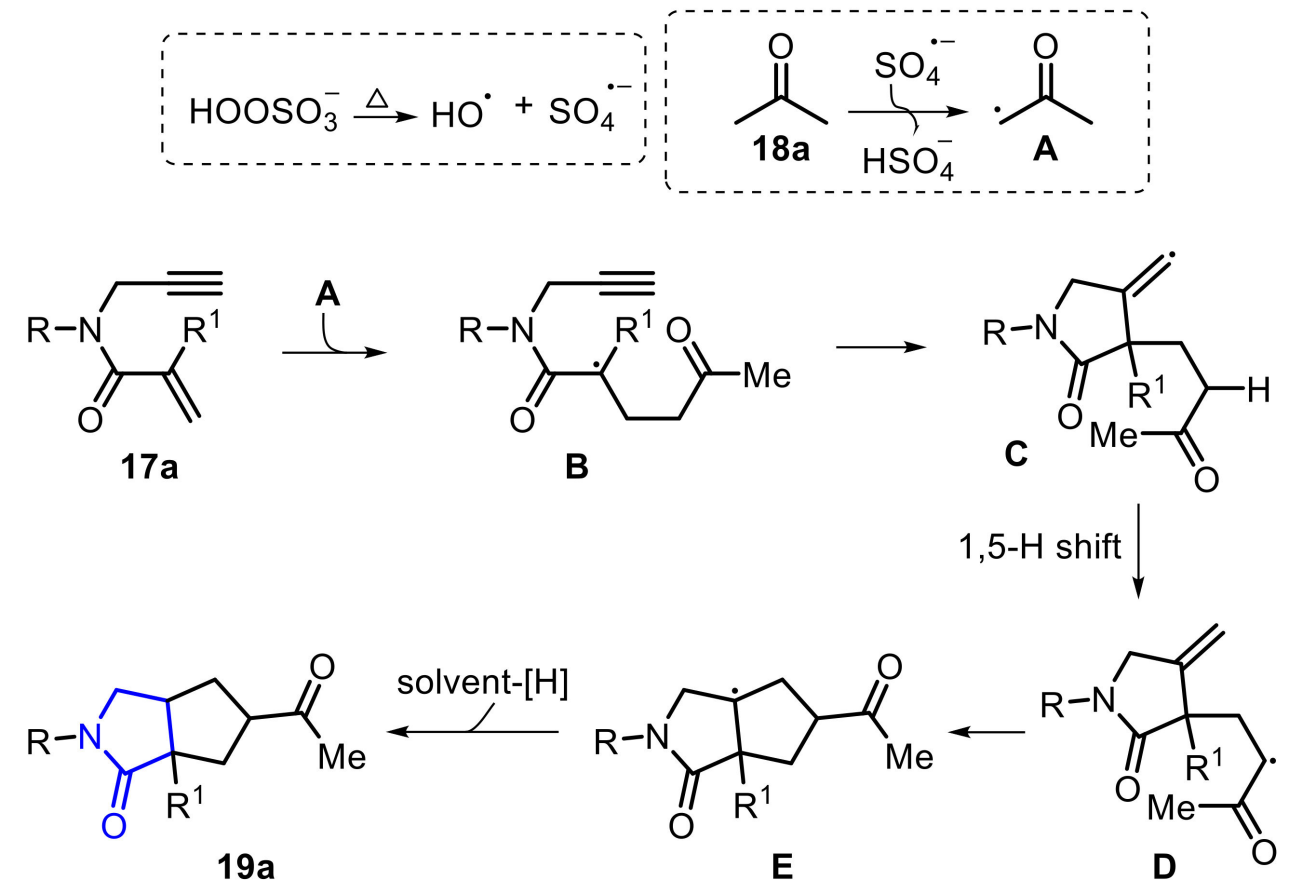
Reaction mechanism for the Oxone-promoted bicyclization of 1,6-enynes **17**.

The reaction mechanism to prepare the *γ*-lactams **19** is depicted in Scheme 12. Initially, the *α*-carbonyl radical **A**, which is formed after heating the ketone **18a** in the presence of Oxone, is added to the terminal C≡C double bond (of 1,6-enyne **17**), giving the alkyl radical intermediate **B**. After an intramolecular cyclization, the tertiary radical **B** is converted to the vinyl radical intermediate **C**, which undergoes an 1,5-H shift, to be converted to the intermediate **D**. Finally, the second cyclization process takes place, generating the intermediate **E**, that through a homolytic hydrogen abstraction is converted to the desired product **19**.

**Scheme 13:**
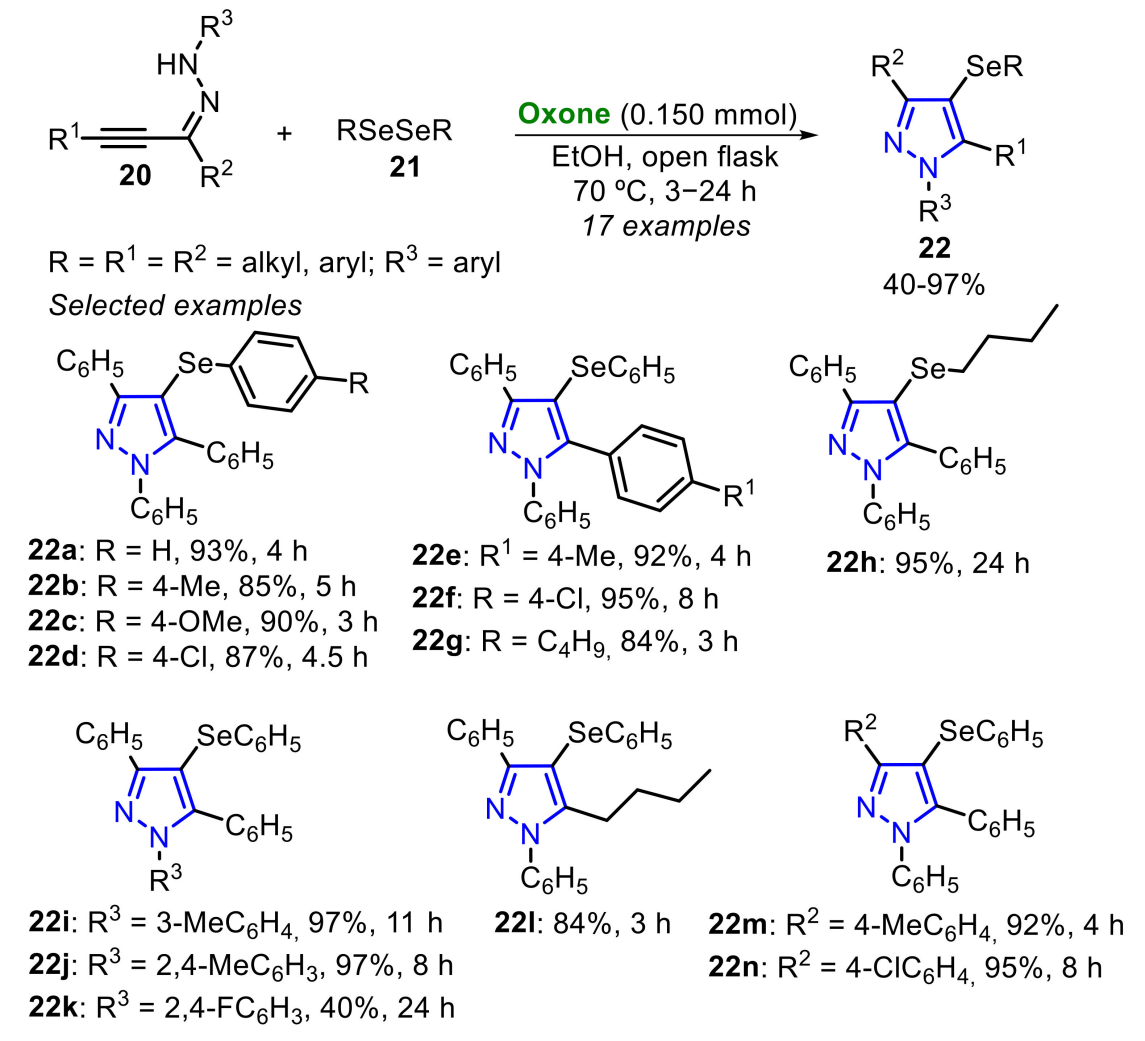
Oxone/RSeSeR-promoted electrophilic cyclization of *α,β*-alkynyl hydrazones **20**.

Additionally, in 2019, some of us reported the electrophilic cyclization of *β*-alkynyl hydrazones **20**, promoted by the system Oxone/RSeSeR **21**, to obtain 4-organoselanyl-1*H*-pyrazoles **22** (Scheme 13) [22]. The reaction scope was studied employing several substituted diorganyl diselenide **21** and hydrazones **20**, affording the desired products **22** in poor to excellent yields. A limitation was faced when the *α,β*-alkynyl hydrazone **20k**, bearing an electron-deficient ring (R^3^ = 2,4-F_2_C_6_H_3_), was employed as substrate, providing the product **22k** at only a 40% yield after 24 h. Better results were obtained when electron-rich *α,β-*alkynyl hydrazones **20** (R^3^ = 3-MeC_6_H_4_ and 2,4-Me_2_C_6_H_3_) were reacted, yielding the products **22i** and **22j** at 97%, after 11 and 8 h of reaction, respectively (Scheme 13).

**Scheme 14:**
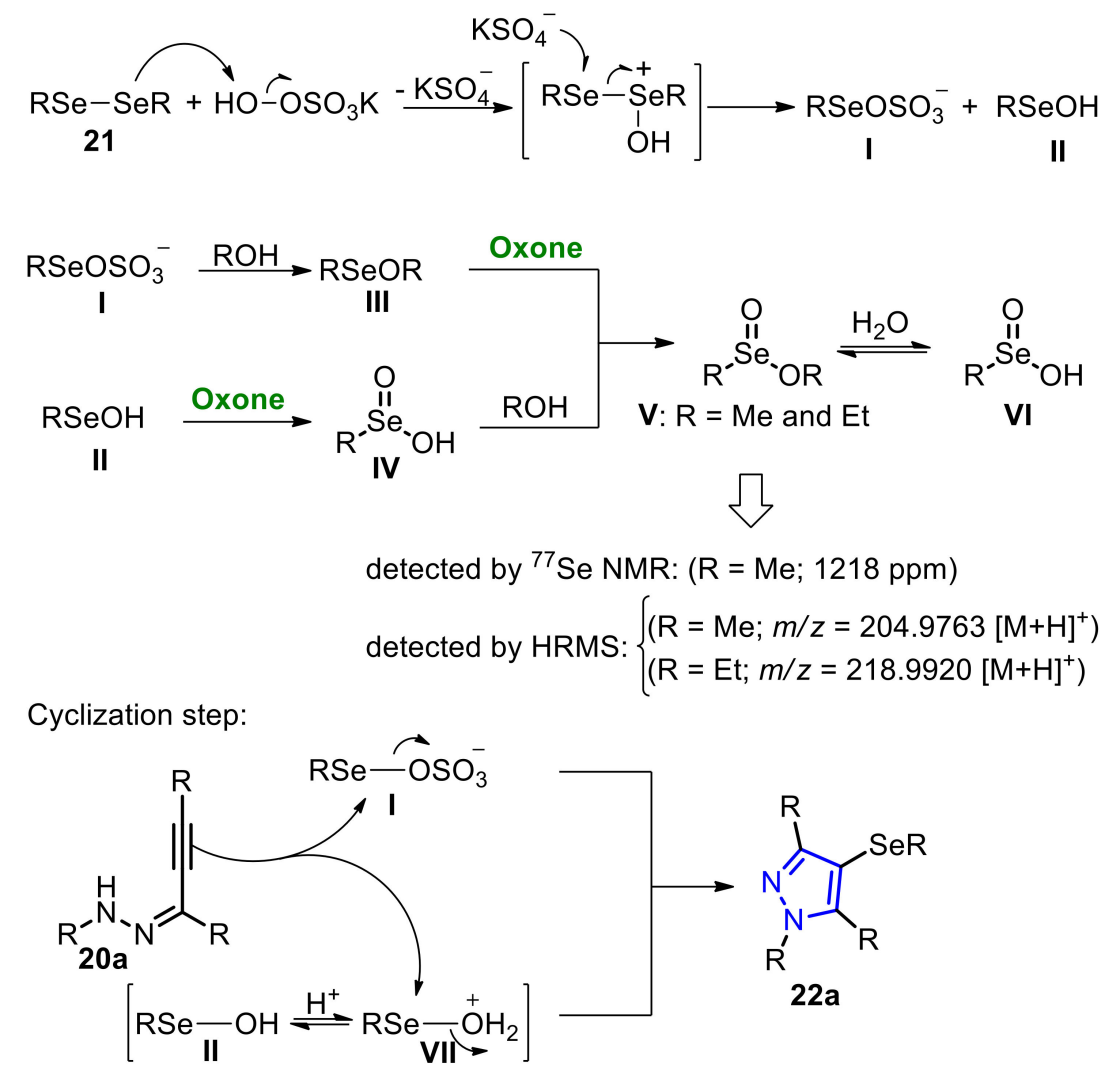
Reaction mechanism for the Oxone/RSeSeR-promoted electrophilic cyclization of *α,β*-alkynyl hydrazones **20**.

During control experiments, the in situ generation of Se-based electrophilic species was identified by ^77^Se NMR and HRMS, which were the key intermediates to disclose the annulation pathway. The proposed mechanism starts with a SET from diphenyl diselenide **21** to the HSO_5_^-^ species, followed by the cleavage of the Se–Se bond, providing the Se-based electrophiles PhSeOSO_3_^-^ **I** and PhSeOH **II**, which promote the electrophilic annulation of the *α*,*β*-alkynyl hydrazone **20**, to afford the desired products **22**. It is worth to mention that species **I** and **II**, in the presence of MeOH and Oxone, can be converted to the methyl benzeneseleninate **V** and benzeneseleninic acid **VI**, respectively (Scheme 14).

**Scheme 15:**
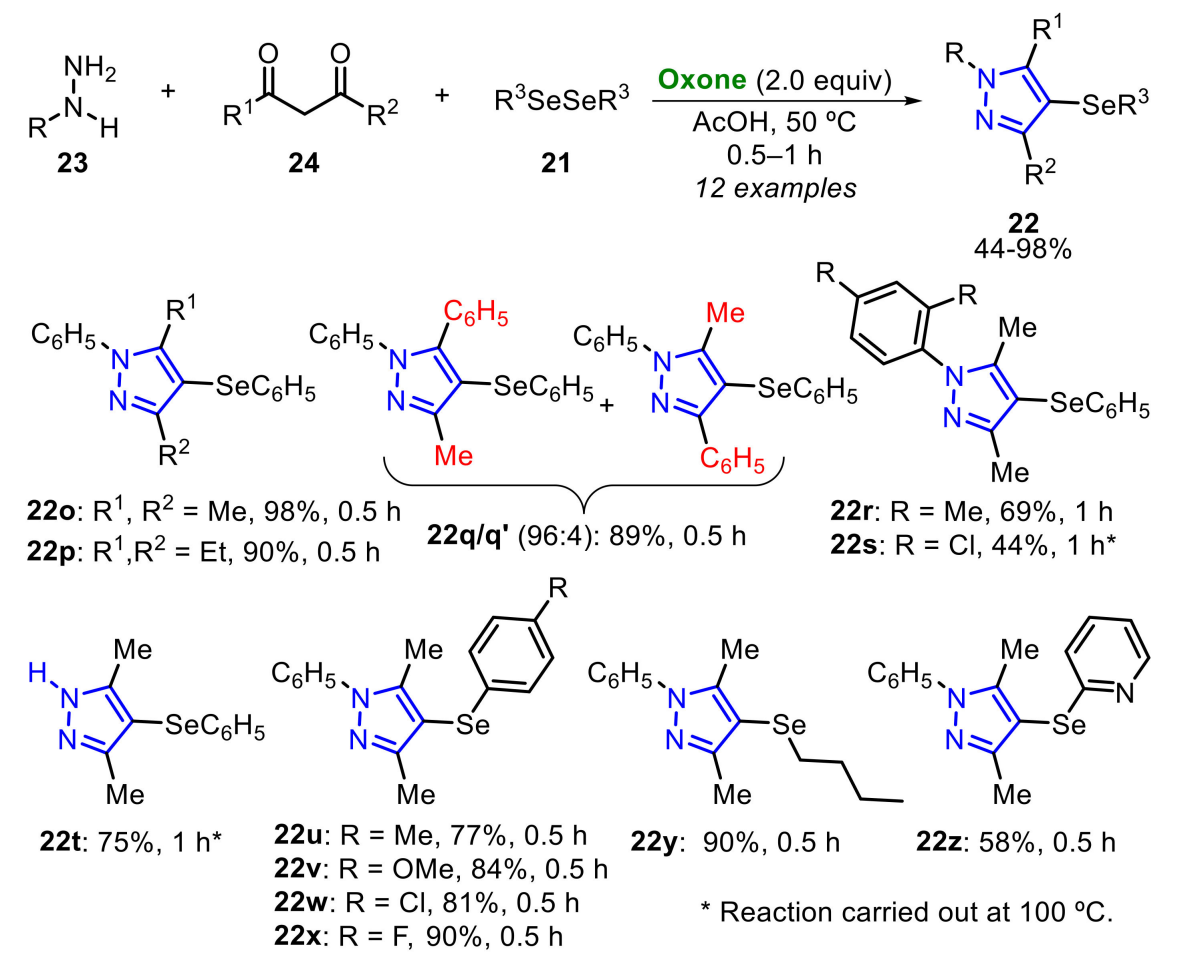
Oxone-mediated multicomponent synthesis of substituted 4-organylselanylpyrazoles **22**.

In the same year, Jacob and co-workers also reported the synthesis of 4-organoselanyl-1*H*-pyrazoles **22** through a multicomponent reaction between hydrazines **23**, 1,3-diketones **24** and diorganyl diselenides **21**, in the presence of Oxone (Scheme 15) [23]. In general, the protocol presented a good substrate tolerance, allowing the synthesis of twelve substituted 4-organylselanylpyrazoles **22** in moderate to excellent yields, after short reaction times. When the unsymmetrical 1-phenyl-1,3-butanedione **24c** was employed as substrate, two regioisomers **22q** and **22q’** in a 96:4 ratio were obtained at 89% yield. Authors attributed the high selectivity to the steric hinderance of ketone **24c** and also to the conjugative effect of the aromatic ring, that can contribute to stabilize the enol tautomer, increasing the regioselectivity of this cyclization towards the formation of product **22q**. The reactivity of *N*-aryl hydrazines **23** was affected by electronic effect. The electron-rich system (R = Me) afforded the respective product **22r**, at 69% yield, whereas the electron-deficient one (R = Cl) gave **22s** at 44% yield, requiring a temperature increase from 50 °C to 100 °C. However, despite this significant difference, in comparison with the absence of substituents (product **22o**), the reaction efficiency was remarkably decreased. On the other hand, the results suggest that the process is not sensitive to the electronic effect in the diaryl diselenides **21**, and the products **22u-x** were obtained in good to excellent yields. Additionally, dibutyl and 2,2′-dipyridyl diselenides **21** reacted smoothly to produce the products **22y** and **22z** at a 90% and 58% yield, respectively (Scheme 15).

**Scheme 16:**
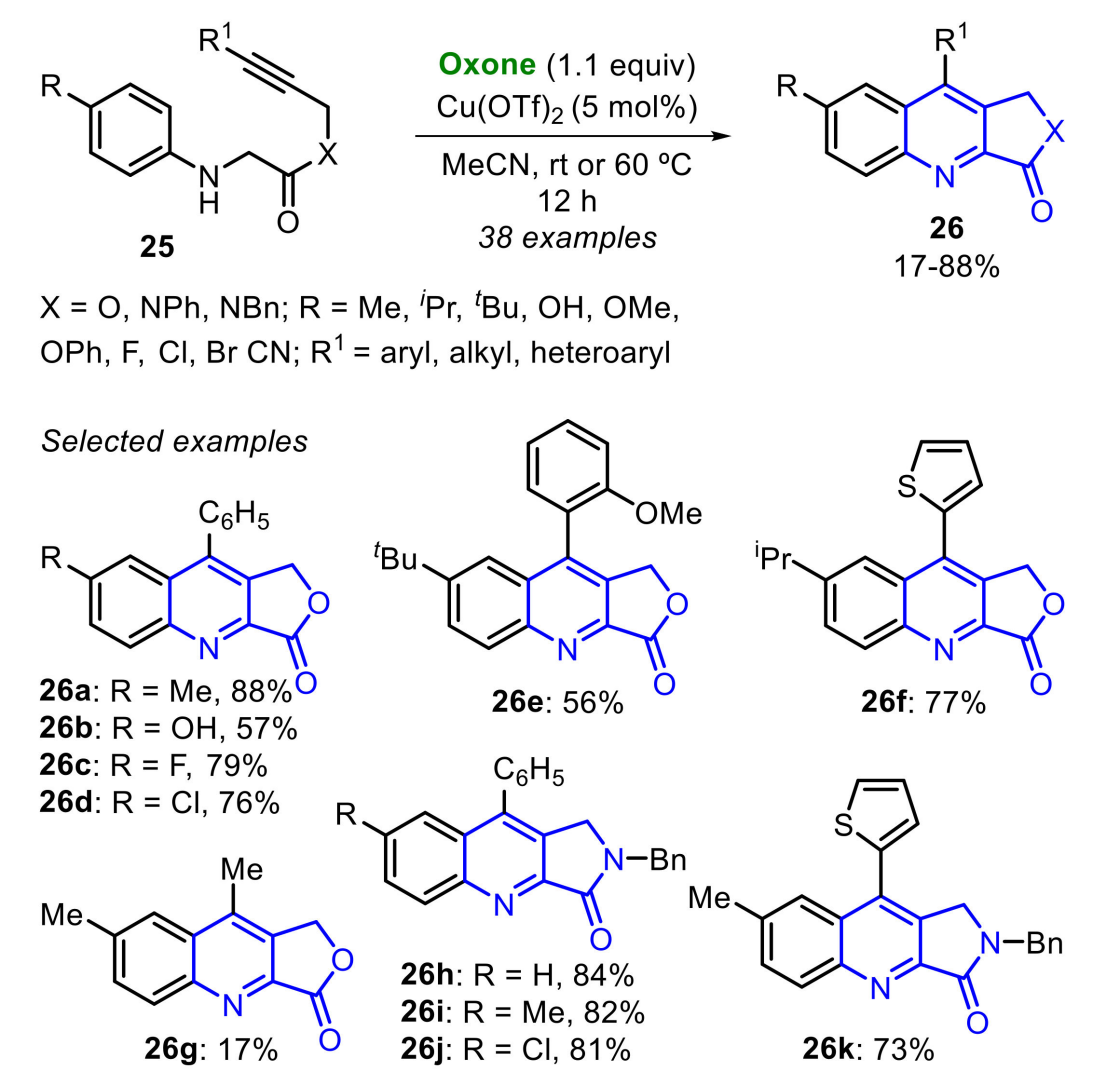
Oxone-promoted intramolecular dehydrogenative Povarov cyclization.

Still in 2019, Muthukrishnan and co-workers reported an Oxone-promoted intramolecular dehydrogenative Povarov cyclization of alkyne tethered *N*-aryl glycine esters and amides **25** (Scheme 16) [24]. Under the optimal conditions (Oxone (1.1 equiv), Cu(OTf)_2_ (5 mol%) in MeCN at room temperature or 60 °C for 12 h), a library of thirty-eight functionalized quinoline-fused lactones and lactams **26** were accessed at up to 88% yield.

**Scheme 17:**
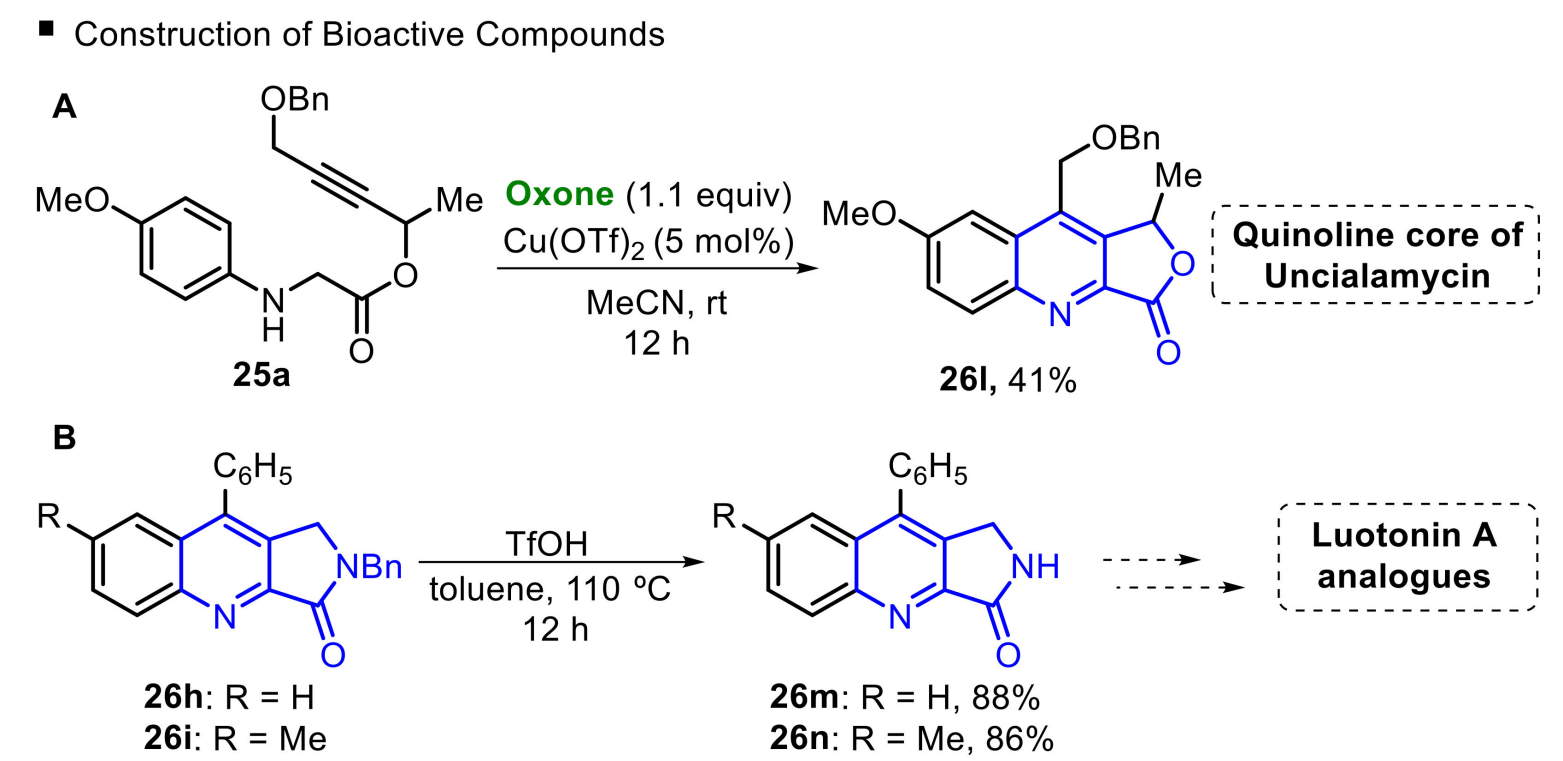
Synthetic application to construct complex structures, such as (**A**) the antibiotic Uncialamycin **26l** and (**B**) the analogues of the cytotoxic alkaloid Luotonin A **26m** and **26n**.

This methodology was used to construct complex structures, such as the antibiotic Uncialamycin **26l** (Scheme 17A). Moreover, the synthesized products **26h** and **26i** were easily converted to **26m** and **26n**, which are analogues of the cytotoxic alkaloid Luotonin A (Scheme 17B).

**Scheme 18:**
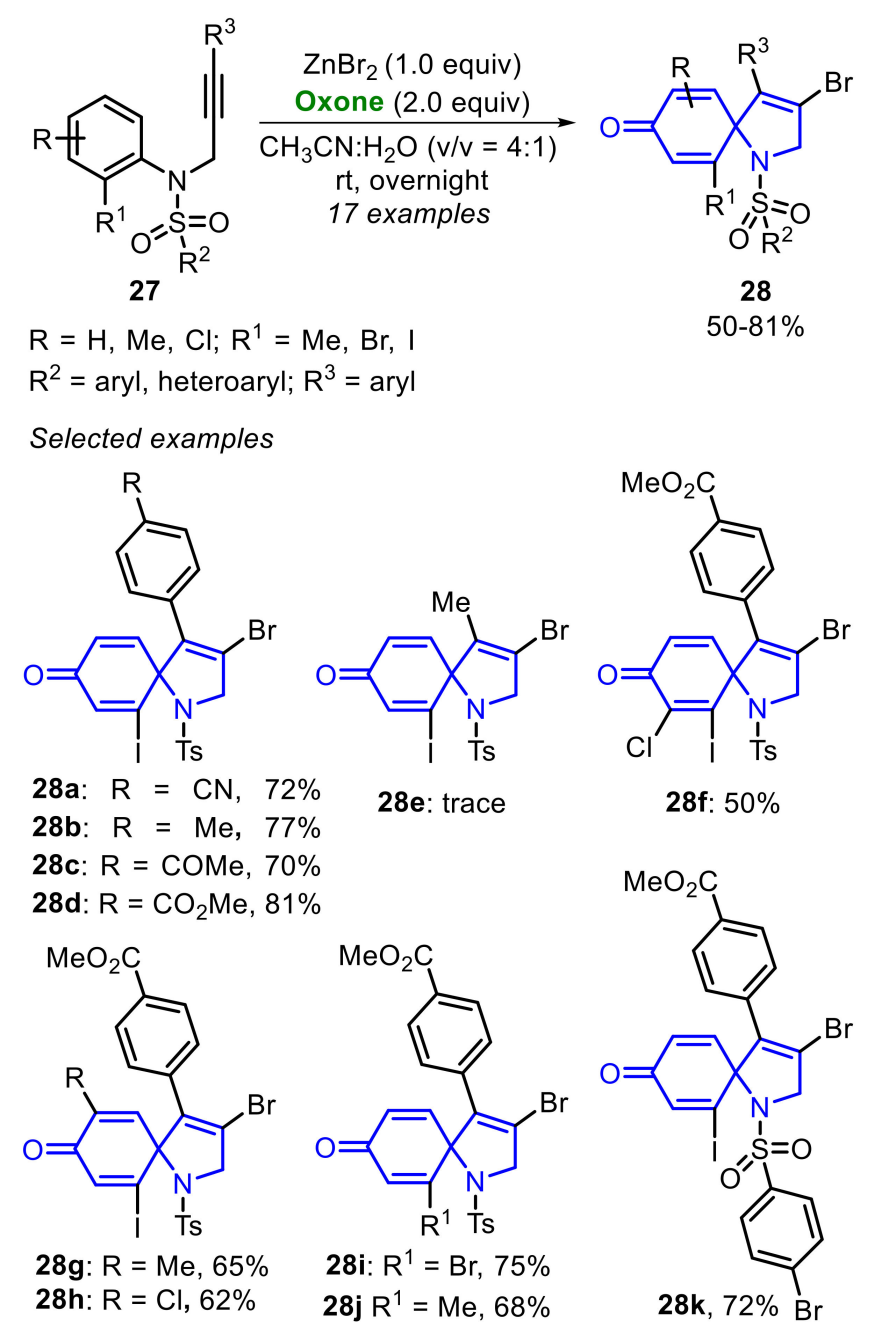
ZnBr_2_/Oxone-mediated radical *ipso*-cyclization of alkynyl anilines **27**.

Liu, Qiu, and co-workers reported the ZnBr_2_/Oxone-mediated radical *ipso*-cyclization of *N*-(3-arylprop-2-yn-1-yl)aniline **27** to prepare 1-azaspiro[4.5]deca-3,6,9-trien-8-ones **28** (Scheme 18) [25]. The reactions were carried out in the presence of ZnBr_2_ (1 equiv), Oxone (2 equiv) and a mixture of acetonitrile and H_2_O (*v*/*v* = 4:1) as solvent at room temperature. In general, electron-rich and electron-deficient aryl groups attached to the C*sp*–C*sp* bond (R = CN, Me, COMe and CO_2_Me) were well tolerated under the optimized condition, affording the products **28a**–**d** in good yields. However, limitations were faced by employing the 2-iodo-*N*-tosyl-*N*-(but-2-yn-1-yl)aniline **27e** as substrate, and the product **28e** was observed only in trace amounts. Regarding the pendent *N*-aryl group, the presence of both electron donor and electron withdrawing substituents (R = Me and Cl) and (R^1^ = Me and Br) were tolerated, affording the products **28g–j** in good yields. A good result was obtained when the *N*-protecting group R^2^ = 4-BrC_6_H_4_ was used, and product **28k** was obtained at a 72% yield (Scheme 18). 

**Scheme 19:**
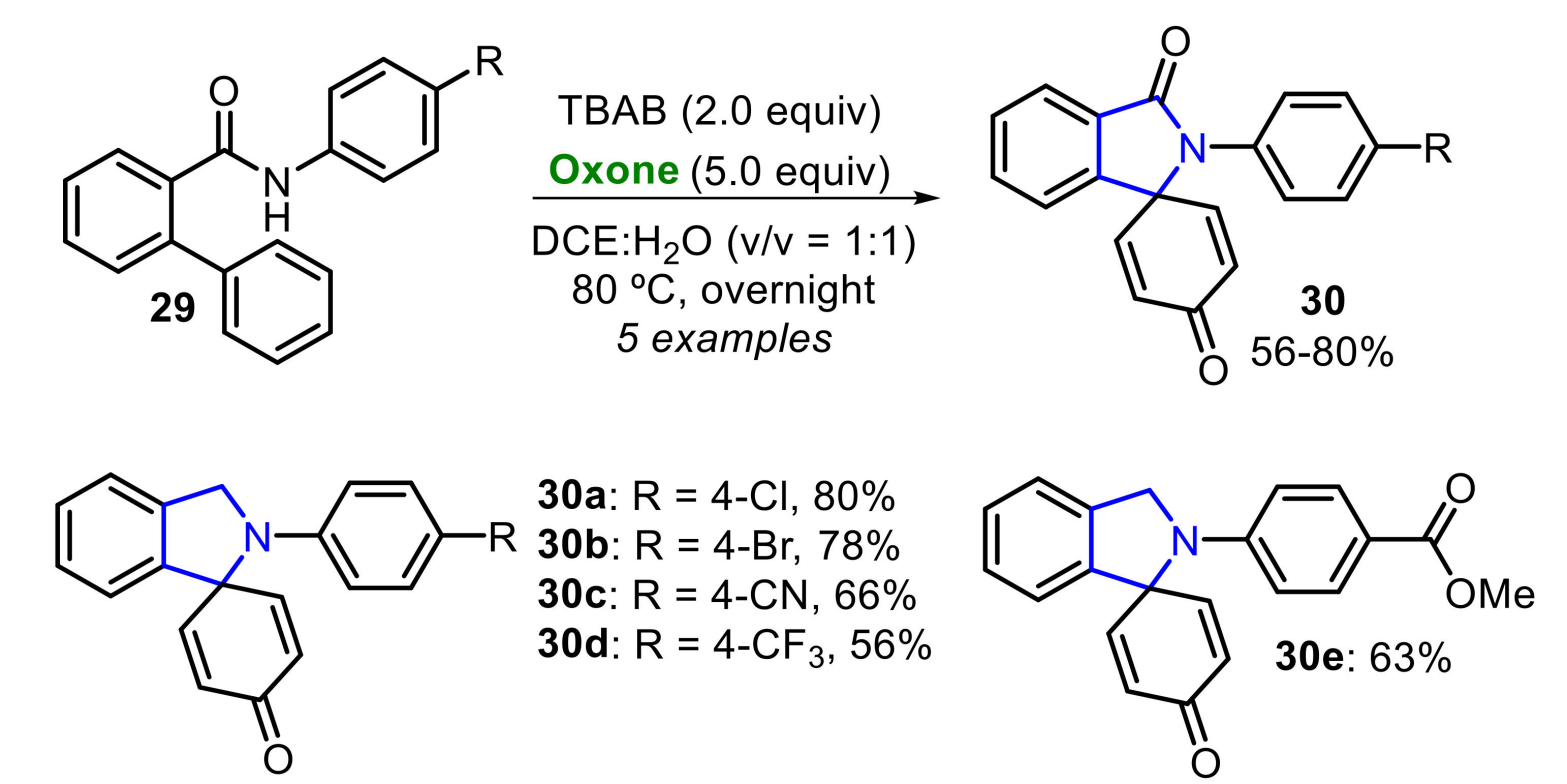
Oxone/TBAB-mediated oxidative cyclization of *N*-aryl-2-arylbenzamides **29**.

Zhou and co-workers reported an Oxone/TBAB-mediated oxidative regioselective cyclization of *N*-aryl-2-arylbenzamides **29** to prepare spiro[cyclohexane-1,1′-isoindoline]-2,5-diene-3′,4-diones **30** (Scheme 19) [26]. A total of five *para*-substituted benzamides **29** (R = Cl, Br, CN, CF_3_, CO_2_Me) were employed as substrate, affording the respective products **30a**–**e** in moderate to good yields.

**Scheme 20:**
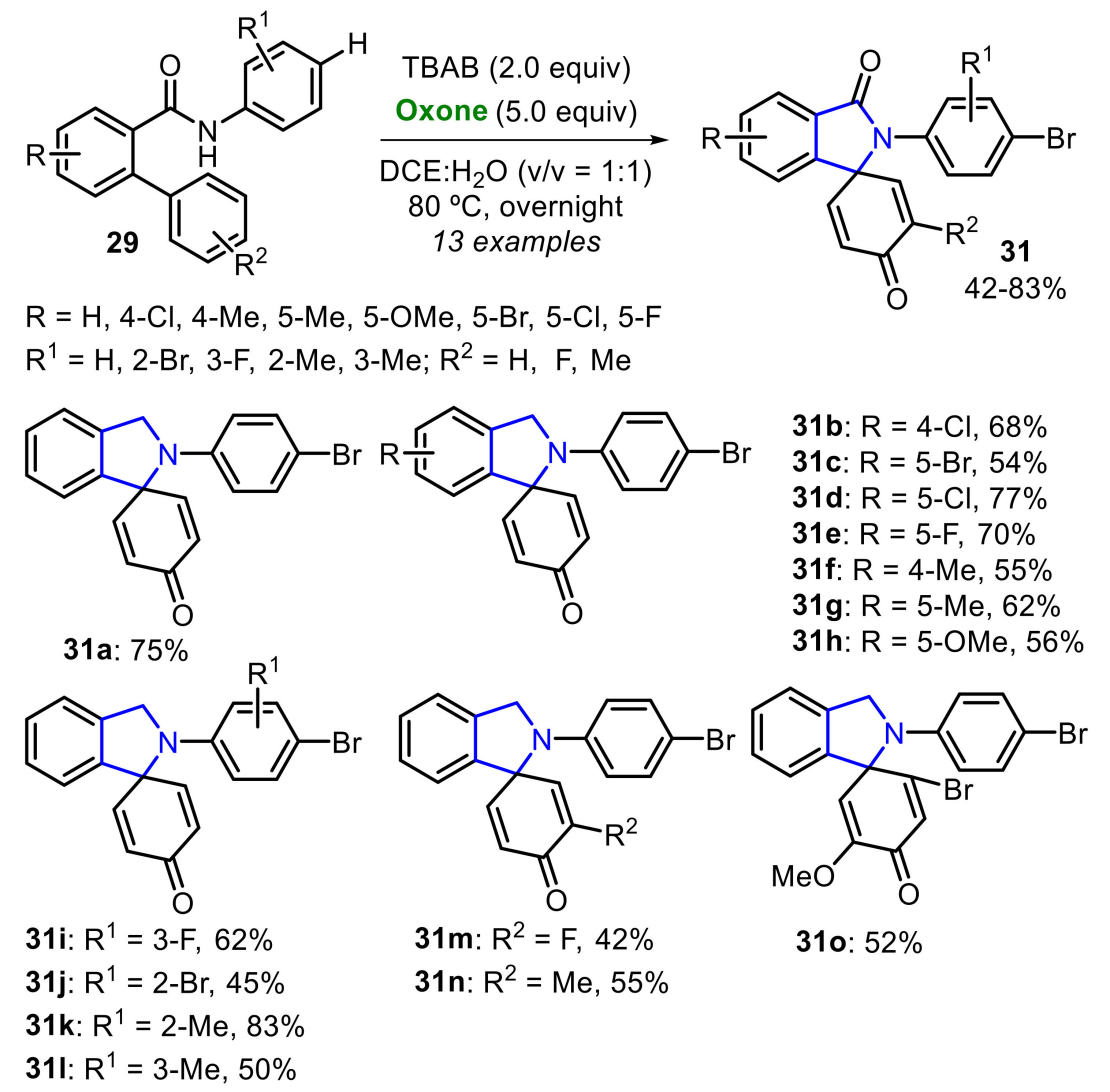
Oxone/TBAB-mediated synthesis of brominated products **31**.

In the absence of a substituent at the *para*-position of the *N*-aryl-2-phenylbenzamide **29**, the bromination of this position occurred, and the product **31a** was afforded in 75% yield. Considering the synthetic versatility of the aryl bromide core, the authors expanded the reaction scope to prepare several brominated products **31** in moderate to good yields (Scheme 20). Interestingly, the presence of a methoxy group in the *ortho*-phenyl ring (R^2^ = 3-OMe) caused the formation of the dibrominated product **31o** at a 52% yield.

**Scheme 21:**
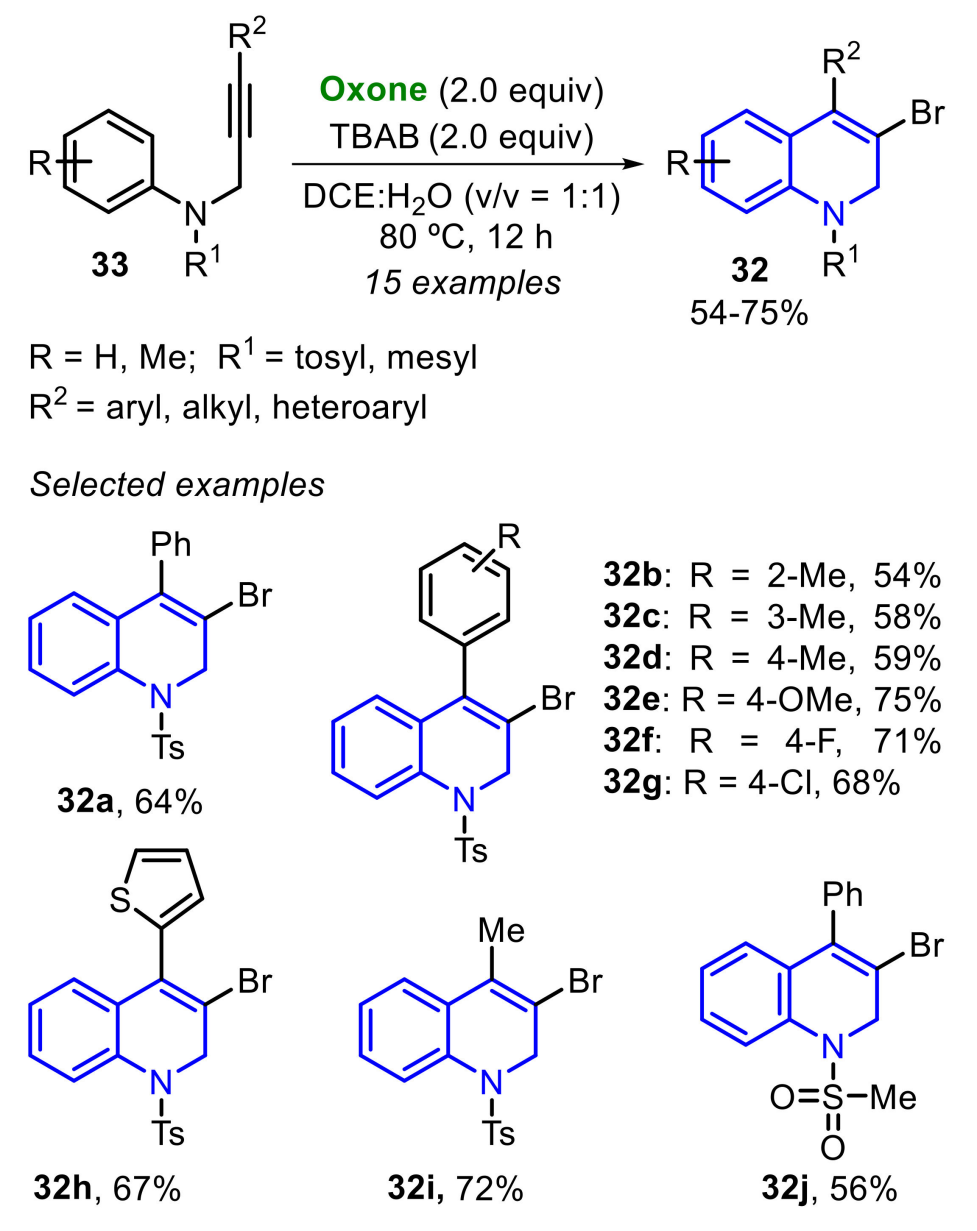
Oxone/TBAB-mediated synthesis of 3-bromo-1,2-dihydroquinoline derivatives **32**.

An Oxone/TBAB-mediated synthesis of 3-bromo-1,2-dihydroquinoline derivatives **32** through a radical 6-*endo*-*trig ortho*-cyclization of 2-alkynylbenzamide **33** was described in 2019 by Liu and co-workers (Scheme 21) [27]. Several substituted *N*-alkynyl-benzenesulfonamides **33** were used under optimal conditions, giving a range of dihydroquinoline derivatives **32** in moderate to good yields. 2-Alkynylbenzamides containing electron-rich benzene ring (R^2^ = Me and OMe) attached to the alkynyl portion were satisfactorily employed as substrate. Among them, the steric hindrance promoted by the methyl group (*ortho*-, *meta*- or *para*-position) slightly affected the reaction efficiency, giving the products **32b**–**d** in similar yields. On the other hand, the presence of the methoxy group at the *para*-position activated the alkynyl portion, affording the product **32e** at a 75% yield, remarkably increasing the reaction efficiency. The protocol presented an interesting halogen tolerance, affording the desired products **32f** (R = 4-F) and **32g** (R = 4-Cl) at 71% and 68%, respectively. Heteroarene- and alkyl-substituted alkynylbenzamides (R^2^ = 2-thienyl and Me), as well as *N*-mesyl, were suitable substrates, affording the respective products **32h**, **32i** and **32j** at 67%, 72% and 56% yields (Scheme 21).

**Scheme 22:**
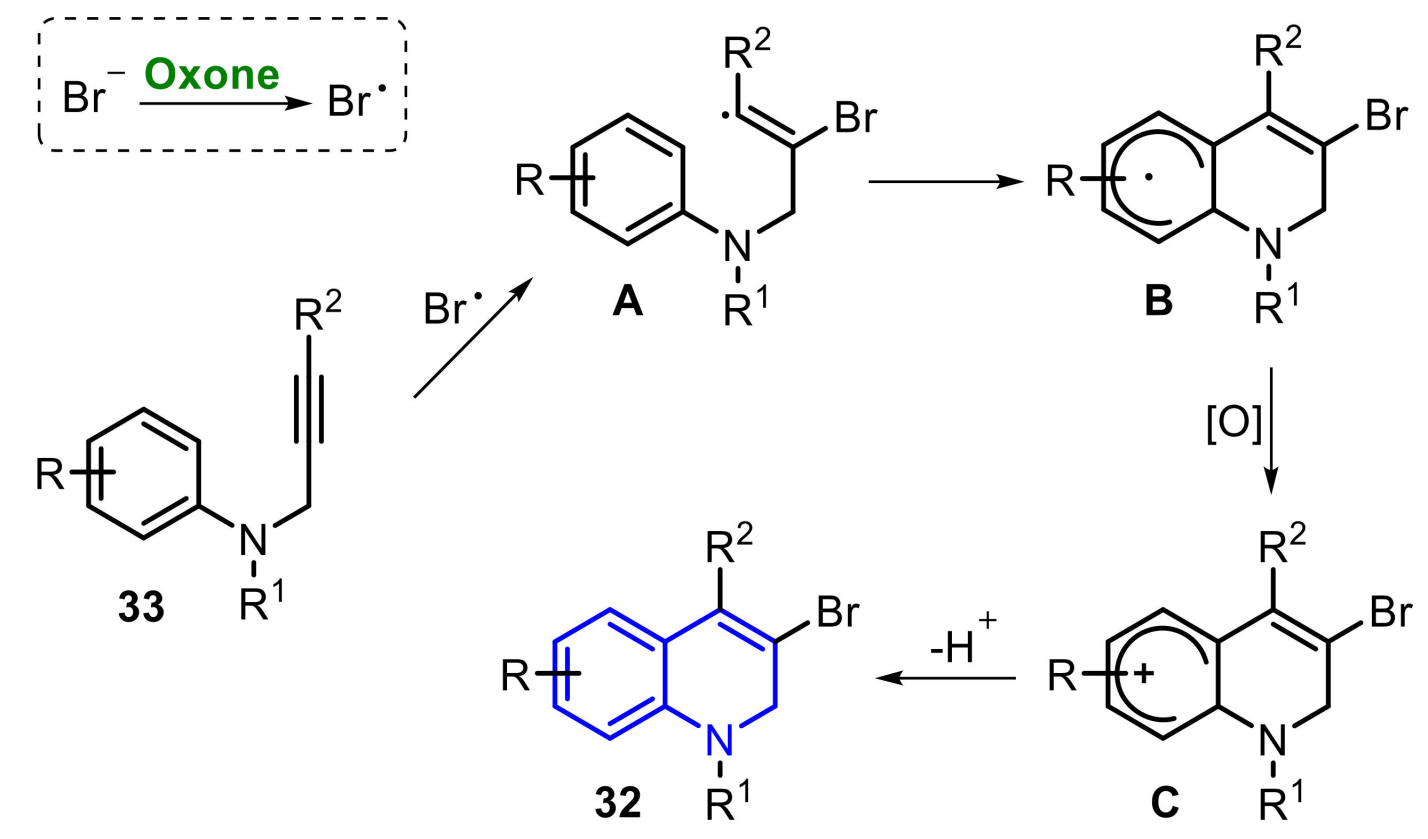
Plausible mechanism for the radical 6-*endo*-*trig* cyclization of the substrate **33**.

Based on control experiments, a plausible reaction mechanism was proposed, which starts with an Oxone-promoted oxidation of bromide anion (Br^–^) to the bromo radical species (Br•) through a SET process. Following on from this, a radical addition of the bromo radical to the substrate **33** produces the intermediate **A**, which undergoes a radical 6-*endo*-*trig* cyclization, to be converted to the intermediate **B**. Finally, the intermediate **B** undergoes oxidation, followed by a deprotonation, to be converted to the desired dihydroquinoline **32** (Scheme 22).

**Scheme 23:**
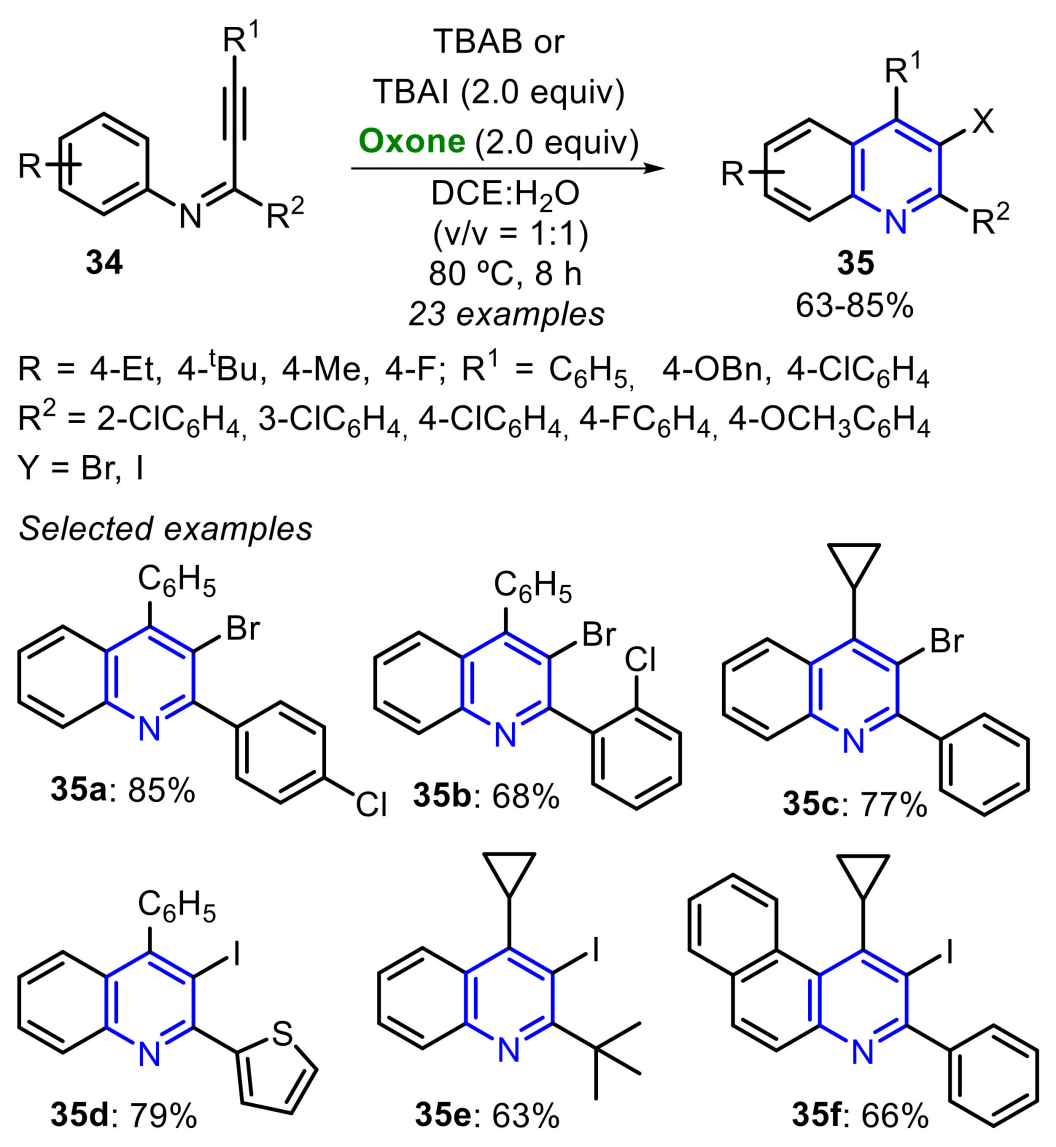
Oxone/TBAX-promoted tandem annulative radical halogenation of alkynyl imines.

In 2020, Qiu and co-workers described an Oxone/TBAX-promoted (X = Br or I) tandem annulative radical halogenation of alkynyl imines **34** to access 3-haloquinolines **35** regioselectively (Scheme 23) [28]. Comparing TBAB and TBAI acting as the halogen-source, any remarkable difference in the reaction efficiency was observed, with TBAB affording 3-bromoquinolines **35** and TBAI delivering 3-iodoquinolines **35** in good to very good yields.

**Scheme 24:**
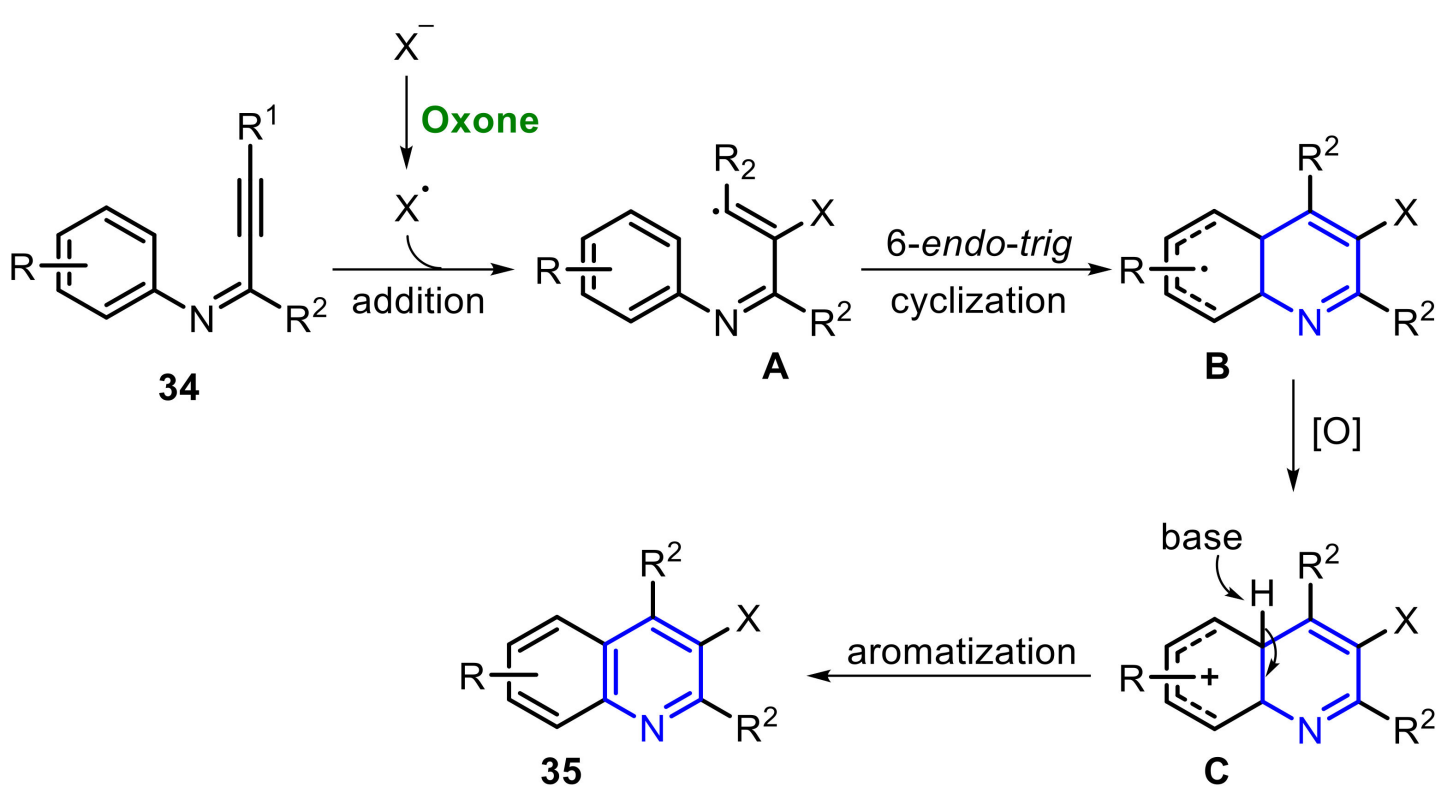
Reaction mechanism for the Oxone/TBAX-promoted tandem annulative radical halogenation.

The reaction mechanism initiates with the Oxone-promoted oxidation of the halogen ion (X^–^) to the halogen radical species (X•), that is subsequently added to the C≡C bond, to produce the vinyl radical intermediate **A**. In the sequence, the cyclized intermediate **B** is obtained via a 6-*endo*-*trig* cyclization, followed by the oxidation to the intermediate **C**, which is finally aromatized to the product **35** (Scheme 24).

**Scheme 25:**
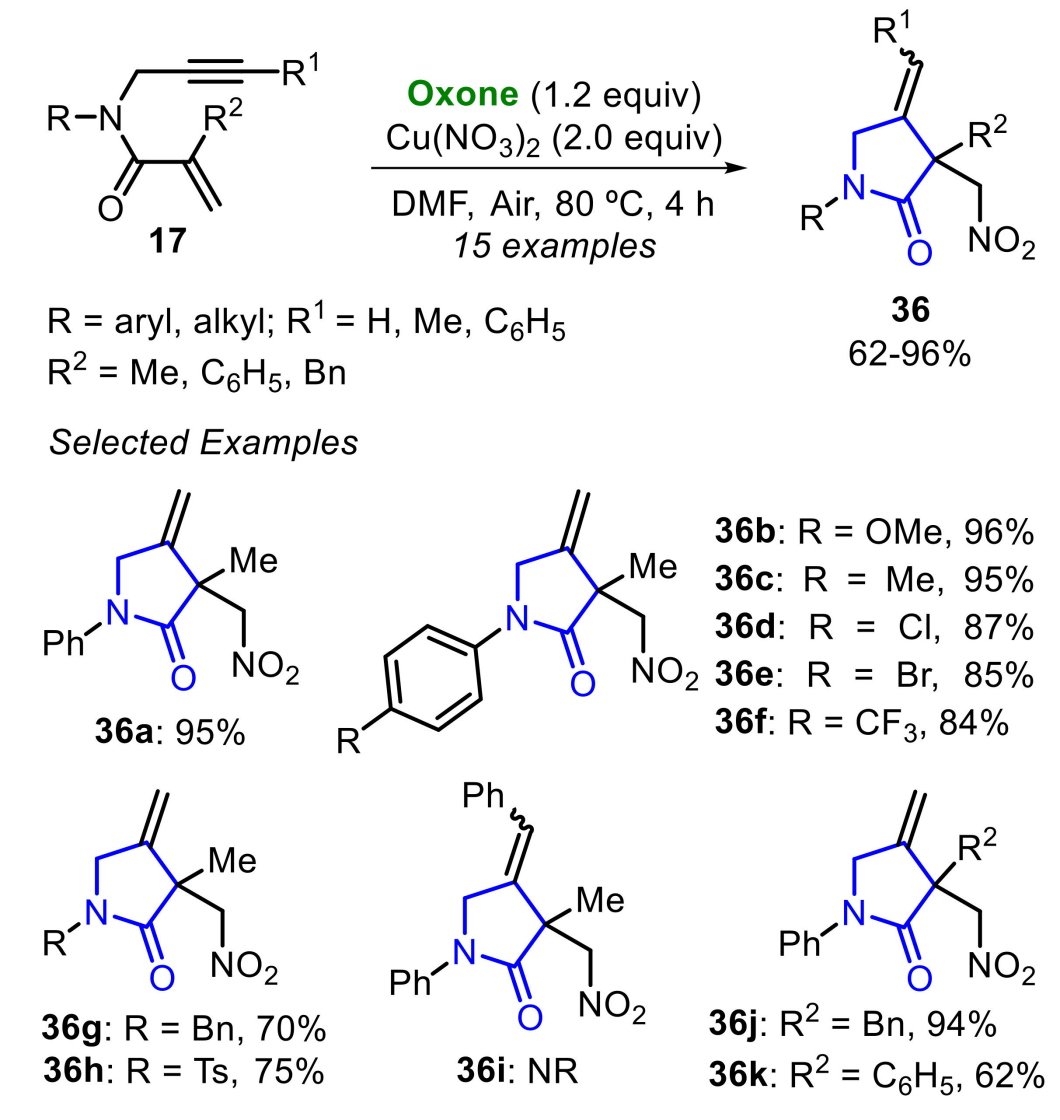
Oxone/Cu(NO_3_)_2_-promoted radical annulative nitration of 1,6-enynes **17**.

In the same year, Wei, Liu, Lei and co-workers reported an Oxone/Cu(NO_3_)_2_-promoted radical annulative nitration of 1,6-enynes **17** (Scheme 25) [29]. Under the optimized reaction conditions, several *N*-substituted 1,6-enynes **17**, bearing different functional groups in the alkynyl and alkenyl portions, reacted satisfactorily to produce nitro-containing pyrrolidin-2-ones **36** in good to excellent yields. It is worth to mention that the protocol was satisfactorily scalable to 10 mmol, affording 3-methyl-4-methylene-3-(nitromethyl)-1-phenylpyrrolidin-2-one **36a** at a 82% yield.

**Scheme 26:**
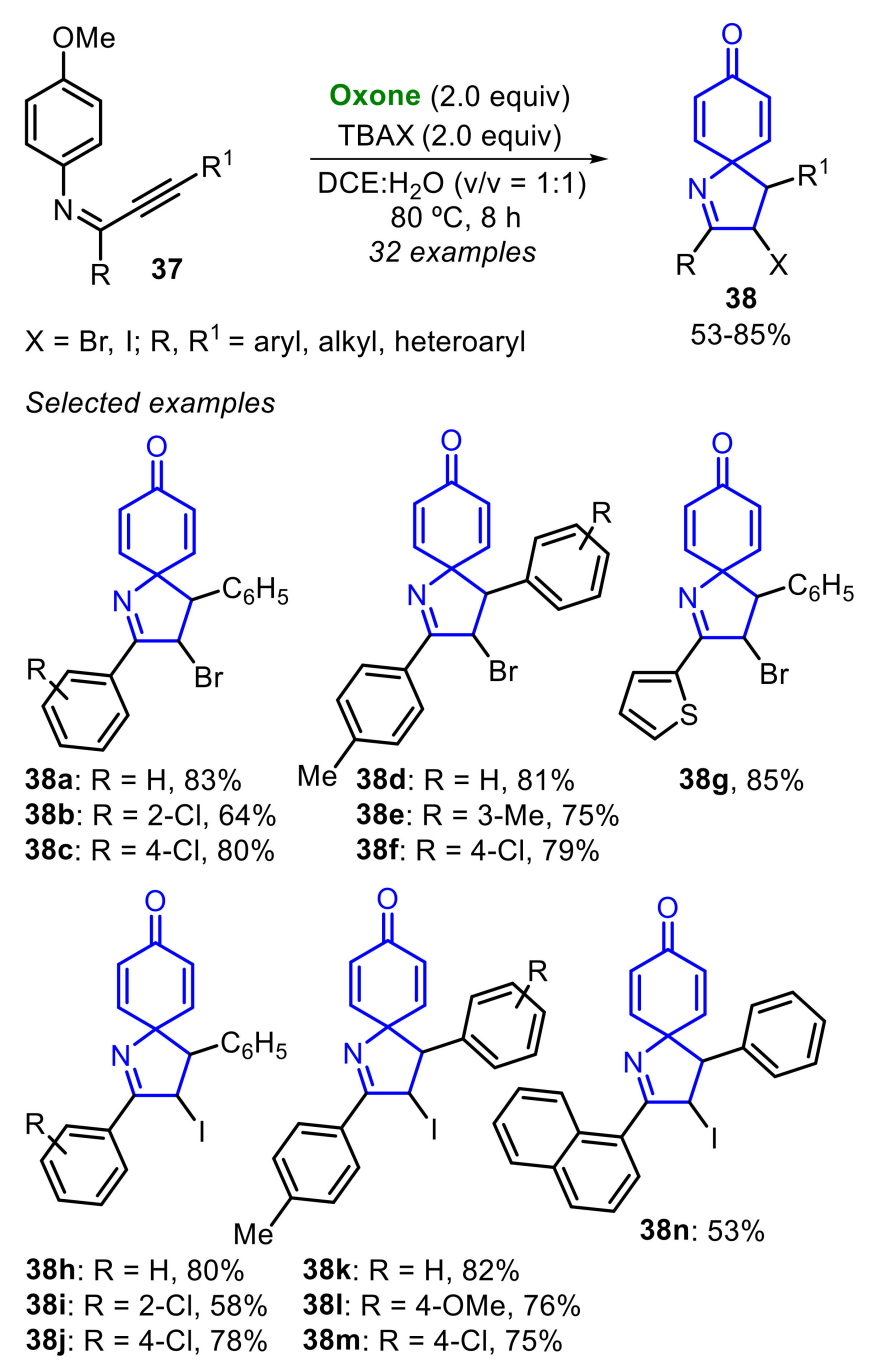
Oxone/TBAX-promoted spirocyclization of *p*-methoxyl alkynyl-*N*-phenylimines **37**.

Qiu, Shan, Chen and co-workers also reported a protocol based on the generation of halogen radical species (X•), employing the system Oxone/TBAX (X = Br and I), which disclosed the radical annulation of *para*-methoxyl alkynyl-*N*-phenylimines **37**, to access 3-halospirocyclohexadienones **38** (Scheme 26) [30]. Instead of the expected 6-*endo*-*trig* cyclization (Scheme 24), a radical 5-*exo*-*trig* cyclization occurred, disclosing a dearomative aza-spirocyclization, driving to the spirocyclic system **38**. Through an optimization study, the best reaction condition was set reacting the substrate **36** in DCE:H_2_O (*v*/*v* = 1:1), at 80 °C for 8 h, in the presence of Oxone (2.0 equiv) and TBAB or TBAI (2.0 equiv). In general, several *para*-methoxyl alkynyl-*N*-phenylimines **37** were satisfactorily submitted to the optimized reaction conditions, tolerating a wide range of substituents, including aryl systems bearing electron-donating and -withdrawing groups, heteroaryl and alkyl (Scheme 26). 

**Scheme 27:**
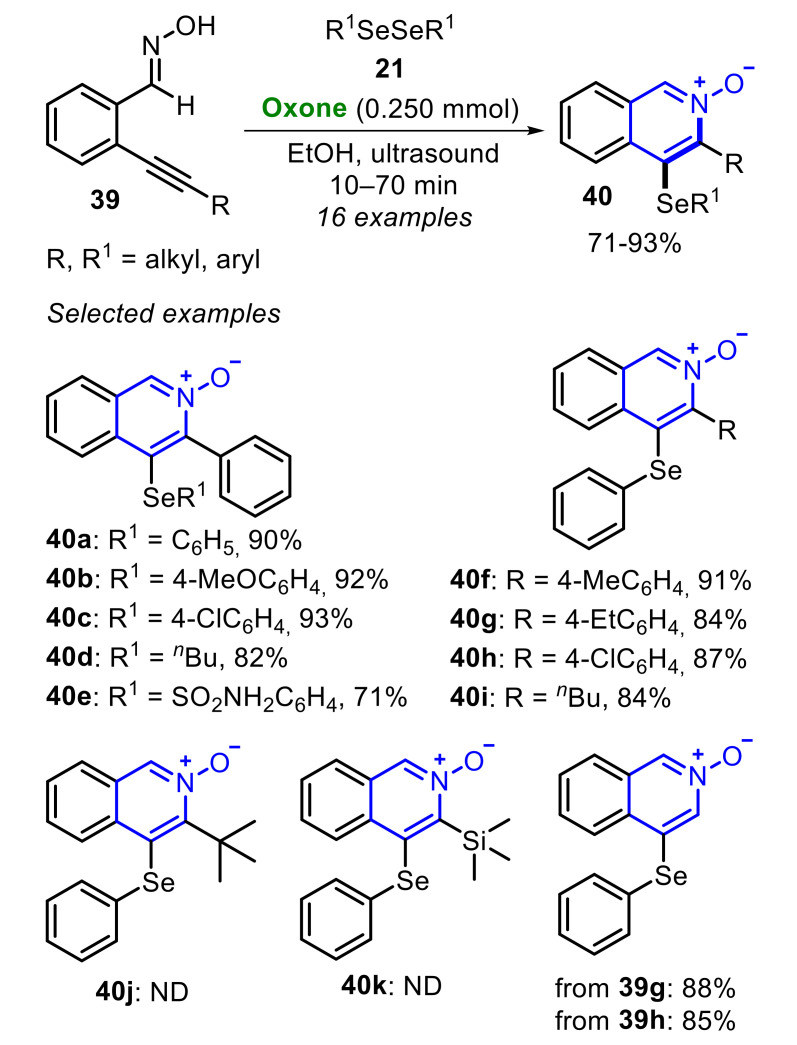
Oxone/RSeSeR-promoted electrophilic cyclization of alkynylbenzaldoximes **39**.

In 2021, some of us reported the Oxone/RSeSeR-promoted electrophilic *6-endo-dig* cyclization of alkynylbenzaldoximes **39**, to access 3-organyl-4-(organylchalcogenyl)isoquinoline-2-oxides **40** (Scheme 27) [31]. The process was conducted under ultrasound irradiation in the presence of EtOH as promoting reaction medium, affording twenty-one Se-containing *N*-oxides **40** at good to excellent yields, within just a few minutes of reaction. Diaryl diselenides bearing electron-donating (R^1^ = 4-MeOC_6_H_4_) and -withdrawing groups (R^1^ = 4-ClC_6_H_4_) reacted smoothly to produce the products **40b** and **40c** at excellent yields. Dialkyl (R^1^ = *^n^*Bu) and the sulfonamide-derivative diselenide (R^1^ = SO_2_NH_2_C_6_H_4_) were suitable substrates, affording the respective isoquinoline-2-oxides **40d** and **40e** in 82% and 71% yield. In general, several electron-rich and electron-deficient alkynylbenzaldoximes **39** were satisfactorily submitted to the optimal conditions. However, limitations were found when an alkyl group (R = *^t^*Bu) was attached in the C≡C bond, and the conversion to the expected product **40j** was not observed An interesting result was obtained when the alkynylbenzaldoxime **39g**, bearing the TMS group attached to the C≡C bond (R = Si(Me)_3_) was employed as substrate. In this case, the expected product **40k** was not afforded, and the reaction was driven to the C3-free product **40l**, obtained in 88% yield. The same product **40l** could be accessed from the unprotected terminal alkynylbenzaldoxime **39h** at an 85% yield (Scheme 27).

**Scheme 28:**
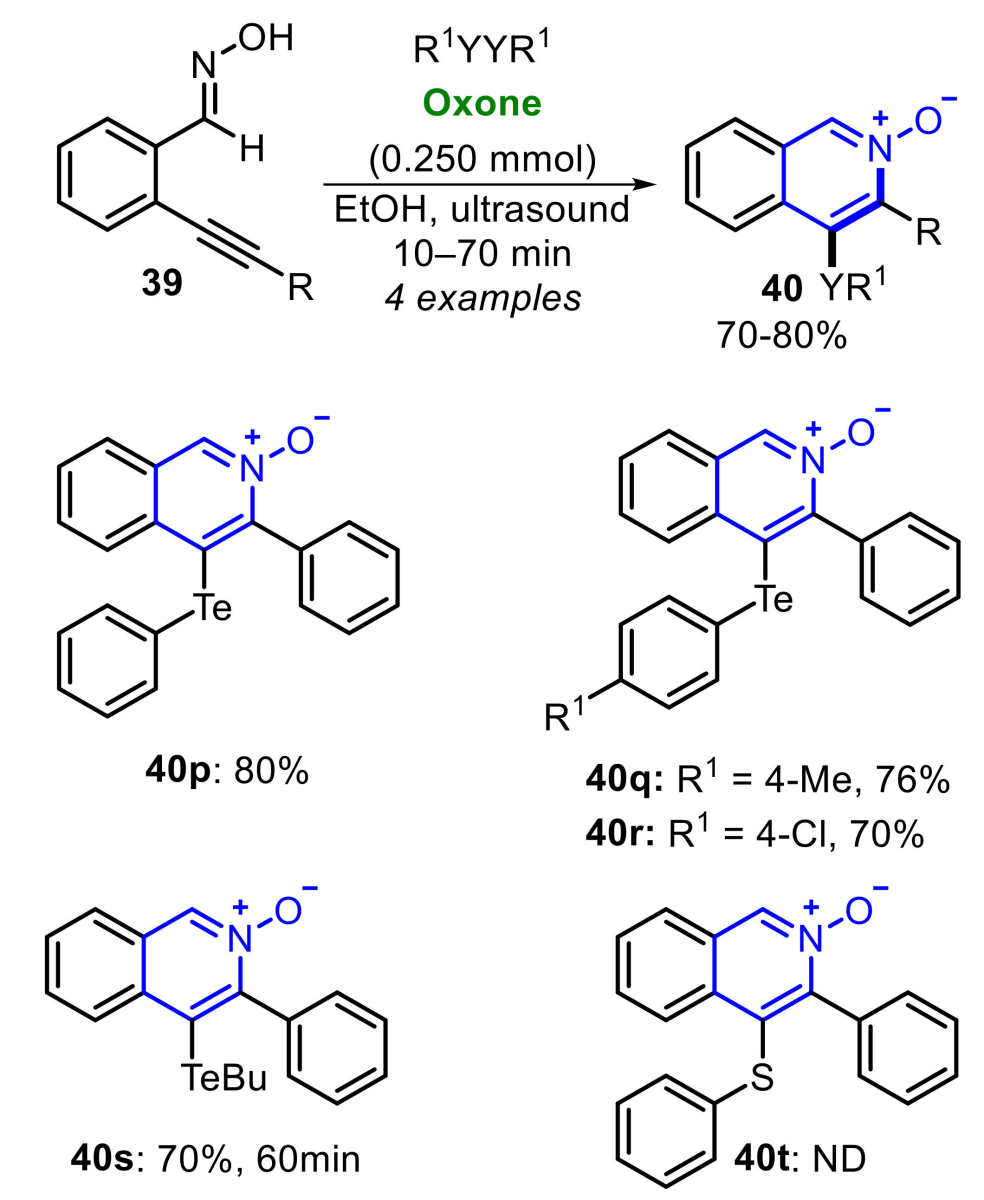
Oxone/RTeTeR-promoted electrophilic cyclization of alkynylbenzaldoximes **39**.

The protocol was satisfactorily expanded to diaryl ditellurides **41a**–**c** and dibutyl ditelluride **41d**, affording tellurium-functionalized isoquinoline-2-oxides **40p**-**s** in good to very good yields (Scheme 28). No reaction was observed using diphenyl disulfide **42a**, and 4-(phenylthio)isoquinoline-2-oxide **40t**, was not obtained, even after 60 min under sonication.

## 3. Oxygen-Containing Heterocycles 

Oxygen-containing heterocycles are an important class of compounds, which are abundantly found in nature, and are especially important in the industry, being widely used as flavor and fragrances [32]. Furthermore, these compounds present an important range of biological activities, including anticancer [33], immunosuppressive and hypolipidemic [34]. As a consequence of the importance of these compounds, several innovative, simple and efficient Oxone-based protocols have been recently developed to synthesize oxygen-containing heterocycles.

**Scheme 29:**
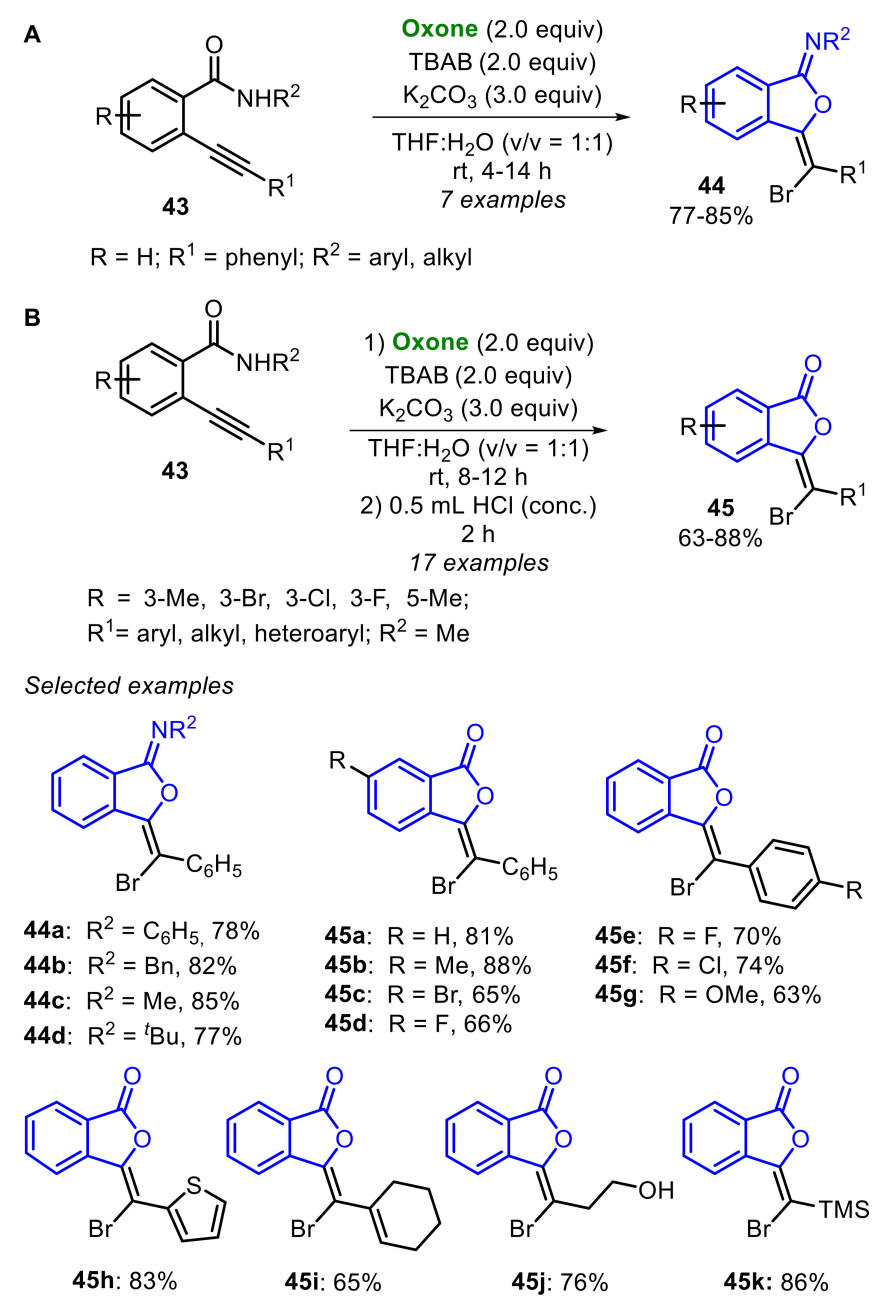
Oxone/TBAB-promoted 5-*exo*-*dig* oxy-cyclization of 2-alkynylbenzamide **43**. (**A**) Synthesis of isobenzofuran-1-imines **44**. (**B**) Synthesis of isobenzofuran **45**.

In 2018, Qiu and co-workers reported the Oxone/TBAB-promoted 5-*exo*-*dig* oxy-cyclization of 2-alkynylbenzamide **43** to access isobenzofuran-1-imines **44** and isobenzofuran **45** selectively, in the presence of K_2_CO_3_ (Scheme 29A,B) [35]. Under the optimized reaction conditions, the product **44** is mostly formed at good to very good yields. However, the product **45** could be accessed at moderate to very good yields using an additional HCl-based hydrolysis step (Scheme 29).

**Scheme 30:**
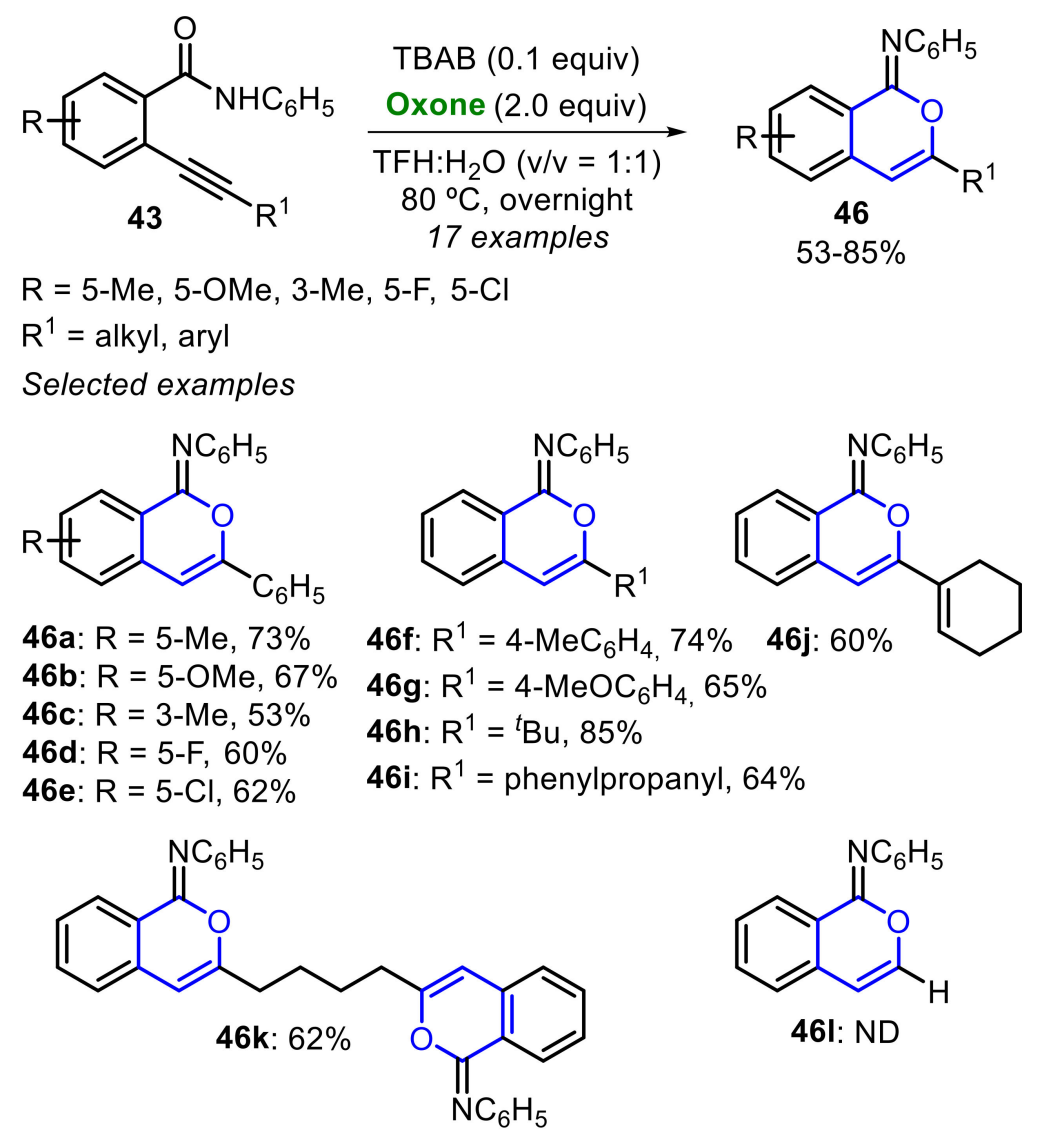
TBAB-catalyzed Oxone-mediated *6-endo-dig* oxidative annulation of 2-alkynylbenzamide **43**.

In 2019, Liu and co-workers described the TBAB-catalyzed Oxone-mediated *6-endo-dig* oxidative annulation of 2-alkynylbenzamide **43** to access several substituted isocoumarin-1-imines **46** (Scheme 30) [36]. Differently substituted 2-alkynylbenzamides **43**, bearing electron-rich and electron-deficient aryl and alkyl groups, were suitably employed as substrates, affording the respective products **46** regioselectively, in moderate do very good yields. Limitations were found when 2-ethynyl-*N*-phenylbenzamide **43l** was employed as substrate, and the respective isocoumarin-1-imine **46l** was not formed.

**Scheme 31:**
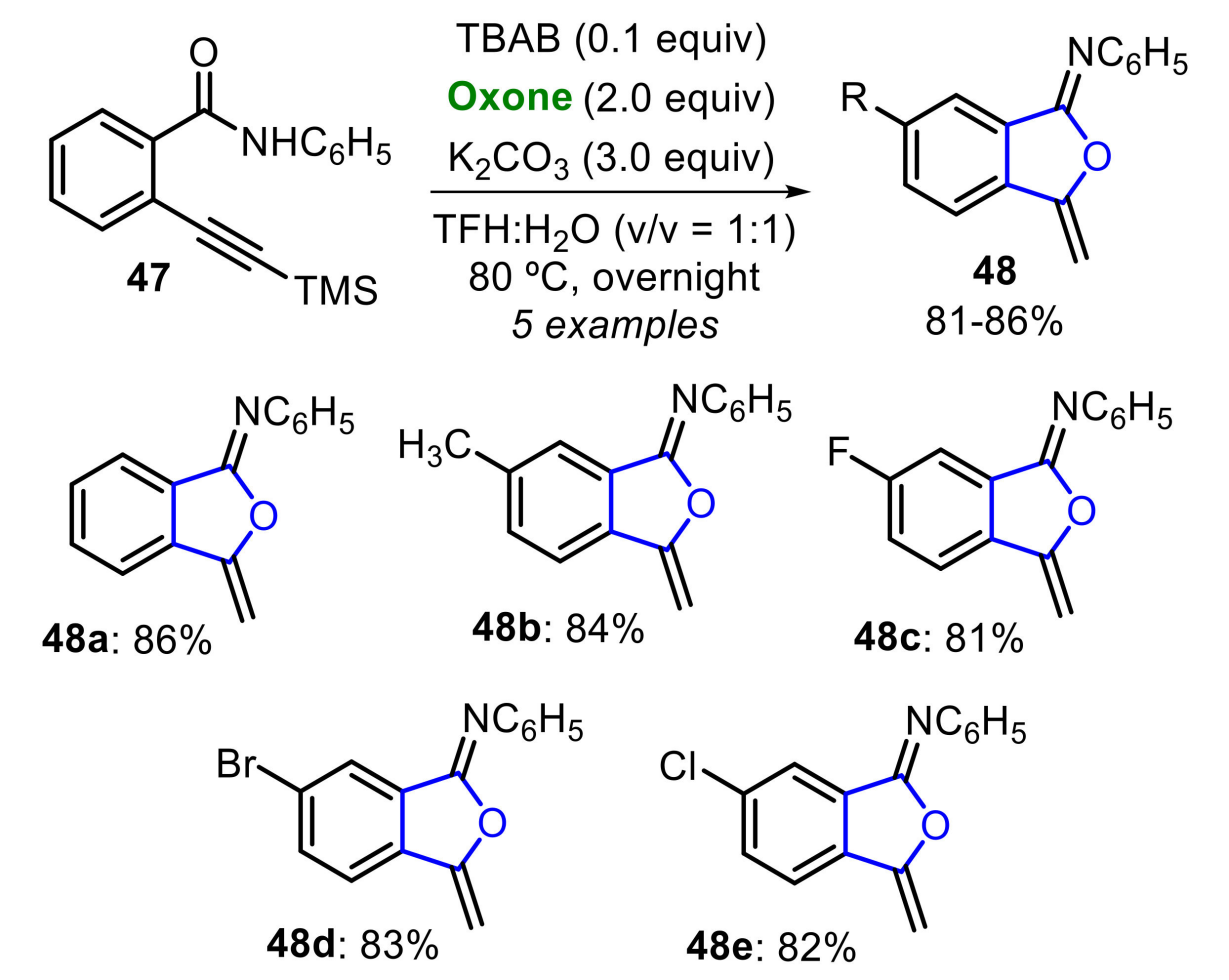
TBAB-catalyzed Oxone-mediated synthesis of isobenzofuran-1-imines **48**.

During their studies, the authors noticed that when the *N*-phenyl 2-trimethylsilylethynylbenzamides **47** was submitted to the optimal reaction conditions, isobenzofuran-1-imines **48** were obtained as the major product, through an oxidative *5-exo-dig* annulation (Scheme 31). Inspired by this result, they decided to investigate the reaction scope, employing several *meta*-substituted *N*-phenyl 2-trimethylsilylethynylbenzamides **47**, bearing electron-donating and electron-withdrawing groups (R = H, Me, F, Br and Cl), and the respective products **48** were obtained in very good yields.

**Scheme 32:**
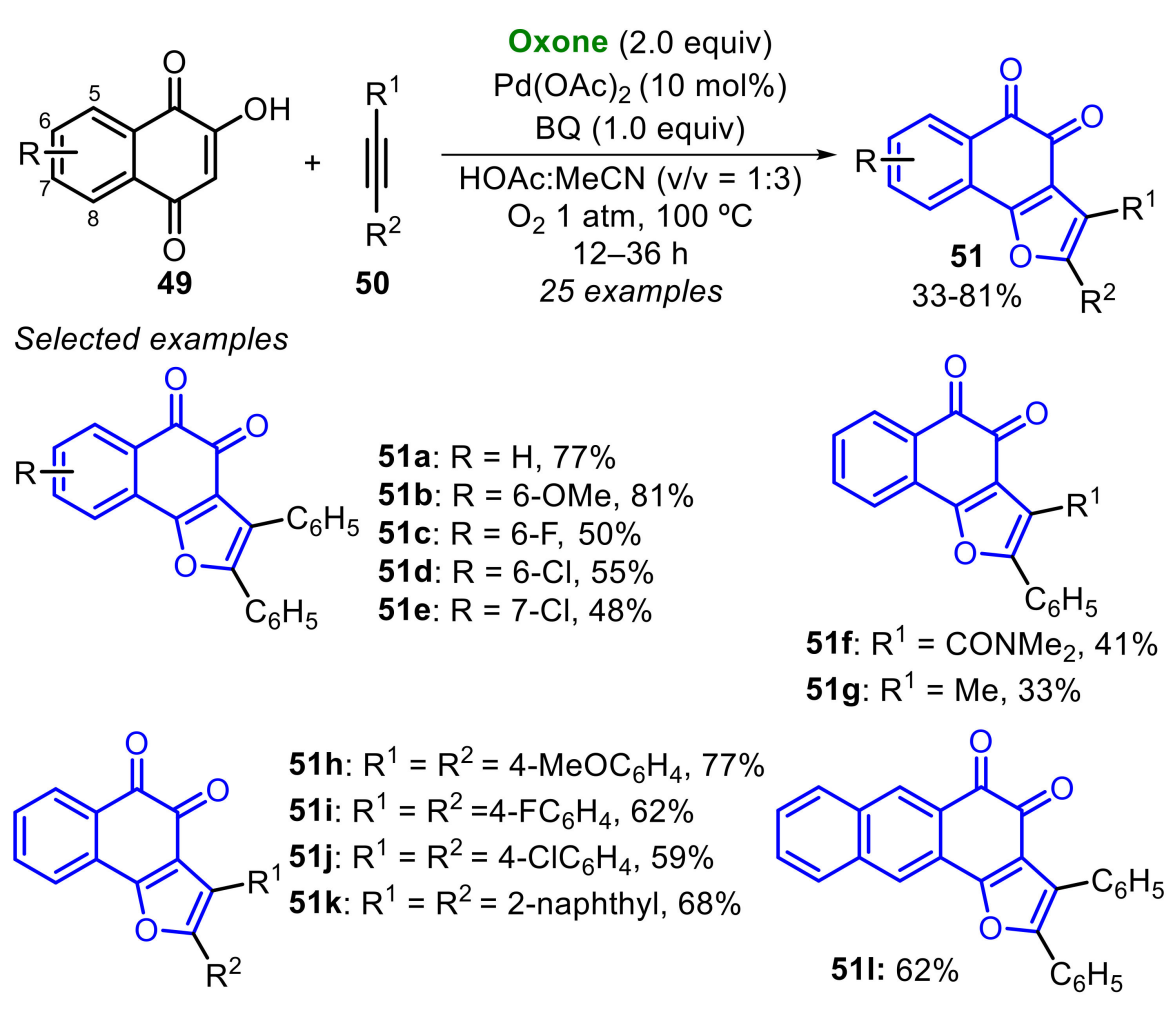
Pd(II)-catalyzed Oxone-mediated oxidative annulation of 1,2-naphthofuroquinone **49** and diaryl alkynes **50**.

In the same year, Wang, Sun, and co-workers reported the Pd(II)-catalyzed Oxone-mediated oxidative sequential addition-annulation of 1,2-naphthofuroquinones **49** with diaryl alkynes **50**, to prepare functionalized naphthofuroquinones **51** (Scheme 32) [37]. The method was efficiently applied to several functionalized 2-hydroxy-1,4-naphthoquinones **49**, bearing electron-neutral, -donating, and -withdrawing aryl substituents. Regarding the diaryl alkyne counterpart **50**, symmetrical and unsymmetrical, as well as electron-rich and electron-deficient derivatives could be converted to the respective naphthofuroquinone **51** in poor to very good yields. The biological potential of the synthesized products was evaluated, and the naphthofuroquinone **51a** presented a potent endothelial protective activity.

**Scheme 33:**
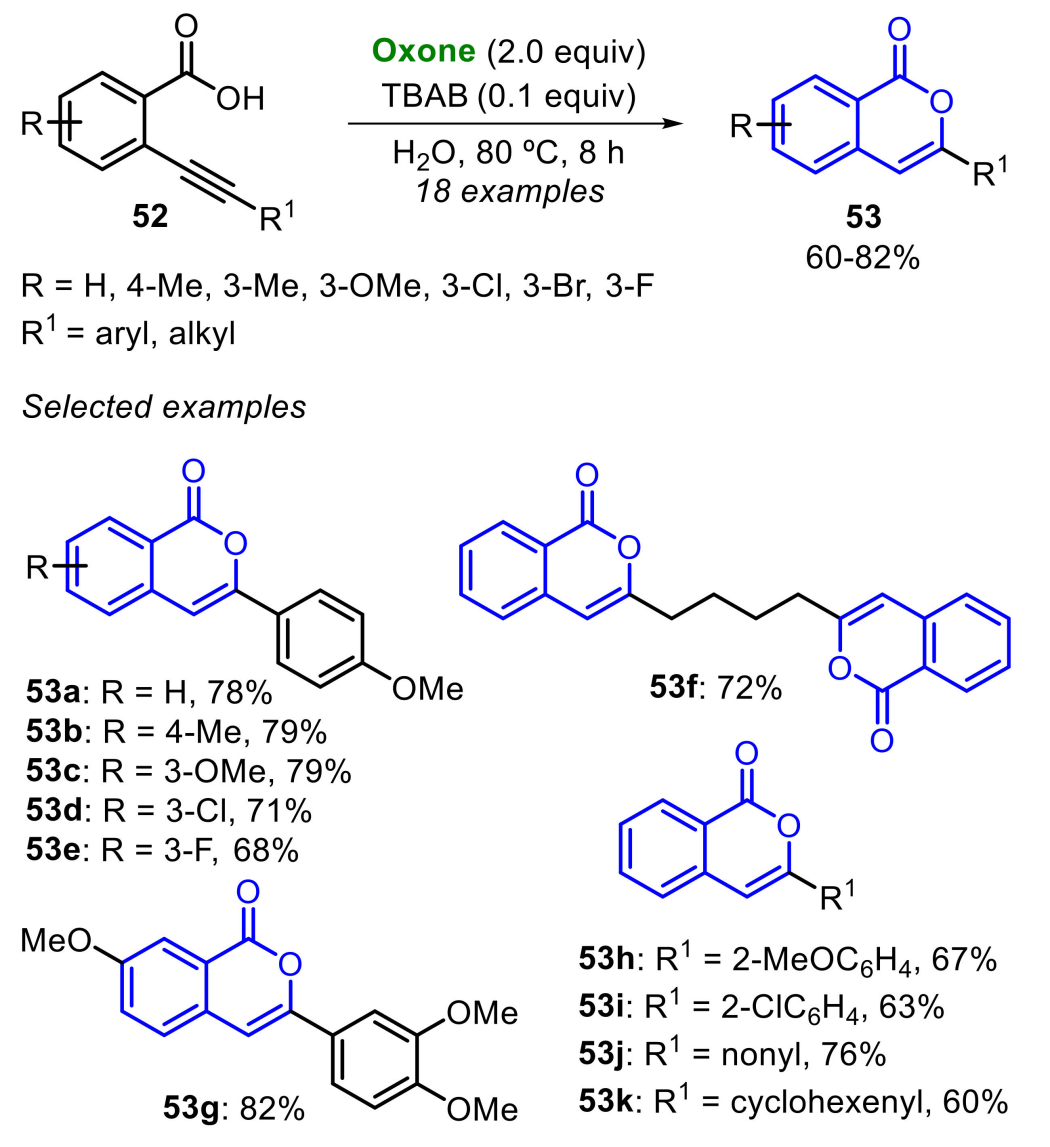
Oxone/TBAB-promoted radical cyclization of 2-alkynylbenzoic acids **52**.

Recently, Liu, Zhou, Qiu and co-workers described the Oxone/TBAB-promoted radical cyclization of 2-alkynylbenzoic acids **52**, to construct isocoumarins **53**, employing catalytic amounts of TBAB (10 mol%) (Scheme 33) [38]. Substituted 2-alkynylbenzoic acids **52** bearing electron-donating and -withdrawing groups were satisfactorily employed, affording a total of eighteen functionalized isocoumarins **53** in moderate to very good yields. Regarding the alkynylbenzoic aryl moiety, when the system was deactivated (R = 3-Cl and 3-F), a slight loss in the reaction efficiency to access the products **53d** and **53e** was faced, whereas electron-donating groups (R = 4-Me and 3-OMe) did not affect the reactivity remarkably. Interestingly, the method was extended to the bis-functionalized substrate 2,2’-(octa-1,7-diyne-1,8-diyl)dibenzoic **52f**, giving the corresponding isocoumarin **53f** in 72% yield. Additionally, 2-alkynylbenzoic acids **52** bearing vinyl and alkyl groups attached in the C≡C bond, reacted smoothly to produce the products **53j**–**k** at good yields (Scheme 33). 

**Scheme 34:**
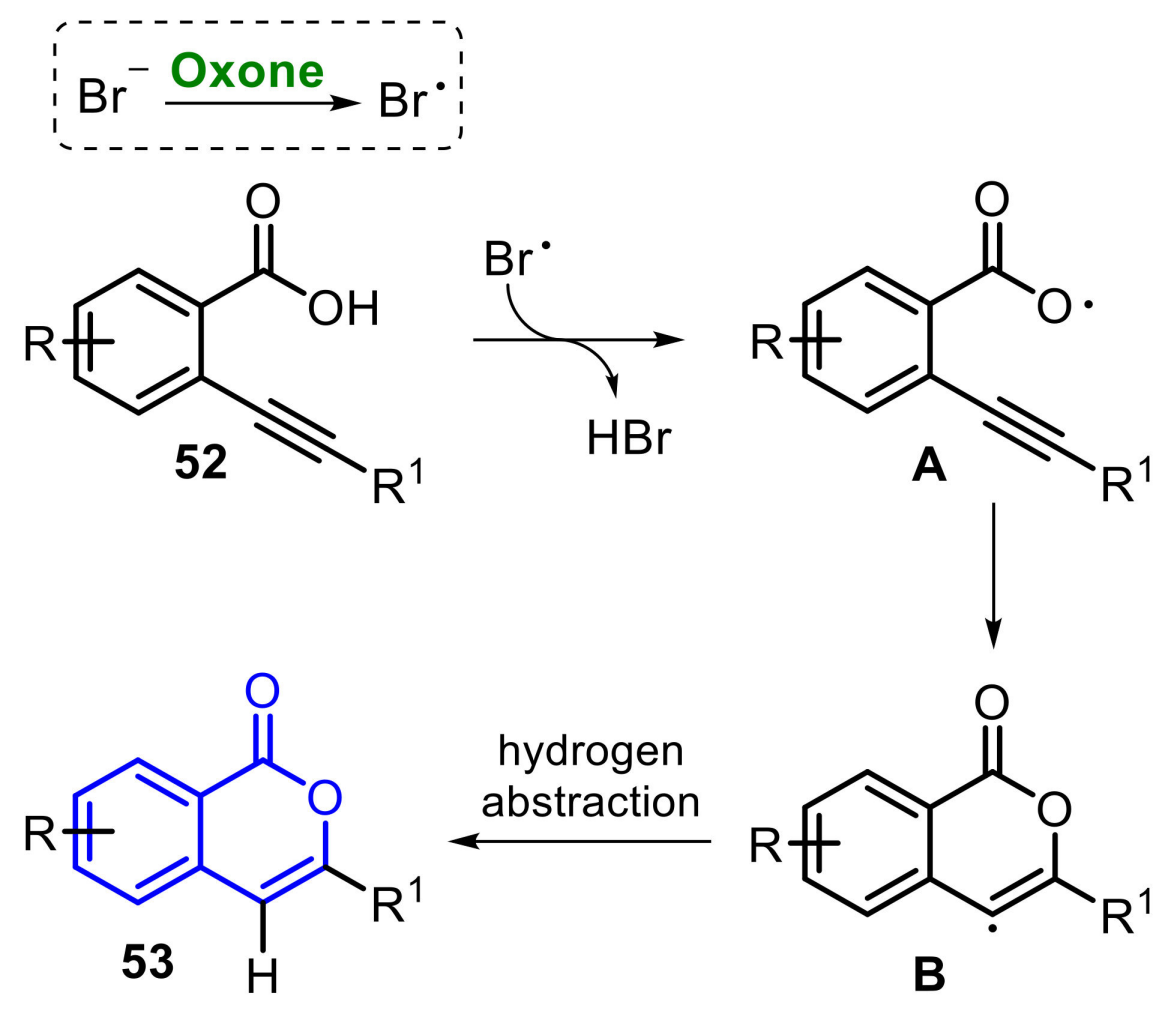
Reaction mechanism of the Oxone/TBAB-promoted radical cyclization of 2-alkynylbenzoic acids **52**.

The first step of the reaction mechanism is the formation of the bromo radical (Br•) species, as proposed in Scheme 21 and Scheme 23. In the sequence, the 2-alkynylbenzoic acid **52** is oxidized, affording the oxygen-centered radical species **A**, which quickly undergoes a *6*-*endo*-*dig* radical annulation to produce the isocoumarin radical **B**. Finally, after a radical hydrogen abstraction, the intermediate **B** is converted to the isocoumarin **53** (Scheme 34).

**Scheme 35:**
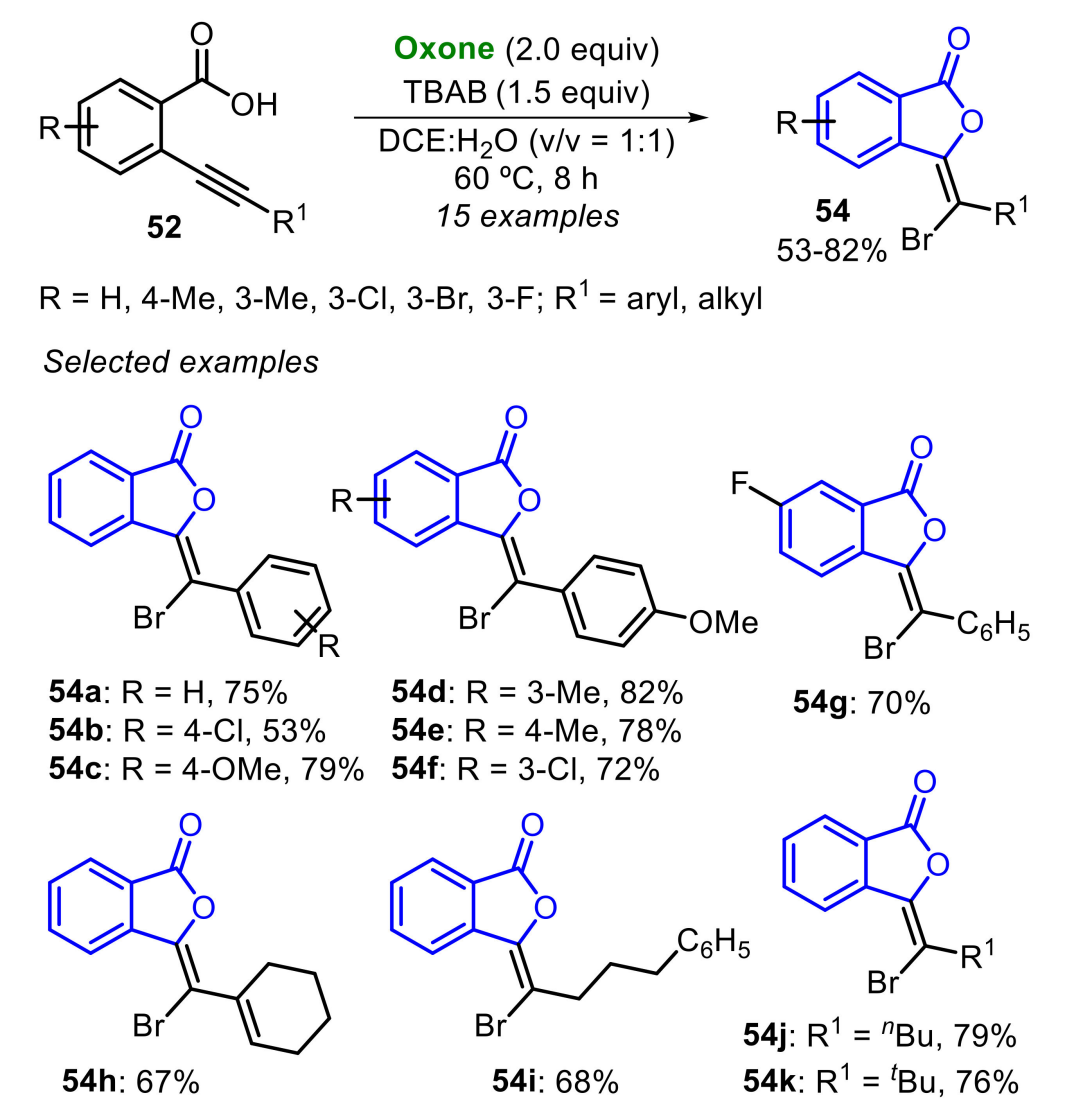
Oxone/TBAB-promoted radical cyclization of 2-alkynylbenzoic acids **52**.

The system TBAB/Oxone was also employed in the 5-*exo*-*dig* bromocyclization of 2-alkynylbenzoic acids **52**, aiming the synthesis of 3-(bromomethylene)isobenzofuran-1(3*H*)-ones **54**, employing overstoichiometric amounts of TBAB (1.5 equiv) (Scheme 35) [39]. A total of fifteen 3-(bromomethylene)isobenzofuran-1(3*H*)-ones **54** were obtained in moderate to very good yields, starting from alkynyl-substituted substrate **52** bearing electron-rich and -deficient aryl groups. Good results were obtained using the vinyl derivative **52h** (R^1^ = cyclohex-1-enyl), which reacted smoothly to yield the product **54h** at a 67% yield. Equally good outcomes were observed using C*sp*-substituted with alkyl groups (R^1^ = phenylpropanyl, *^n^*Bu and *^t^*Bu), and the respective products **54i**–**k** were obtained at good yields (Scheme 35).

**Scheme 36:**
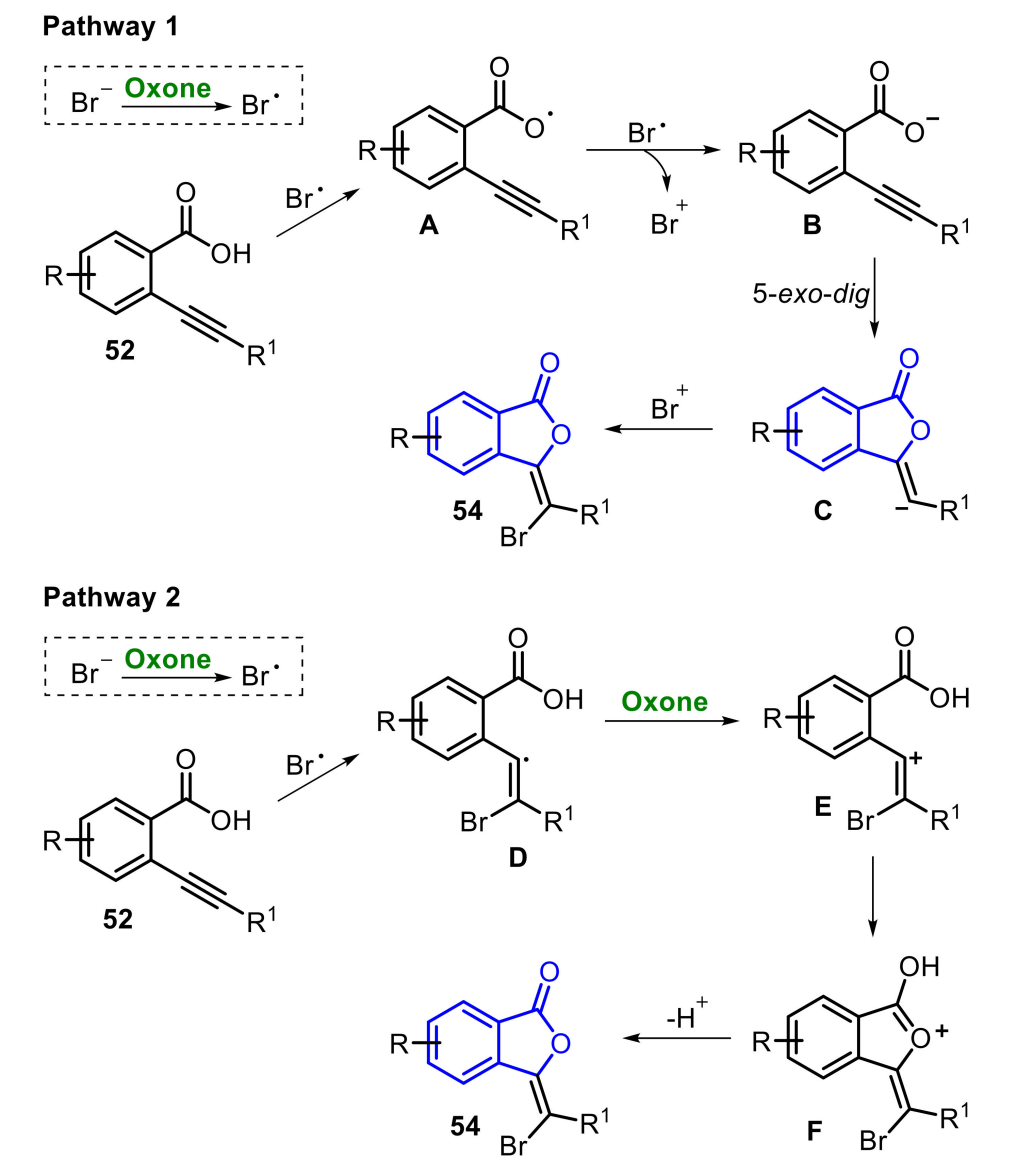
Plausible mechanism of the Oxone/TBAB-promoted radical cyclization of 2-alkynylbenzoic acids **52**.

The proposed mechanism of the formation of **54** from **52** has two possible pathways (Scheme 36). Initially, an Oxone-promoted SET process affords the bromo radical intermediate, which discloses the radical process. Thus, considering the pathway 1, the substrate **52** is initially oxidized by the bromo radical (Br•), releasing HBr and generating the 2-alkynylbenzoic acid radical **A**. Once formed, A is reduced by another equivalent of Br•, to produce the alkynylbenzoic acid anion **B** and a bromonium ion (Br^+^). Following on from this, the intermediate **B** undergoes a 5-*exo*-*dig* cyclization to form the isobenzofuran-1-one anion **C**, which combines with the bromonium ion, to afford the respective product **54**. On the other hand, in the pathway 2, the bromo radical (Br•) firstly adds to the C≡C bond, giving the intermediate **D**, which is oxidized by Oxone, to afford the vinyl cation intermediate **E**. Subsequently, an annulative process drives the reaction toward the intermediate **F**, which is finally converted to the desired product **54** by deprotonation (Scheme 36).

**Scheme 37:**
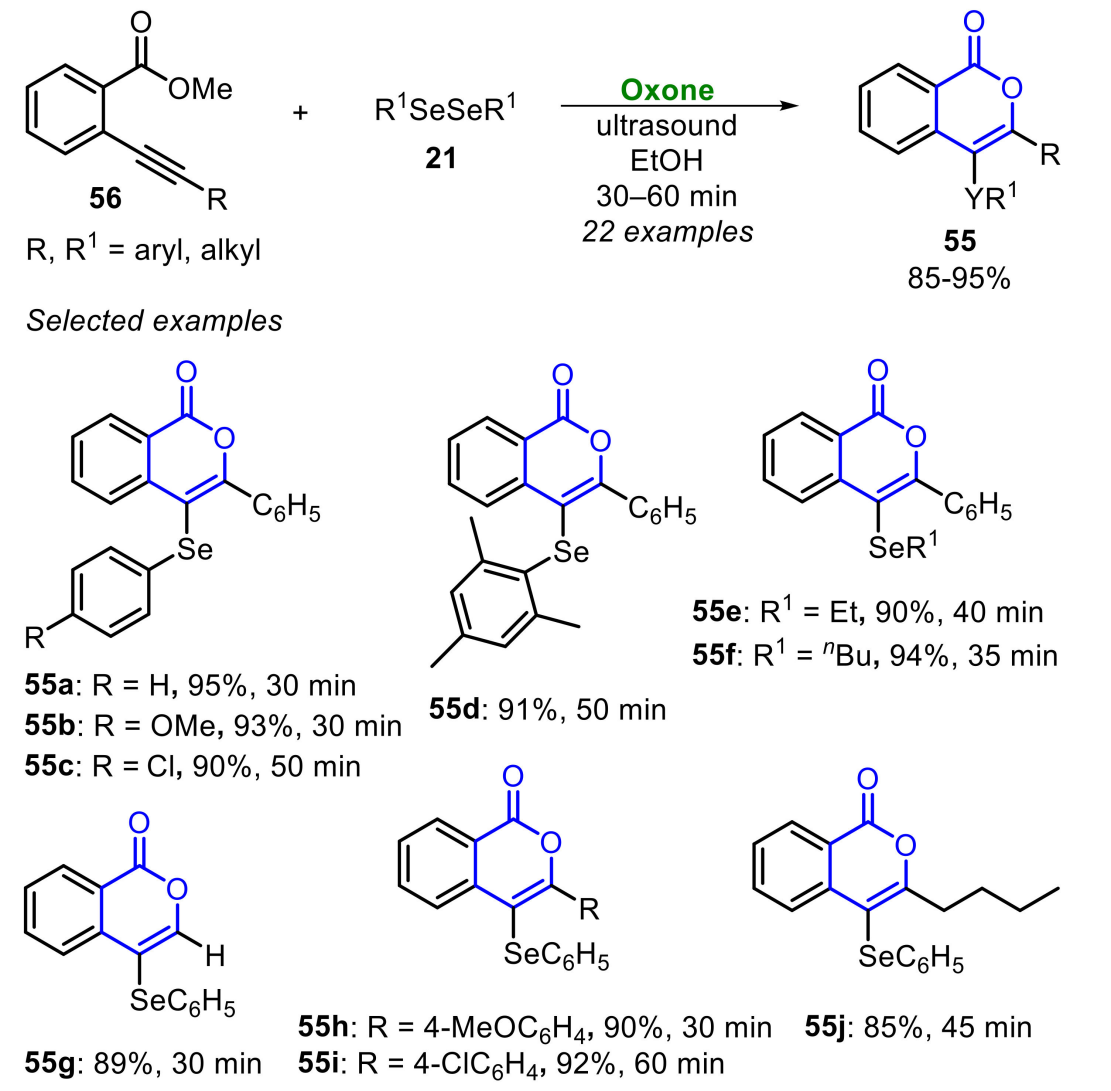
Oxone/diselenide-promoted electrophilic cyclization of 2-alkynylaryl esters **56**.

Very recently, some of us [40] described a simple and general Oxone/diselenide-promoted ultrasound-assisted protocol to prepare 4-chalcogenyl-1*H*-isochromen-1-ones **55**, through a 6-*endo*-*dig* electrophilic cyclization of 2-alkynylaryl esters **56** (Scheme 37) [40]. By using EtOH as solvent, the reaction scope to access Se-containing isochromen-1-ones **55** was investigated using several diorganyl dichalcogenides **21**, **41**, **42** and 2-alkynylaryl esters **56**. Diaryl and dialkyl diselenides **21** were suitable substrates, yielding the products **55a**-**f** in excellent yields, with the reaction time ranging between 30 and 50 min. 2-Alkynylaryl esters **56** bearing a diversity of alkynyl-substituted groups (R groups) reacted smoothly to produce the products **55g**–**j** in very good to excellent yields, in a reaction time ranging from 30 to 60 min. For instance, some examples can be highlighted, including (1) the synthesis of C3-free isochromen-1-ones **55g**, which may be derivatized through a TM-catalyzed cross-coupling strategy, as well as (2) the synthesis of the C3-alkyl derivative **55j**, both in high yields (Scheme 37). 

**Scheme 38:**
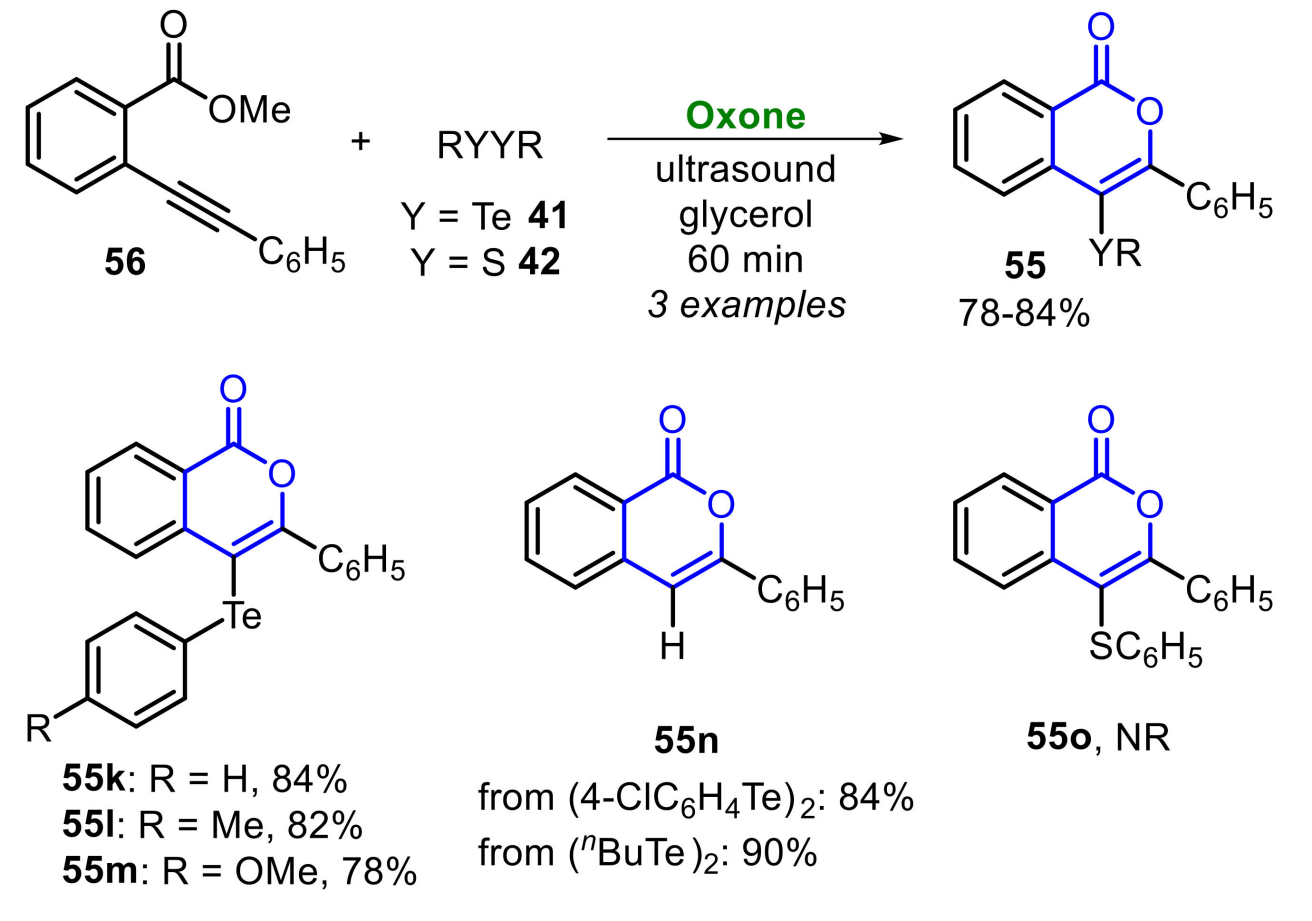
Oxone/ditelluride-promoted electrophilic cyclization of 2-alkynylaryl esters **56**.

The reaction scope was expanded to tellurium-containing isochromen-1-ones **55k**–**n**, employing glycerol as solvent. Neutral and electron-rich aryl ditellurides **41** reacted smoothly to produce the respective products **55k**–**m** in very good yields, under sonication for 1 h. Surprisingly, 4-chlorophenyl ditelluride and dibutyl ditelluride were not able to afford the respective tellurium-containing isochromen-1-ones **55**; the formation of the 3-phenyl-1*H*-isochromen-1-one **55n** was observed in these cases. Moreover, a limitation was also found when diphenyl disulfide **42** was employed as substrate, and the expected sulfur-containing derivative **55o** could not be obtained (Scheme 38).

**Figure 1:**
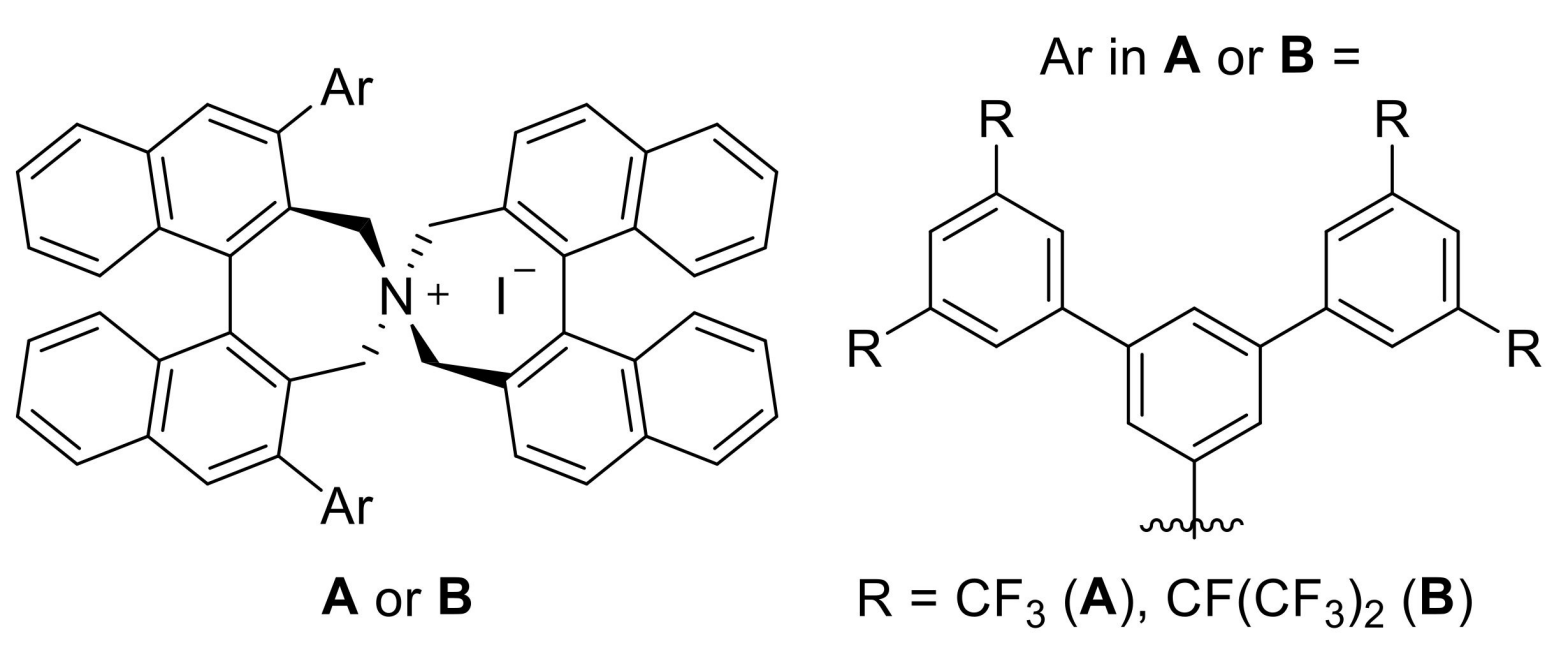
Structures of quaternary ammonium hypoiodite **A** and **B**.

In 2019, Ishihara and co-workers [41] described the Oxone-promoted dearomatization of functionalized arenols in the presence of quaternary ammonium hypoiodite. In this study, the authors proposed the use of two chiral quaternary ammonium hypoiodites (Figure 1), and carried out the oxidation of phenols, 1- and 2-naphthols. 

**Scheme 39:**
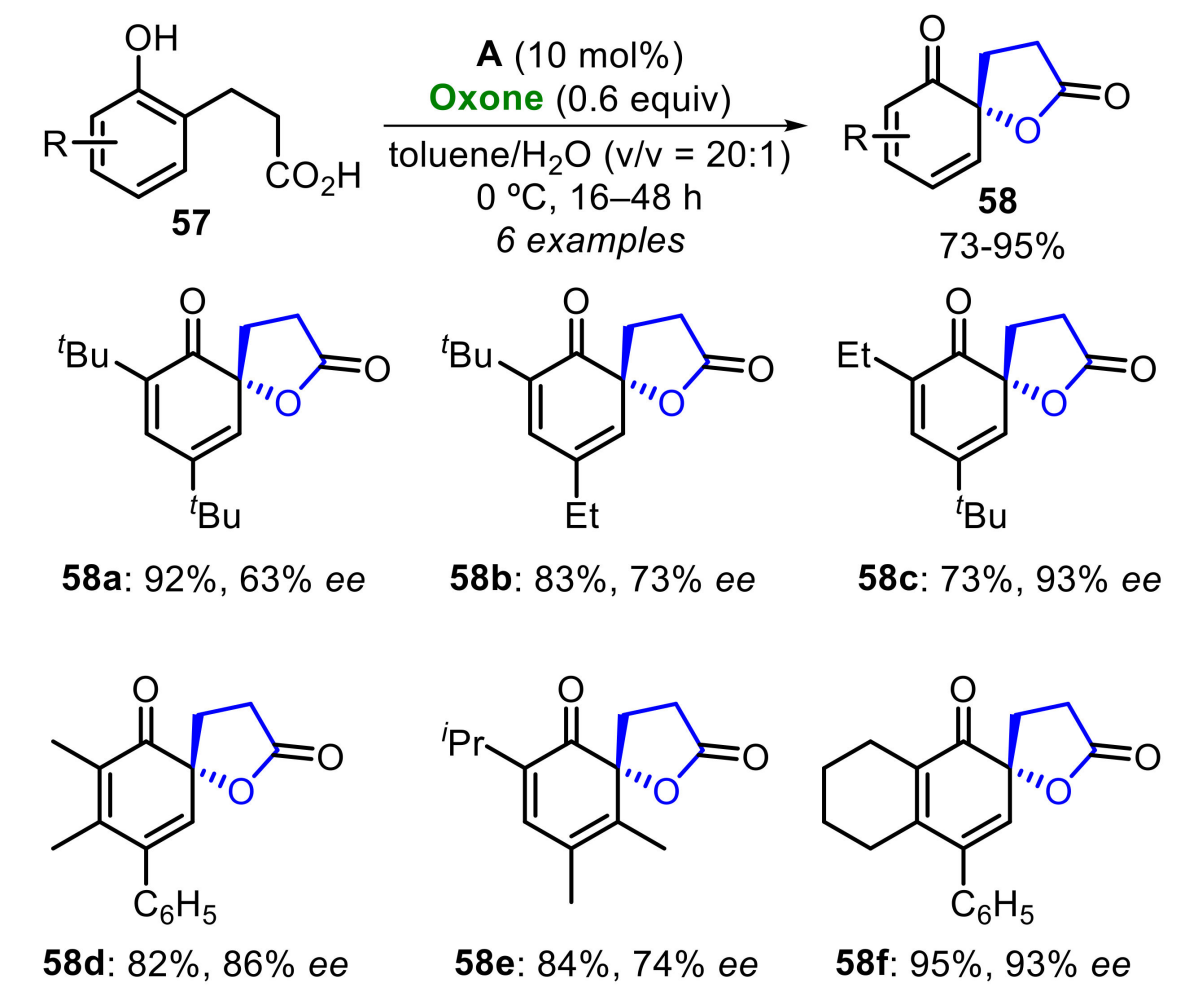
Enantioselective oxidation of phenols **57a**–**f**.

Initially, several phenols substituted with aliphatic groups were used for the enantioselective oxidative spirolactonization using chiral ammonium hypoiodite **A** or **B** (Scheme 39). When using the phenols **57a**–**f**, the corresponding spirolactones **58a**–**f** were obtained at moderate to good yields (73–95%) and with good to high enantioselectivity (73–93% *ee*) after 16–48 h. It was observed that when naphthols with bulky substituents in the *ortho*-position were used, a decrease in the enantioselectivity of the respective compounds **58a**, **58b** and **58e** was observed (Scheme 39). 

**Scheme 40:**
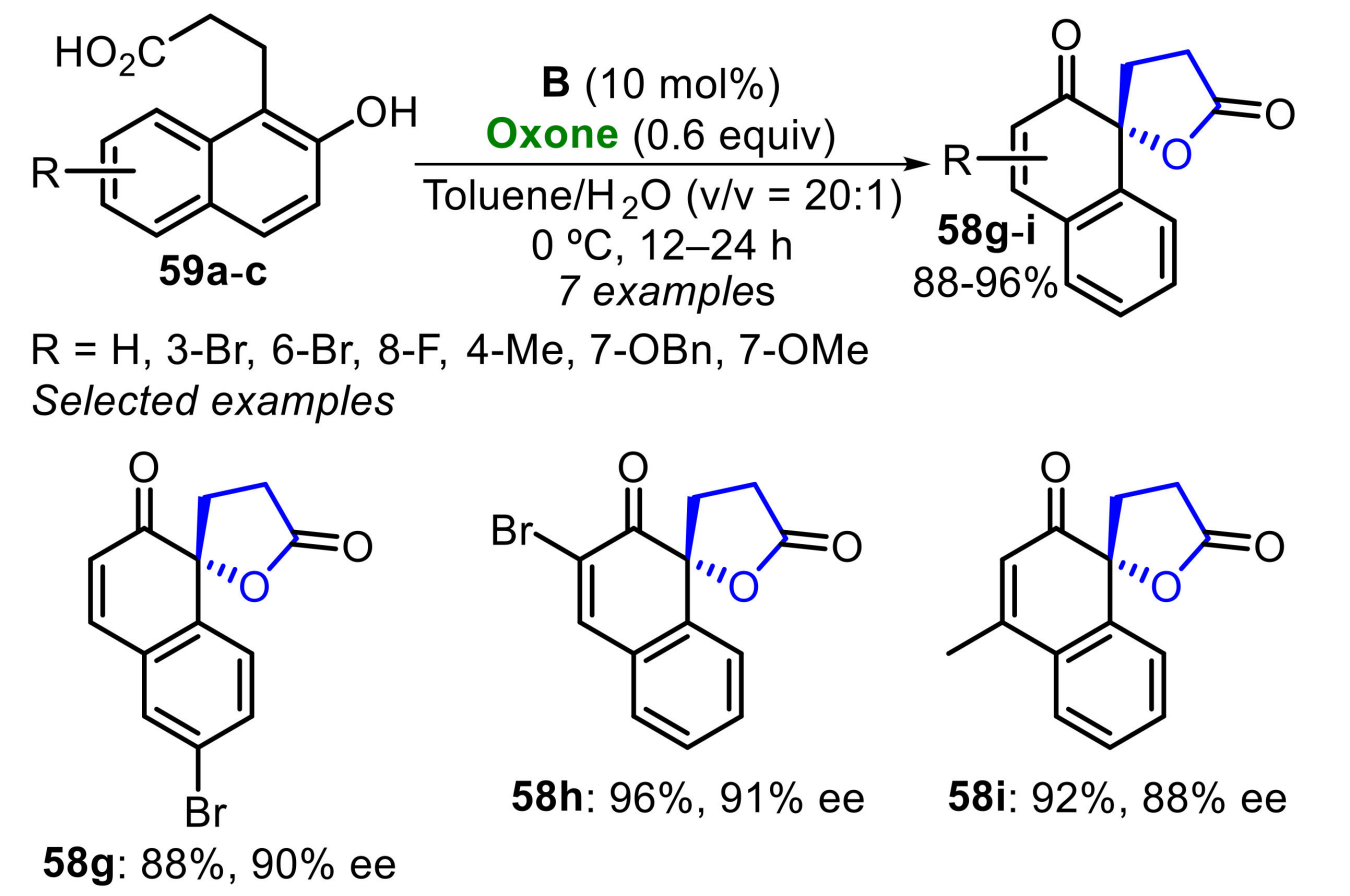
Enantioselective oxidation of 2-naphthols **59a**–**c**.

Concomitantly, under the optimized conditions, several 2-naphthol **59a**–**c** derivatives were oxidized to the corresponding spirolactones **58g**–**i** in very good to excellent yields (88–96%), and with high enantioselectivity (85–91% *ee*), regardless of the electronic nature and position of the substituents (Scheme 40). The best hypoiodite in this reaction was the derivative **B**. 

**Scheme 41:**
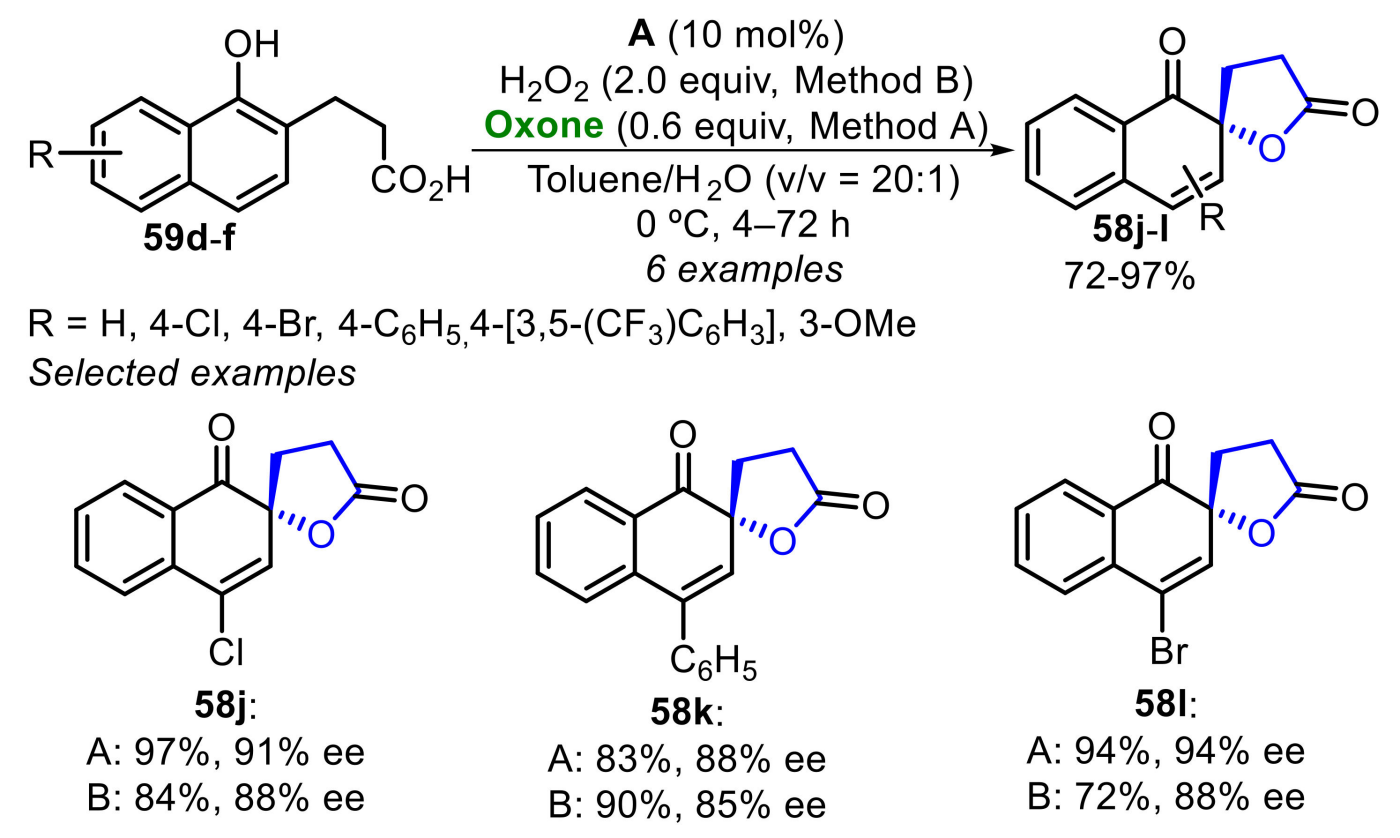
Enantioselective Oxidation of 1-Naphthols **59d–f**.

On the other hand, the oxidation of 1-naphthols **59d–f** was examined using hypoiodite **A**/Oxone catalysis under two conditions: the optimal one described above (method A), and for comparison the reactions were carried out using 2 equiv. of H_2_O_2_ (method B), this condition was tested in the reaction optimization step. Thus, the corresponding spirolactones **58j**–**l** were obtained at higher chemical yields and enantioselectivity, and in shorter reaction times when using method A. These results again demonstrated the good efficiency of hypoiodite/Oxone catalysis (Scheme 41).

## 4. Organochalcogen-Containing Heterocycles

Chalcogenophenes are a valuable class of heterocyclic compounds, which play an important role as building blocks in organic synthesis [42], in the development of new materials [43,44], and in the prospection of new drugs in the pharmaceutical industry [45,46]. Due to these important features, the search for the development of simple and efficient methods to access chalcogenophenes has attracted interest during the last decades. In this context, the use of the system Oxone/dichalcogenide has demonstrated to be a very efficient strategy to access chalcogenophenes through chalcogen-based electrophilic cyclization processes.

**Scheme 42:**
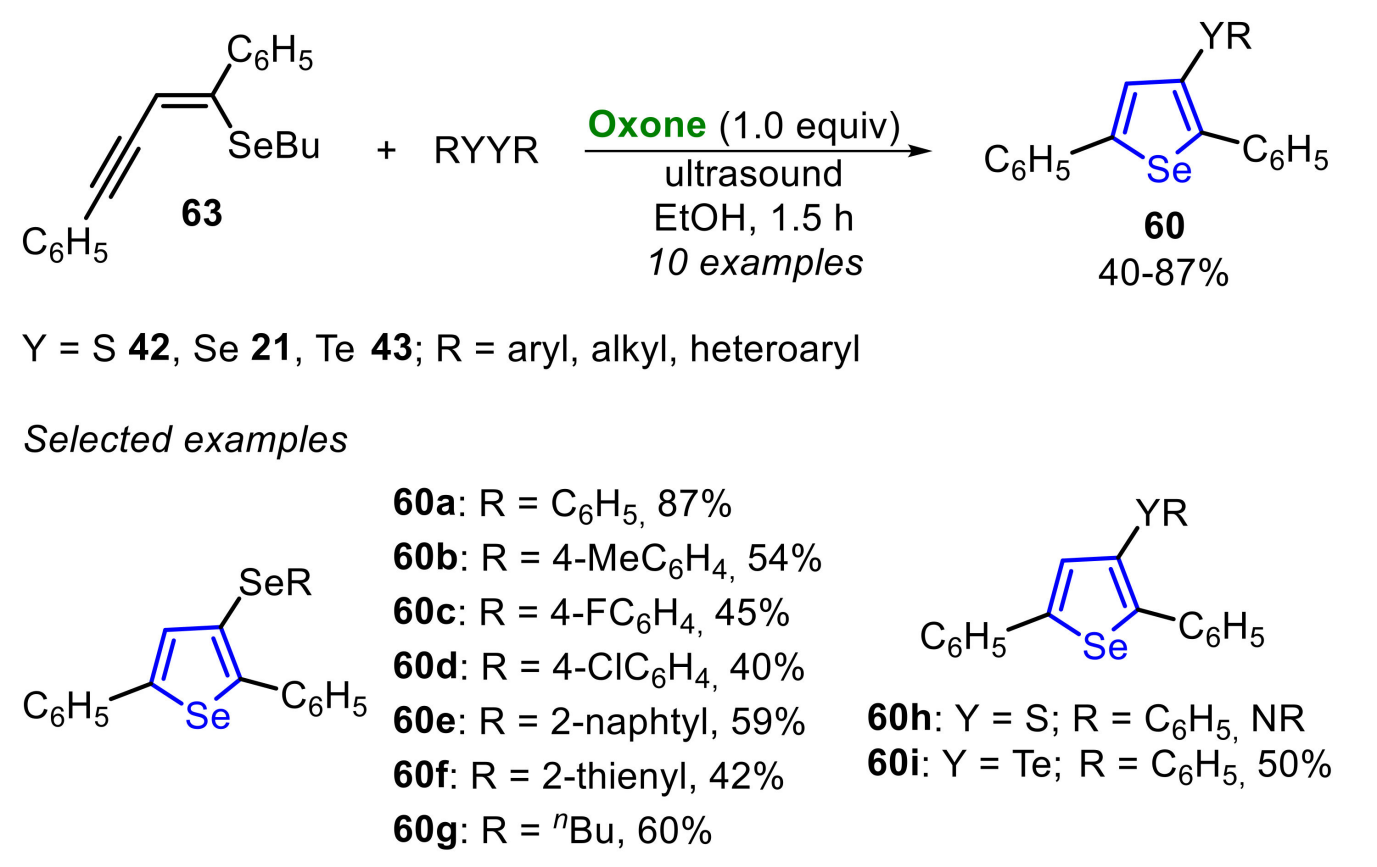
Oxone/dichalcogenide-promoted annulation of (*Z*)-1-butylselen-1-en-3-ynes **63**.

In 2020, some of us reported an Oxone/dichalcogenide-promoted synthesis of 3-selanyl-chalcogenophenes and 3-tellanylchalcogenophenes **60–62**, through the electrophilic cyclization of (*Z*)-chalcogenoenynes **63–65**, under ultrasound irradiation (Scheme 42) [47]. The annulation of (*Z*)-1-butylselen-1-en-3-ynes **63** was conducted in the presence of several diorganyl diselenides **21** and diphenyl ditelluride **41a**, affording the respective 3-arylselanyl- and 3-aryltellanylselenophenes **60** in poor to very good yields. Initially, by employing diphenyl diselenide **21a**, the respective product **60a** was obtained in 87% yield. On the other hand, the reaction was remarkably affected by the presence of substituents attached to the Se-phenyl ring, and the products **60b**-**d** were obtained in just 40–54% yield. Heteroaryl diselenides **21** (R = 2-naphtyl and 2-thienyl) were smoothly submitted to the optimized reaction conditions to afford the corresponding products **60e** and **60f** in 59% and 42% yield, respectively. It is worth to mention that dibutyl diselenide **21** reacted efficiently to produce the selenophene **60g** in 60% yield. Finally, as already observed in other Oxone-promoted oxidative cleavage of the chalcogen–chalcogen bond, diphenyl disulfide **42a** failed to access the expected product **60h**, whereas diphenyl ditelluride **41a** reacted satisfactorily to be converted to the tellurium-functionalized selenophene **60i** at a 50% yield (Scheme 42).

**Scheme 43:**
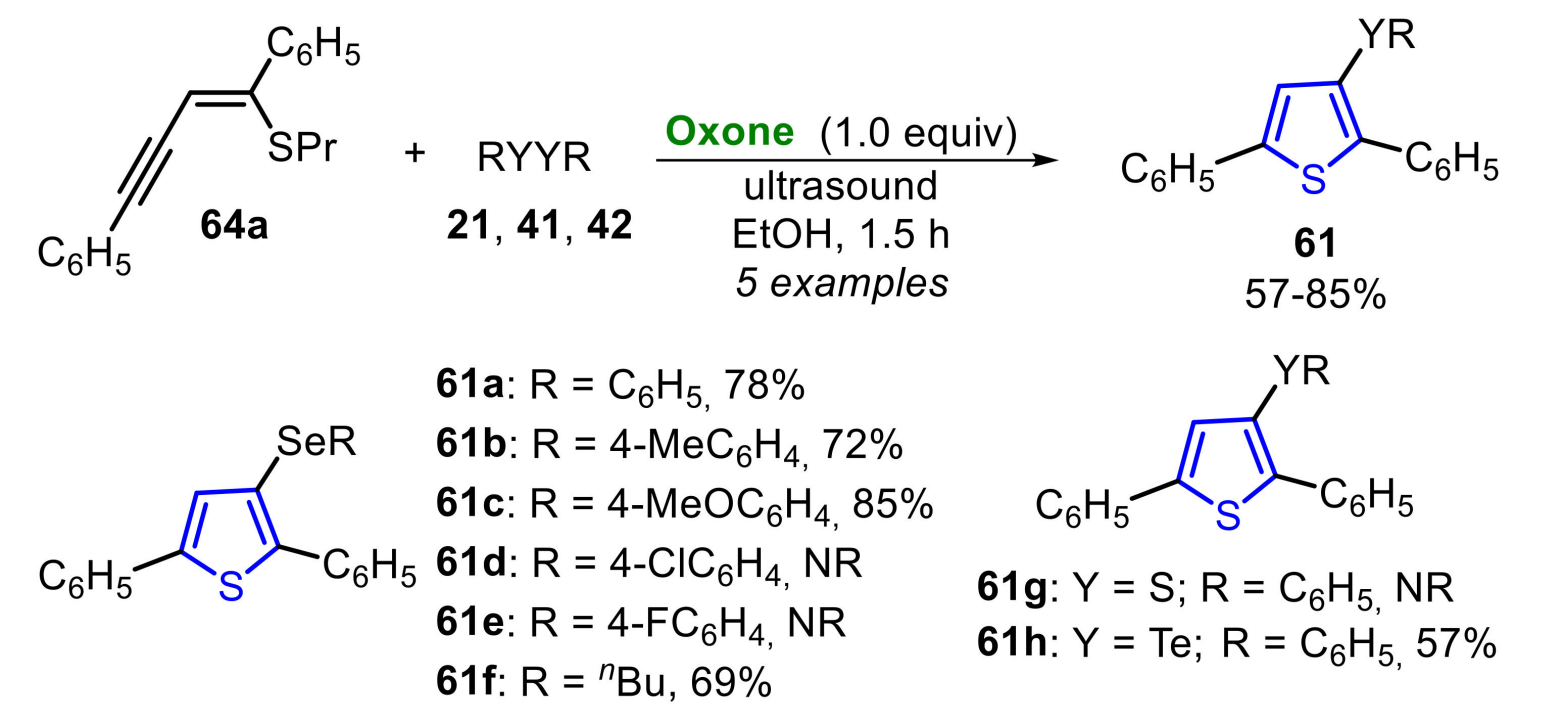
Oxone/dichalcogenide-promoted annulation of (*Z*)-(1,4-diphenylbut-1-en-3-yn-1-yl)(propyl)sulfane **64a**.

The reaction scope was expanded to (*Z*)-(1,4-diphenylbut-1-en-3-yn-1-yl)(propyl)sulfane **64a** at the same optimal conditions, aiming to access a library of chalcogen-functionalized thiophenes **61** (Scheme 43). The authors observed that neutral and electron-rich diaryl diselenides **21** were suitable electrophile precursors, affording the respective 3-selanylthiophenes **61a–c** in 72% to 85% yield. However, the presence of halogen at the *para*-position of the Se-phenyl ring of the diaryl diselenide (R = 4-FC_6_H_4_ and 4-ClC_6_H_4_), prevented the formation of the respective thiophenes **61d** and **61e**. Dibutyl diselenide **21f** reacted smoothly to produce 3-(butylselanyl)-2,5-diphenylthiophene **61f** at a 69% yield. Additionally, in this case, diphenyl disulfide **42a** did not react with enyne **64a** to produce 3-sulfanylthiophene **61g**, whereas diphenyl ditelluride **41a** was efficient in the synthesis of 3-(phenyltellanyl)-2,5-diphenylthiophene **61h**, obtained at a 57% yield (Scheme 43).

**Scheme 44:**
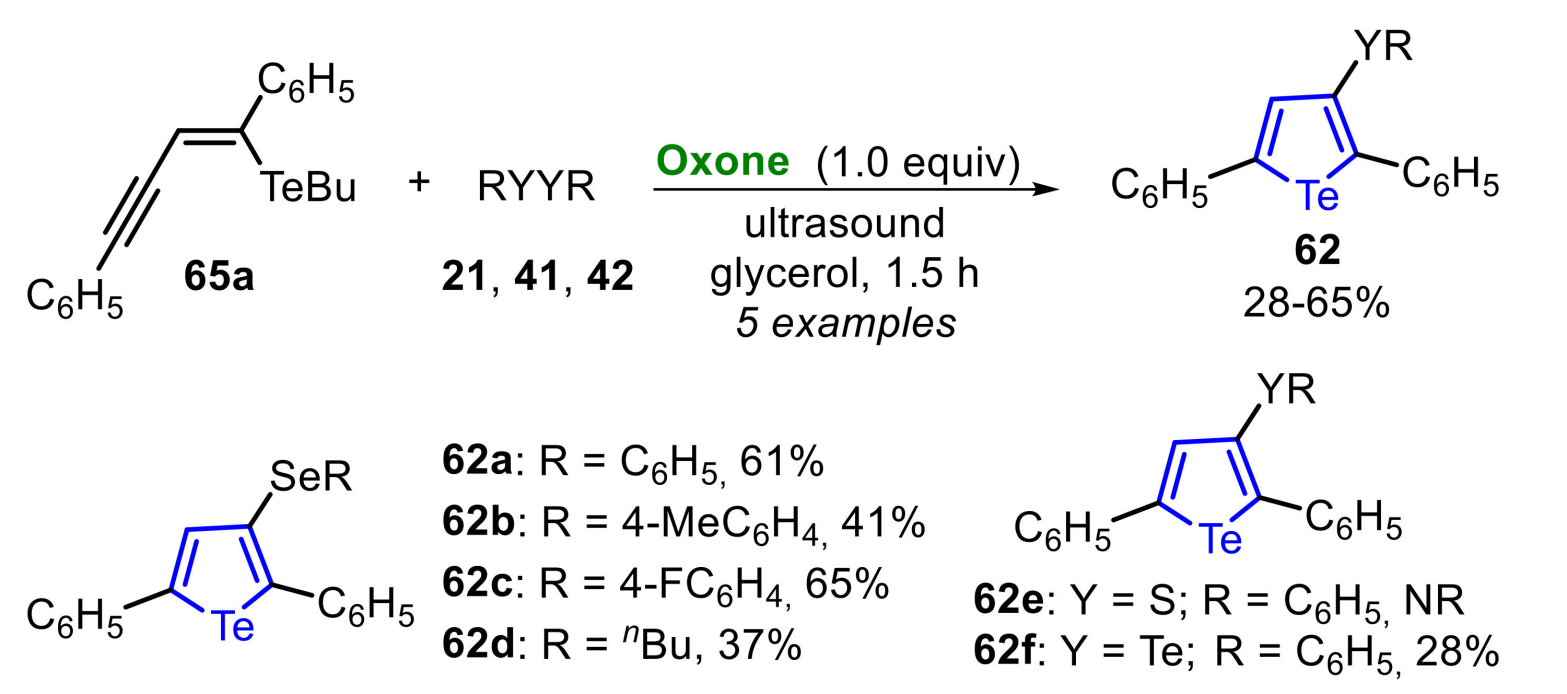
Oxone/dichalcogenide-promoted annulation of (*Z*)-butyl(1,4-diphenylbut-1-en-3-yn-1-yl)tellane **65a**.

Additionally, the protocol was expanded for the synthesis of tellurophenes **62**, using (*Z*)-butyl(1,4-diphenylbut-1-en-3-yn-1-yl)tellane **65a** in the reaction with Oxone/dichalcogenide, in the presence of glycerol as solvent. In this case, the reaction temperature could be increased to up 100 °C under ultrasound, which was necessary to allow the cyclization (Scheme 44). Under this condition, electron-rich and electron-deficient diaryl diselenides **21** reacted adequately to produce the products **62a**–**c** at moderate yields, whereas dibutyl diselenide **21f** afforded the tellurophene **62d** in 37% yield. As previously observed, the protocol was not suitable to diphenyl disulfide **42a**, whereas 3-(phenyltellanyl)-2,5-diphenyltellurophene **62f** was obtained in only 28% by the reaction between **65a** and diphenyl ditelluride **41a** under the optimal conditions (Scheme 44).

**Scheme 45:**
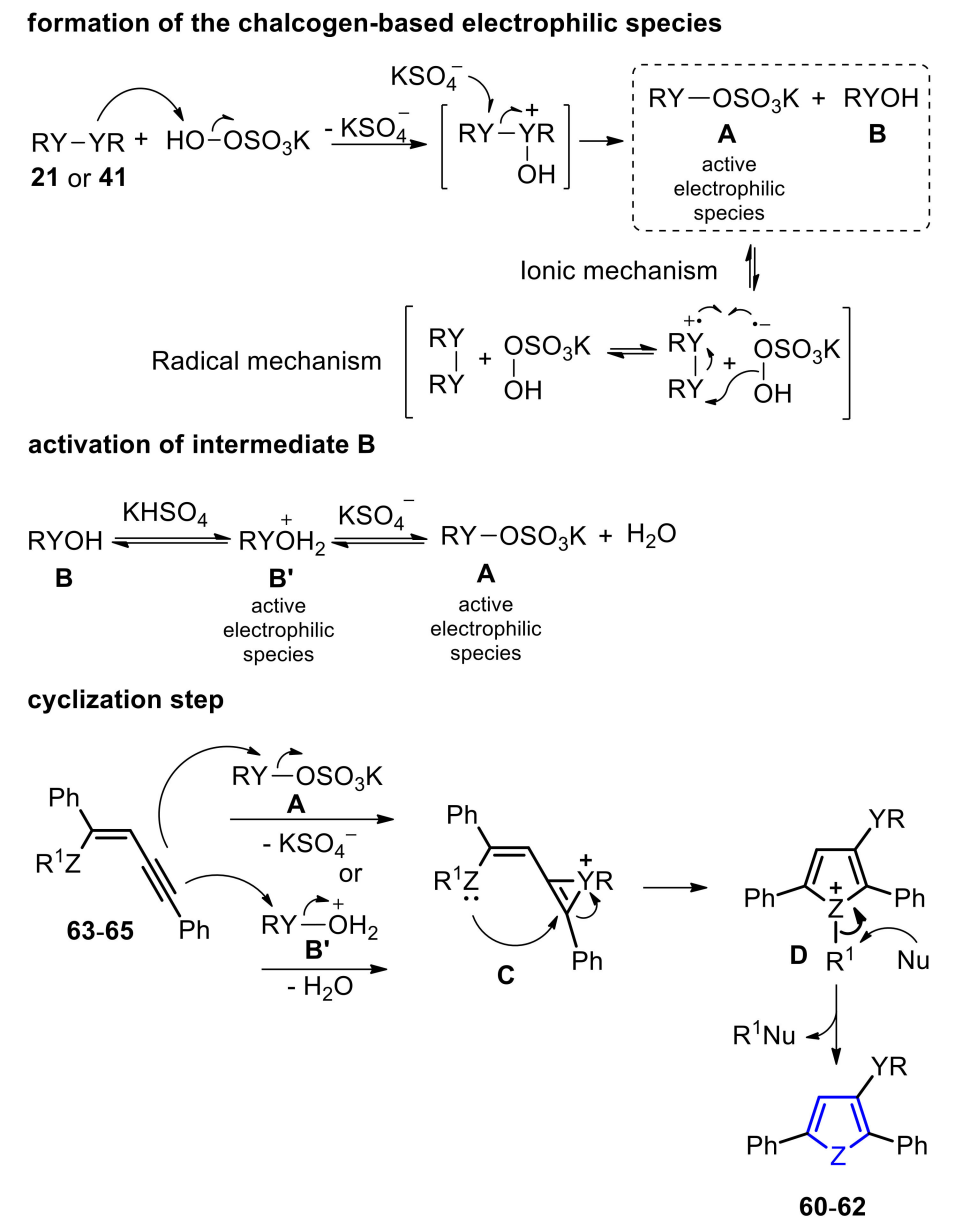
Reaction mechanism for the Oxone/dichalcogenide-promoted cyclization of chalcogenoenynes.

The proposed reaction mechanism for the electrophilic cyclizations follows an ionic and a radical step simultaneously (Scheme 45). Initially, the reactive intermediates **A** and **B** are obtained via anionic or radical pathways, by the reaction between the diorganyl dichalcogenide **21** or **41** and potassium peroxymonosulfate (KHSO_5_, the active component of Oxone). Once formed, the intermediate **B** can be protonated to afford the strongest electrophile **B′**. In the presence of chalcogenoenynes **63**, **64** or **65**, the intermediates **A** and **B′** are converted to the chalcogenironium intermediate **C**, that undergoes an intramolecular annulation to form the intermediate **D**. Finally, an alkyl group displacement, promoted by a nucleophilic species from the reaction medium, yields the desired chalcogenophenes **60–63** (Scheme 45).

**Scheme 46:**
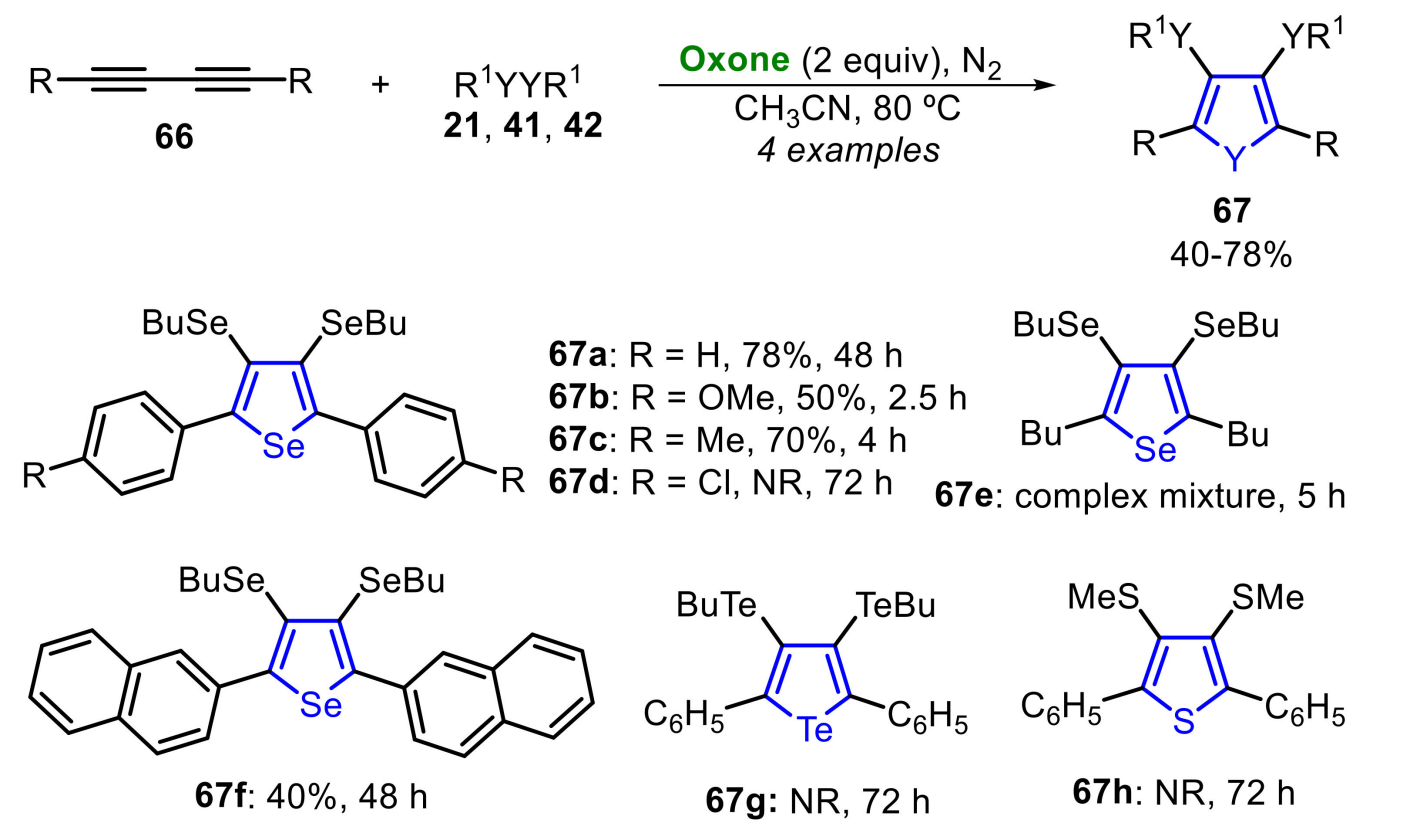
Oxone/dibutyl diselenide-promoted synthesis of thiophenes **67**.

In 2021, some of us reported an Oxone/dibutyl diselenide-promoted 5-*endo*-*dig* electrophilic cyclization of 1,3-diynes **66**, to access selectively 3,4-bis(butylselanyl)selenophenes **67** and 4-alkoxyselenophenes **68** (Scheme 46) [48]. The selectivity in the formation of the products **67** and **68** was achieved by controlling the solvent (MeCN or ROH) and the dibutyl diselenide **21f** amount (2 equiv or 1.5 equiv). Thus, by employing 2 equiv of dibutyl diselenide **21f** in the presence of MeCN as solvent, 3,4-bis(butylselanyl)selenophenes **67** were prepared in moderate to good yields, after being stirred at 80 °C. Neutral and electron-rich 1,3-diynes **66** (R = H, OMe and Me) reacted adequately to yield the products **64a**, **67b** and **67c** in 78%, 50% and 70% yield, respectively. On the other hand, the electron-deficient 1,4-bis(4-chlorophenyl)buta-1,3-diyne (**66d**, R = 4-ClC_6_H_4_) could not be converted to the expected product **67d**, even after 72 h of reaction. By employing dodeca-5,7-diyne (**66e**, R = *^n^*Bu) as substrate, a complex mixture of products was obtained. Interestingly, 1,4-di(naphthalen-2-yl)buta-1,3-diyne **66f** allowed the formation of the selenophene **67f** in 40% yield, after 48 h. Finally, dibutyl ditelluride **41d** and dimethyl disulfide **42b** were submitted to the optimized reaction conditions; however, both were not able to produce the expected products **67g** and **67h**, even after 72 h of reaction (Scheme 46).

**Scheme 47:**
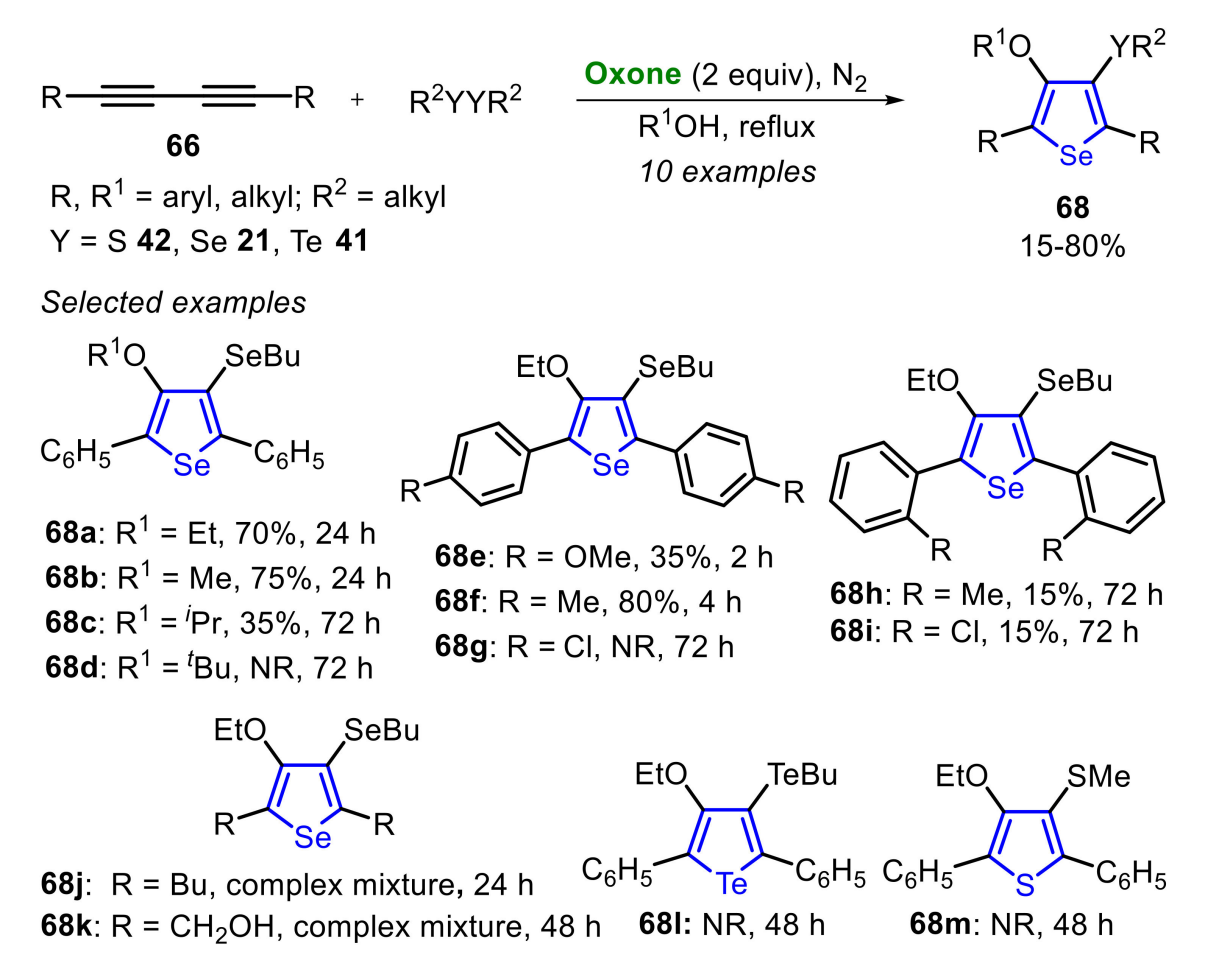
Oxone/dibutyl diselenide-promoted synthesis of thiophenes **68**.

On the other hand, by employing 1.5 equiv of dibutyl diselenide **21f** and aliphatic alcohols as solvent/nucleophiles under reflux, the reaction was driven selectively to 3-(butylselanyl)-4-alkoxyselenophenes **68**, that were afforded in low to very good yields (Scheme 47). Initially, several alcohols (EtOH, MeOH, *^i^*PrOH and *^t^*BuOH) were employed as solvent in the presence of the 1,3-diyne **66a** and dibutyl diselenide **21f**. Among them, EtOH and MeOH were able to conduct the reaction adequately, affording the products **68a** and **68b** at a 70% and 75% yield, respectively. On the other hand, in the presence of the sterically hindered *^i^*PrOH and *^t^*BuOH, a remarkable loss of efficiency was faced, with product **65c** (R^1^ = *^i^*Pr) being obtained at a 35% yield, whereas **68d** (R^1^ = *^t^*Bu) could not be obtained. Different symmetrical 1,3-diynes **66** where employed as substrate in the reaction with dibutyl diselenide **21f** in the presence of EtOH, affording the products **68e**–**i**. For instance, the electron-rich methoxy-derivative diyne (R = 4-MeOC_6_H_4_) afforded **68e** in just 35% yield, whereas the *p*-tolyl analogue (R = 4-MeC_6_H_4_) was a more suitable substrate, affording **68f** in 80% yield. On the other hand, no product **68g** was observed when the *para*-chlorophenyl ring was attached to the 1,3-diyne (R = 4-ClC_6_H_4_). When the sterically hindered *ortho*-substituted diynes **66e** (R = 2-MeC_6_H_4_) and **66f** (R = 2-ClC_6_H_4_) were used, the respective 4-ethoxyselenophenes **68h** and **68i** were obtained both at a 15% yield after 72 h. Alkyl-substituted 1,3-diyne (**66e**, R = *^n^*Bu) and hexa-2,4-diyne-1,6-diol (**66h**, R = CH_2_OH) afforded a complex mixture of products, not yielding the respective selenophenes **68j** and **68k**. As observed above, in the reactions in acetonitrile (Scheme 42), dibutyl telluride **41d** and dimethyl disulfide **42b** were not suitable dichalcogenides in this reaction, and the formation of products **68l** and **68m** was not observed, even after 48 h (Scheme 47).

**Scheme 48:**
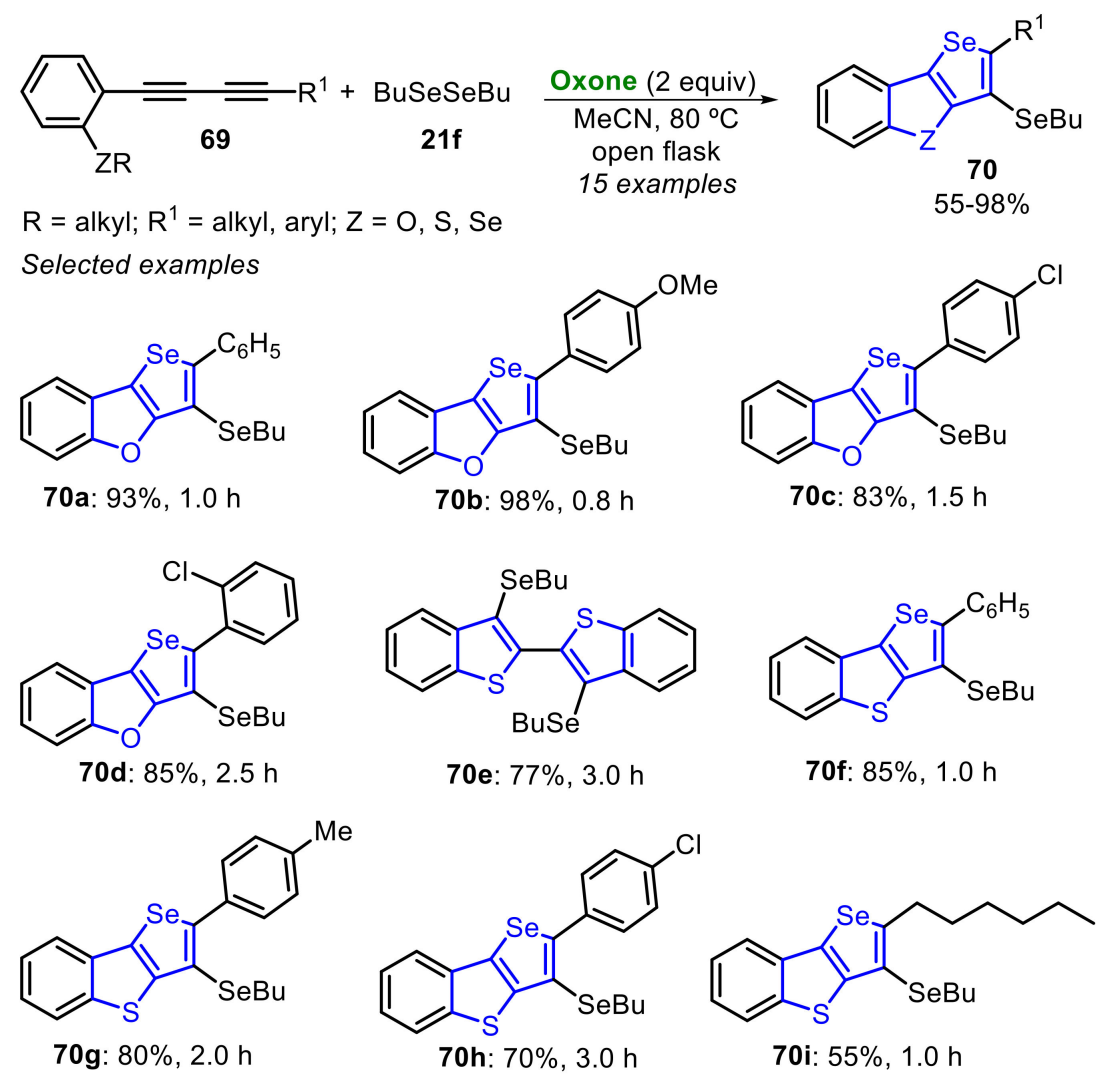
Oxone/dibutyl diselenide-promoted intramolecular cyclization of *ortho*-1,3-diynyl phenyl chalcogenide **69**.

The same strategy was used by some of us to access selectively selenophene-fused benzo[*b*]chalcogenophenes **70**, through the intramolecular cyclization of *ortho*-1,3-diynyl phenyl chalcogenides **69** (Scheme 48) [49]. The optimal conditions were efficiently applied to substituted 1,3-diynes **69** bearing electron-neutral and electron-rich aryl groups, to produce the products **70a**–**d** at excellent yields. Good results were obtained even in the presence of a chlorine a the *ortho*-position, giving the product **70d** was obtained in 85% yield. Interesting, symmetric *ortho*-thiosubstituted 1,3-diyne **69e** was a suitable substrate, being satisfactorily converted to 3,3′-bis(butylselanyl)-2,2′-dibenzo[*b*]thiophene **70e** in 77% yield, after 3 h. Additionally, unsymmetric [2-(phenylbuta-1,3-diin-1-yl)phenyl](propyl)sulfide **69f** reacted smoothly to produce the respective selenophene **70f** in 85% yield, after 1 h. The electron-rich substrate **69g** (R^1^ = 4-MeC_6_H_4_) was more reactive in comparison with the electron-deficient one **69h** (R^1^ = 4-ClC_6_H_4_), and the respective products **70g** and **70h** were obtained at a 80% and 70% yield, after 2 and 3 h, respectively (Scheme 48).

**Scheme 49:**
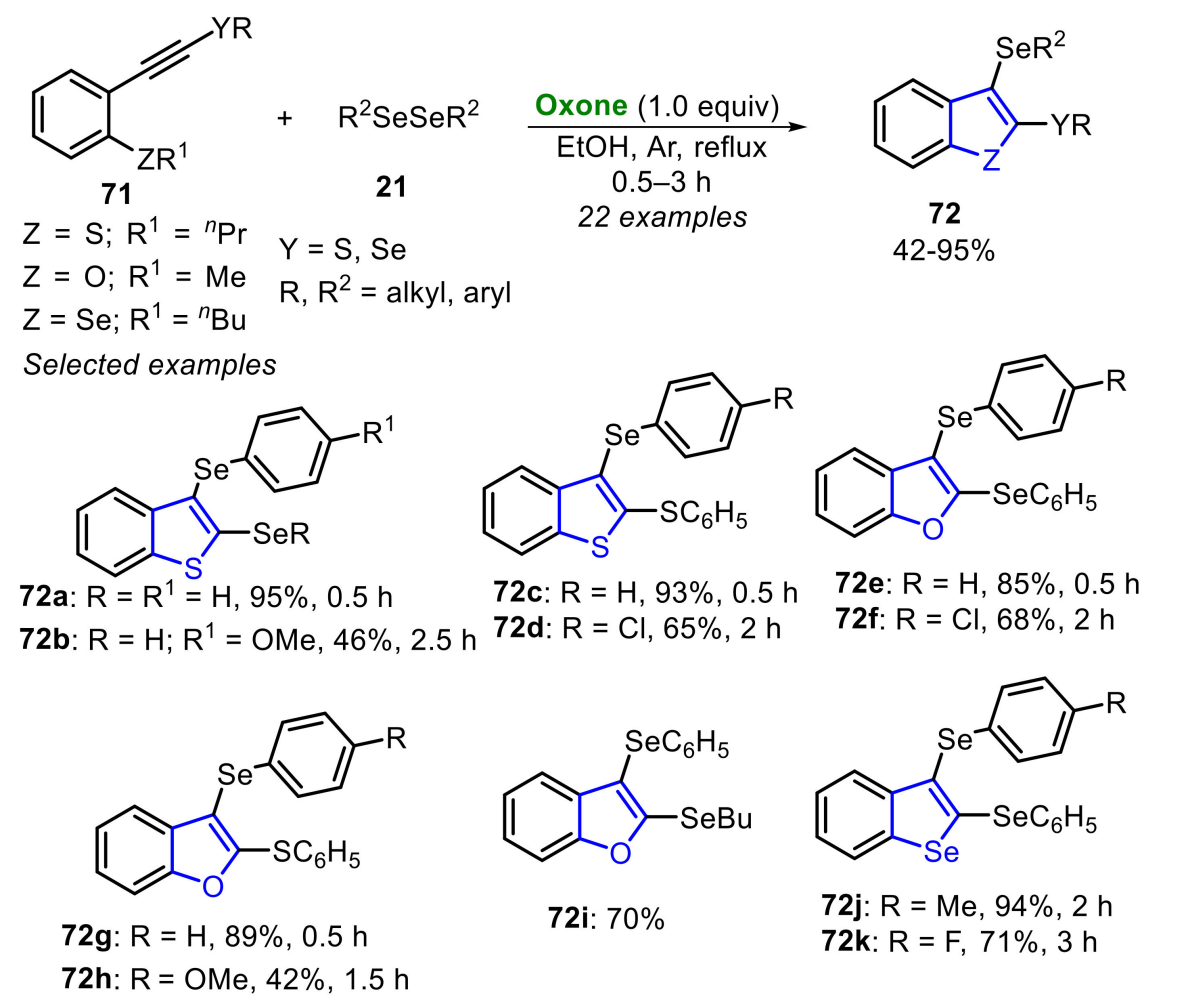
Oxone/diselenide-promoted electrophilic cyclization of 2-functionalized chalcogenoalkynes **71**.

In 2019, some of us reported the Oxone/diselenide-promoted electrophilic cyclization of 2-functionalized chalcogenoalkynes **71**, allowing the access to benzo[*b*]chalcogenophenes **72**, in moderate to excellent yields (Scheme 49) [50]. It is important to highlight the high substrate tolerance presented by the method, allowing the use of alkynes with different alkyl, or electron-rich and electron-deficient aryl substituents. 

## 5. Miscellaneous Cyclizations

Besides the several important transformations discussed above, many other Oxone-promoted cyclization processes have been described during the last few years, to prepare a wide variety of compounds.

**Scheme 50:**
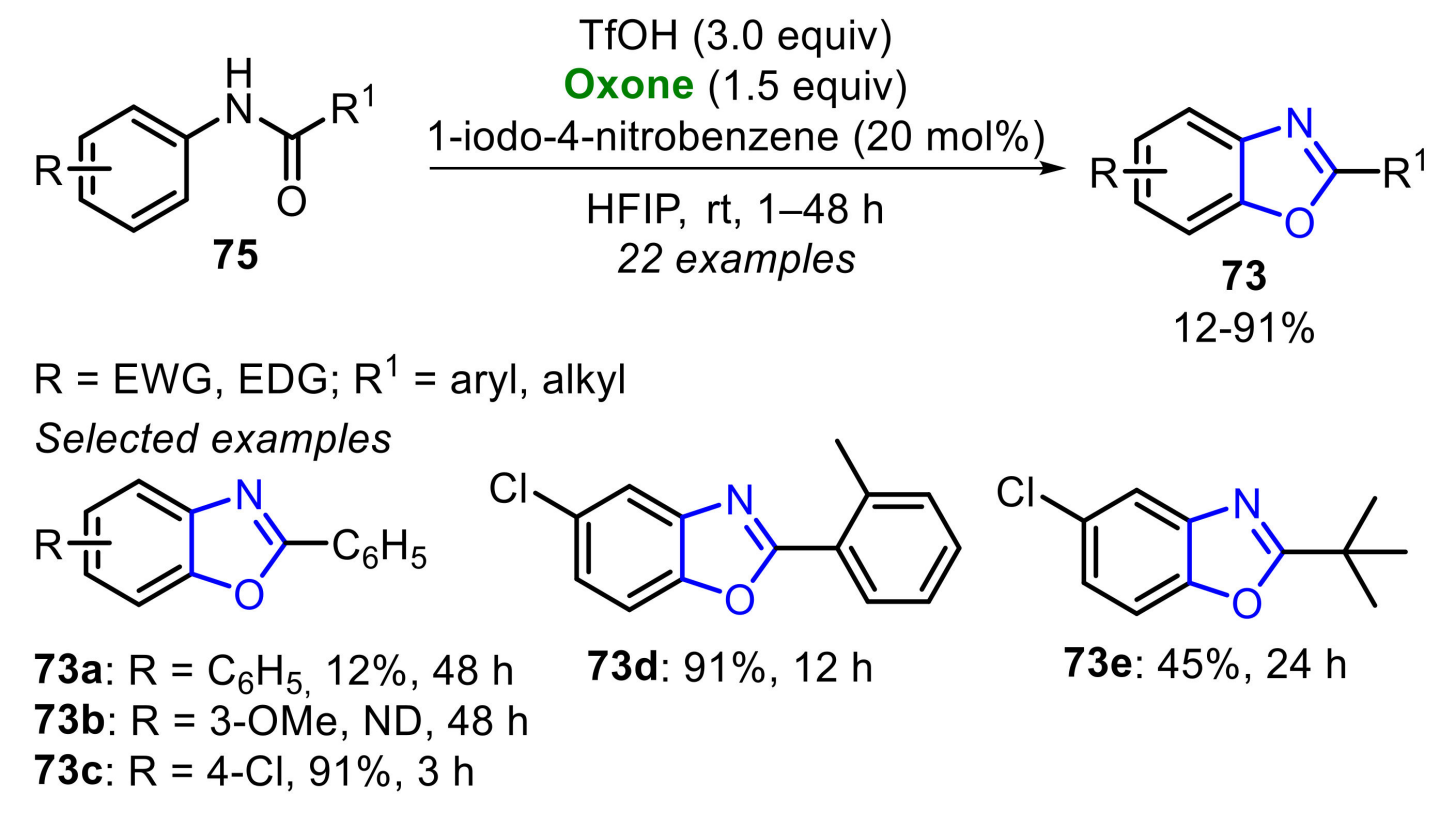
Oxone-mediated synthesis of 2-arylbenzoxazoles **73**.

In 2014, Punniyamurthy and co-workers described the use of Oxone as a green oxidant in the organocatalytic synthesis of 2-arylbenzoxazoles **73** and 2-arylbenzothiazoles **74** (Scheme 50) [51]. In the presence of 1-iodo-4-nitrobenzene (20 mol%) as catalyst, Oxone (1.5 equiv), TfOH (3.0 equiv) and hexafluoro-2-propanol (HFIP, 2.5 mL) as solvent, *N*-*4*-tolylbenzamides **75** were converted to benzoxazoles **73** in poor to excellent yields, under room temperature.

**Scheme 51:**
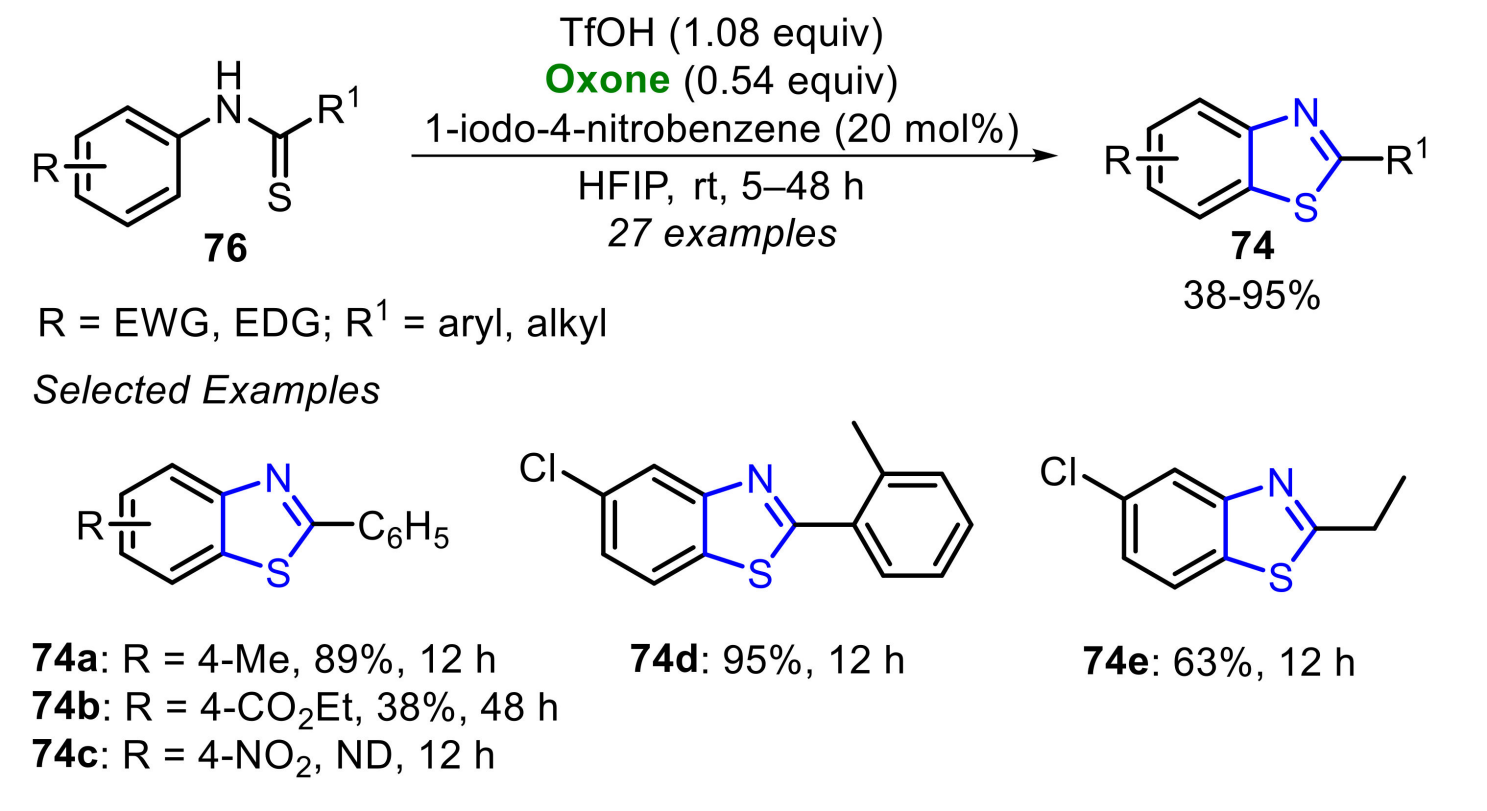
Oxone-mediated synthesis of 2-arylbenzothiazoles **74**.

In parallel, several electron-rich and electron-deficient *N*-phenylthiobenzamides **76** were employed as substrate to access 2-arylbenzothiazoles **74**, under similar conditions, at room temperature (Scheme 51). The protocol showed an excellent substrate tolerance, allowing the use of different *N*-phenylthiobenzamides **76** to prepare a total of twenty-seven substituted benzothiazoles **74** in poor to excellent yields.

**Scheme 52:**
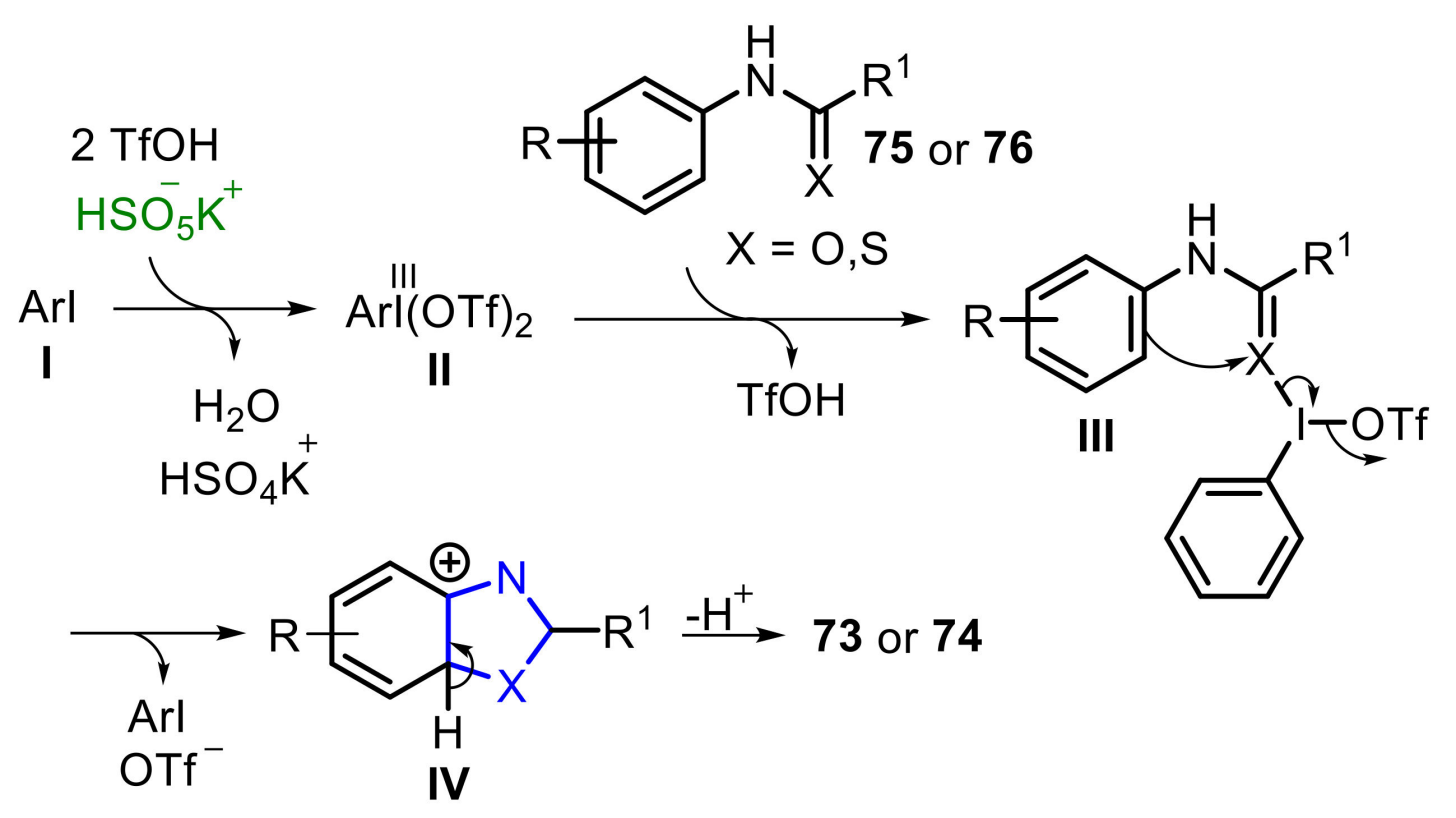
Reaction mechanism for the synthesis of **73** and **74**.

The proposed reaction mechanism starts by the generation of the hypervalent iodine(III) species **II**, by reaction of aryl iodide **I**, TfOH and Oxone (Scheme 52). The species **II** is able to catalyze the oxidative annulation of substrates **75** or **76** to produce the intermediate **III**, that can be stabilized by HFIP. The intramolecular cyclization of **III** affords the cationic intermediate **IV**, releasing iodobenzene **I** to the reaction medium, which is eventually converted to **II** to restart the process. Finally, the intermediate **IV** is easily converted to the aromatic products **73** and **74** by deprotonation.

**Scheme 53:**
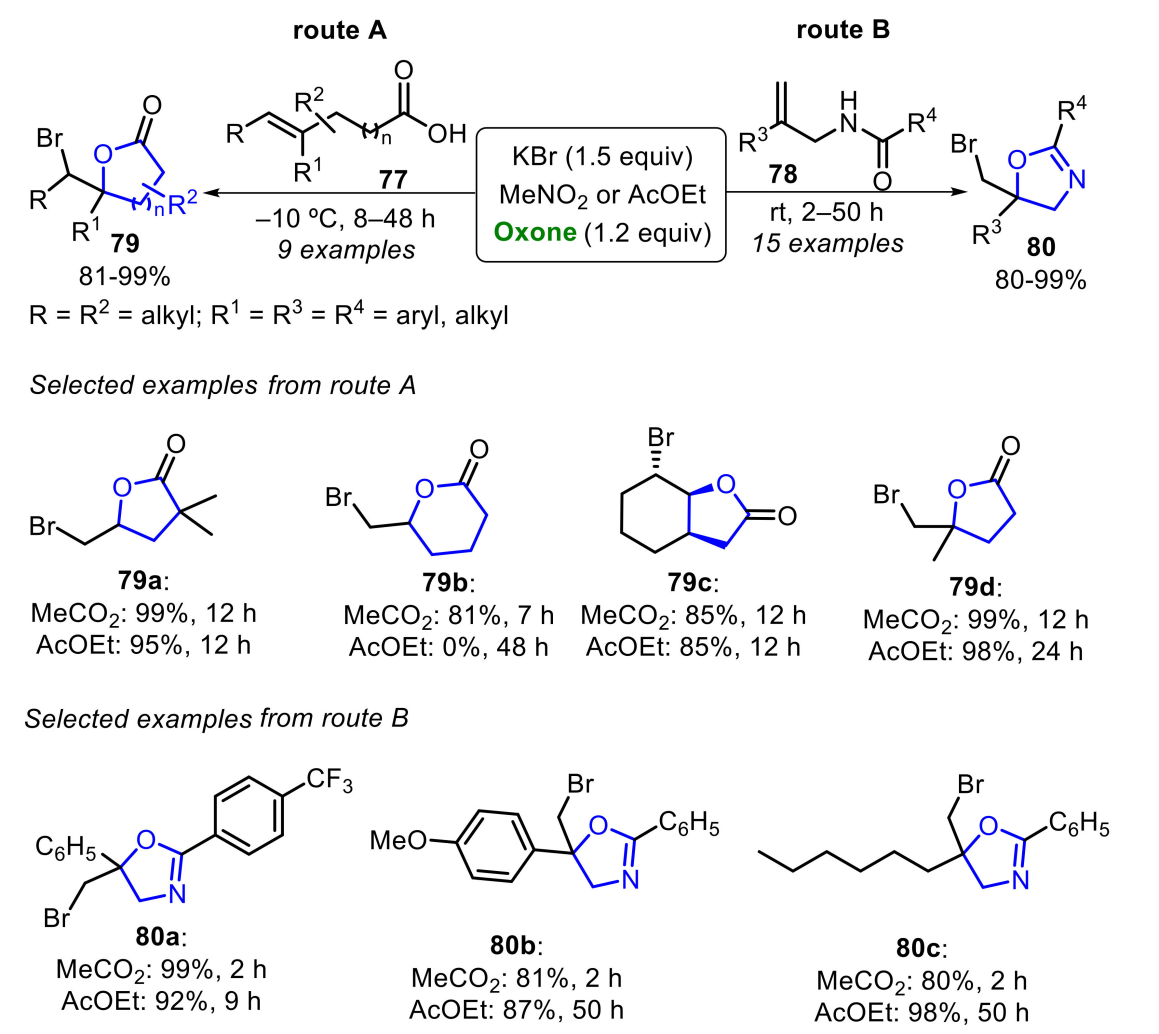
Oxone/KBr-promoted bromo-cyclization of alkenyl carboxylic acids **77** (**A**) and *N*-allyl amides **78** (**B**).

Togo and co-workers described, in 2015, the Oxone/KBr-promoted bromo-lactonization of alkenyl carboxylic acids **77** and bromo-cyclization of *N*-allyl amides **78** to provide dihydrofurans **79** and dihydrooxazoles **80**, respectively (Scheme 53) [52]. The reaction optimization study revealed that MeNO_2_ and AcOEt, acting as solvent, remarkably increased the bromo-lactonization diastereoselectivity. In the presence of MeNO_2_, several substrates *α*,*α*-disubstituted **77** (*gem*- and *vic*-disubstituted alkenes) were converted to the corresponding products **79** in very good to excellent yields (Scheme 53, route A). Additionally, the six-membered ring cyclization proceeded efficiently to produce δ-lactone **79b** at a 81% yield. The bromo-lactonization in AcOEt presented similar behavior, except to access the six-membered ring derivative **79b**, in which no product was observed. Subsequently, the bromo-lactonization of *α*-substituted *N*-allyl amides **78** was investigated, and both MeNO_2_ and AcOEt, were able to produce the desired products **80** at very good to excellent yields (Scheme 53route B).

**Scheme 54:**
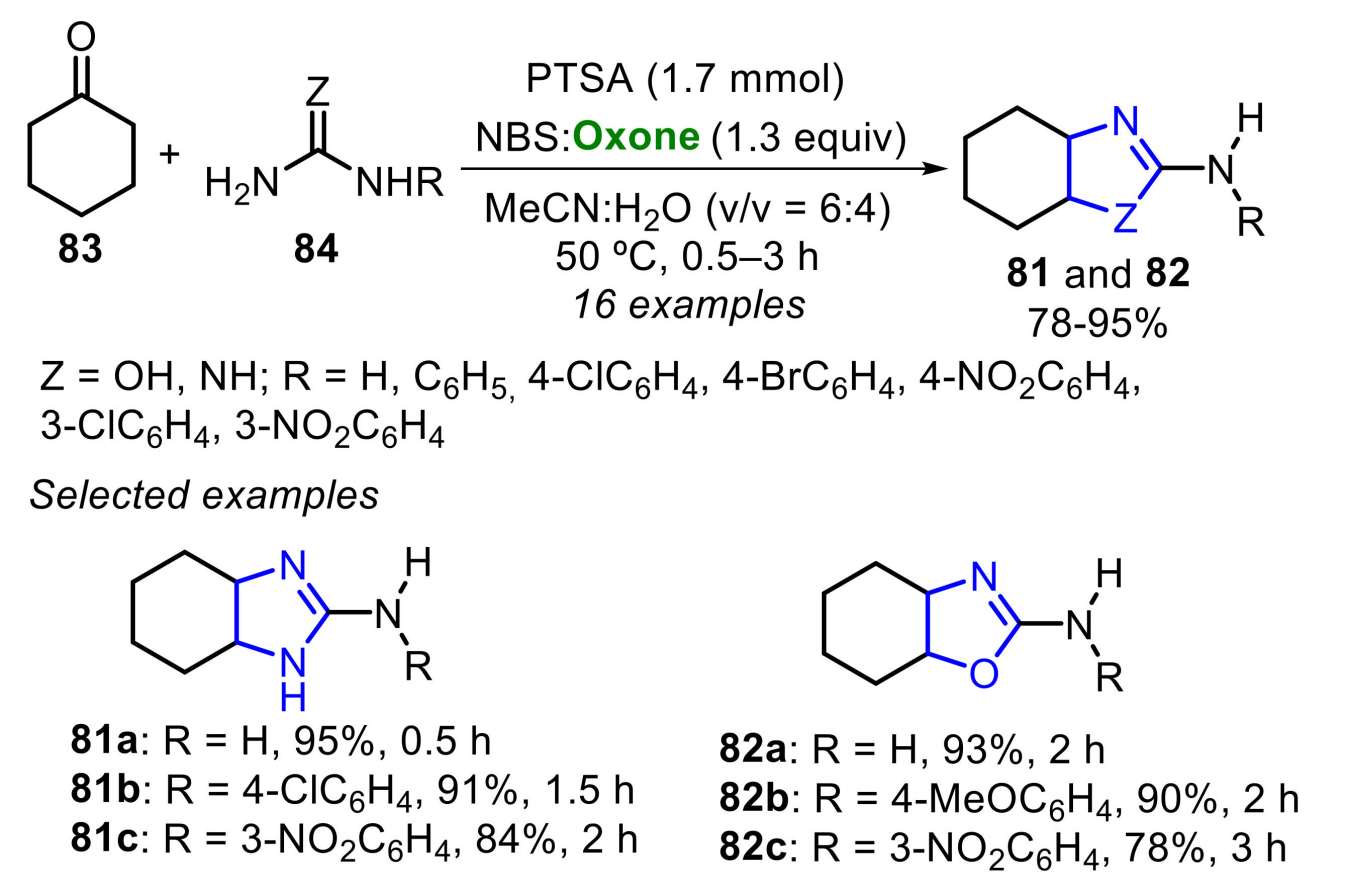
Oxone/NBS-promoted synthesis of 2-aminobenzimidazoles **81** and 2-aminobenzoxazoles **82**.

The Oxone/NBS-promoted synthesis of 2-aminobenzimidazoles **81** and 2-aminobenzoxazoles **82** was reported in 2016, by Kumar and co-workers, starting from cyclohexanone **83** and guanidine/urea **84** as substrates (Scheme 54) [53]. The Oxone/NBS system promoted the in situ halogenation of cyclohexanone **83**, which reacted with electron-rich and electron-deficient guanidine/urea **84**, affording the respective products **81** and **82** at good to excellent yields. The whole features of this strategy make it a simple and economical approach to prepare 2-aminobenzimidazoles **81** and 2-aminobenzoxazoles **82**.

**Scheme 55:**
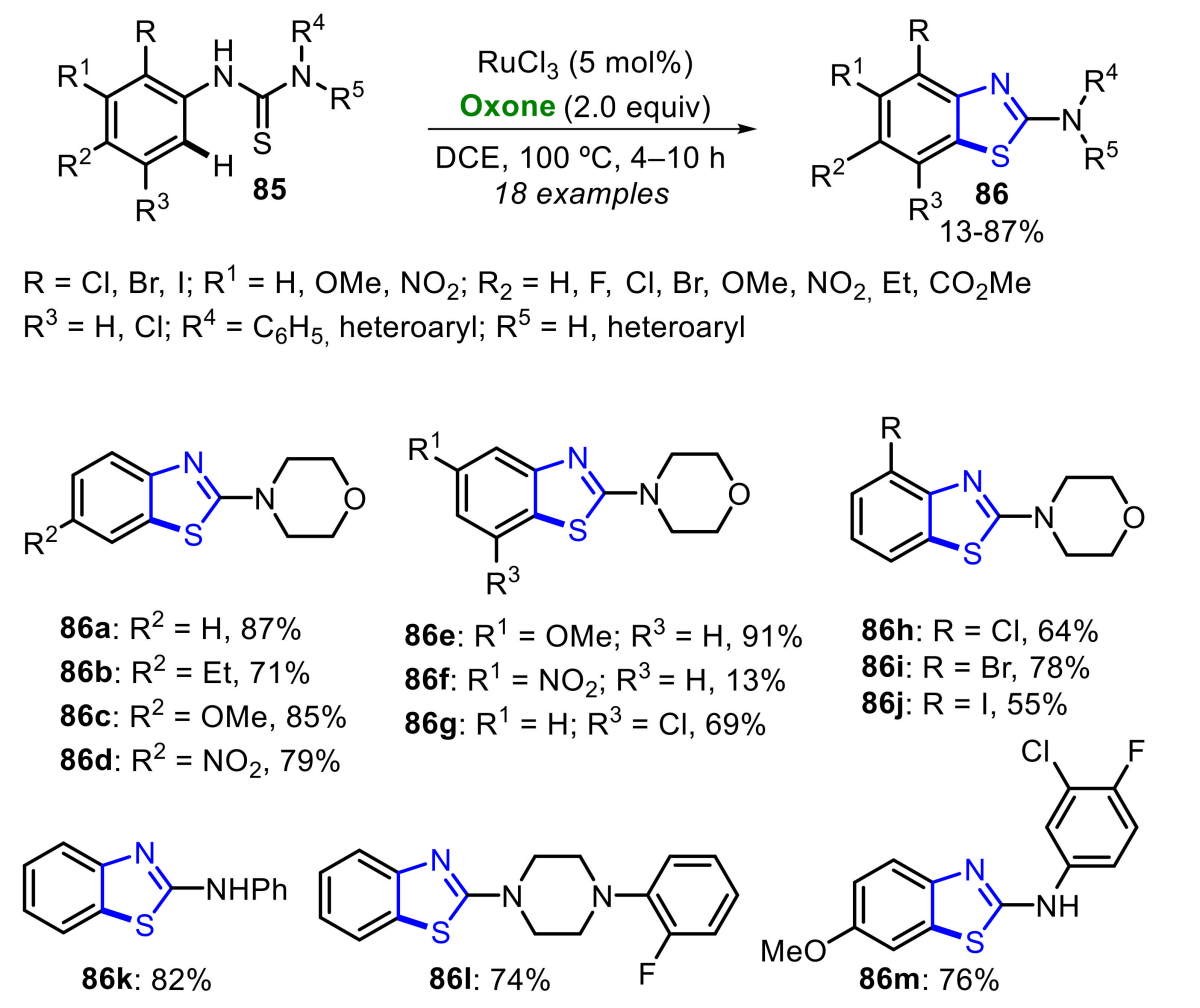
Ru-catalyzed Oxone-mediated intramolecular C−S coupling of *N*-arylthioureas **85**.

In 2016, Sawant and co-workers have reported a Ru-catalyzed Oxone-mediated intramolecular C−S coupling of *N*-arylthioureas **85**, aiming to access 2-aminobenzothiazoles **86** (Scheme 55) [54]. A good substrate tolerance was achieved, allowing the synthesis of a wide library of 2-aminobenzothiazoles **86** in poor to excellent yields. In general, electron-rich *N*-arylthioureas **85** were more reactive in relation to the electron-poor ones. An example of this remarkable difference can be observed by comparing the access to the products **86e** (R^1^ = OMe) and **86f** (R^1^ = NO_2_), which were obtained at a 91% and 13% yield, respectively. This specific reactivity indicates the involvement of an electrophilic ruthenation mechanistic pathway, which is supported by the observed inverse secondary kinetic isotopic effect (KIE) effect and by the computational studies.

**Scheme 56:**
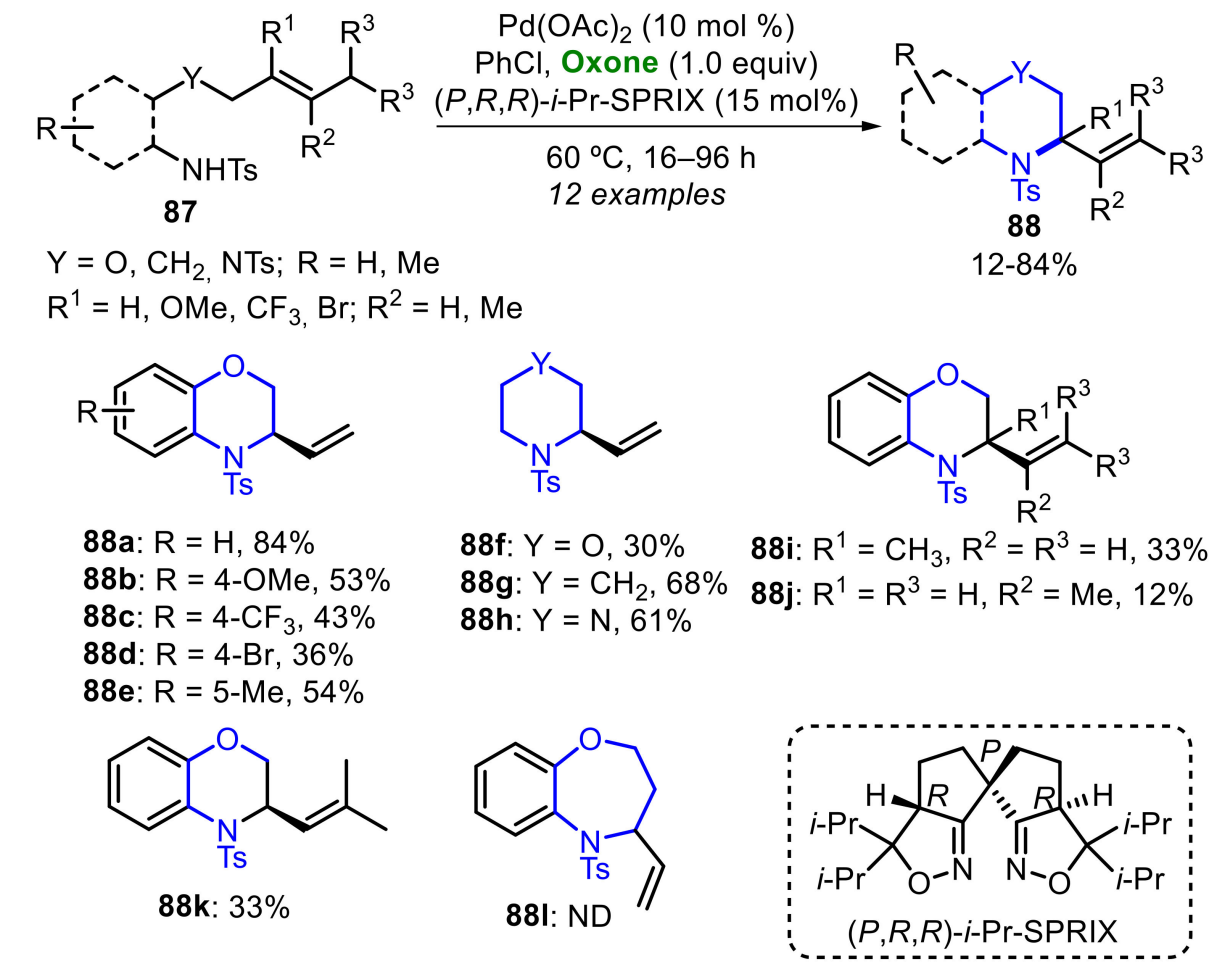
Pd(II)-catalyzed Oxone-mediated enantioselective *aza*-Wacker type reaction.

In 2018, Sasai and co-workers developed a Pd(II)-catalyzed Oxone-mediated enantioselective *aza*-Wacker type reaction of alkenyl sulfonamides **87**, to afford several substituted heterocyclic systems **88**, including morpholines, piperazines and piperidines, as well as their benzo-fused derivatives (Scheme 56) [55]. Electron-rich and electron-deficient alkenyl sulfonamides **87** were adequately employed as substrates to access the products **88b**-**e**, and any remarkable difference on reactivity was observed. The presence of the phenylene binder in the starting material was not mandatory, and the products **88f**–**h** were satisfactorily obtained. Limitations were found when trisubstituted alkenes **87** were used, and the respective products **88i**–**k** were obtained at just a 33%, 12% and 33% yield. The attempts in preparing the benzo[*b*][1,4]oxazepine **88l** were not successful (Scheme 56).

**Scheme 57:**
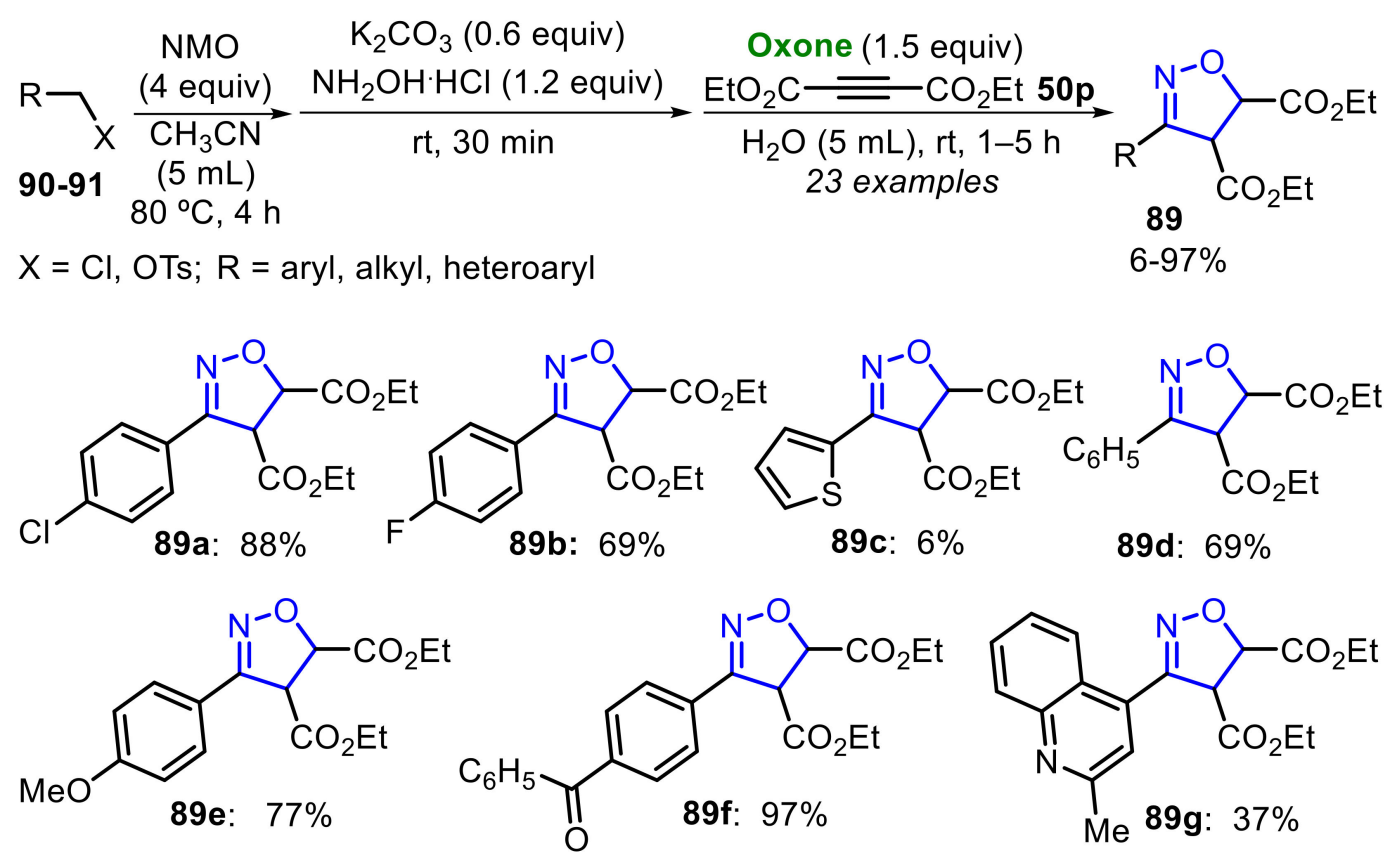
Oxone-mediated synthesis of 3-arylisoxazole-4,5-dicarboxylates **89**, through a *one-pot* process.

In 2018, Imai and Togo reported an Oxone-mediated synthesis of 3-arylisoxazole-4,5-dicarboxylates **89**, through the *one-pot* reaction between benzylic chlorides **80** or alkyl *4*-tosylates **91**, and acetylenedicarboxylate **50p** (Scheme 57) [56]. The substrates **90** and **91** were treated with *N*-methylmorpholine *N*-oxide (NMO, 4 equiv), and then with hydroxylamine hydrochloride (1.2 equiv) and potassium carbonate (0.6 equiv), followed by the addition of acetylenedicarboxylate **50p**, to generate desired products **89**. The method efficiency was proved by accessing a library of twenty-three new isoxazoles **89** at up to a 97% yield and in short reaction times.

**Scheme 58:**
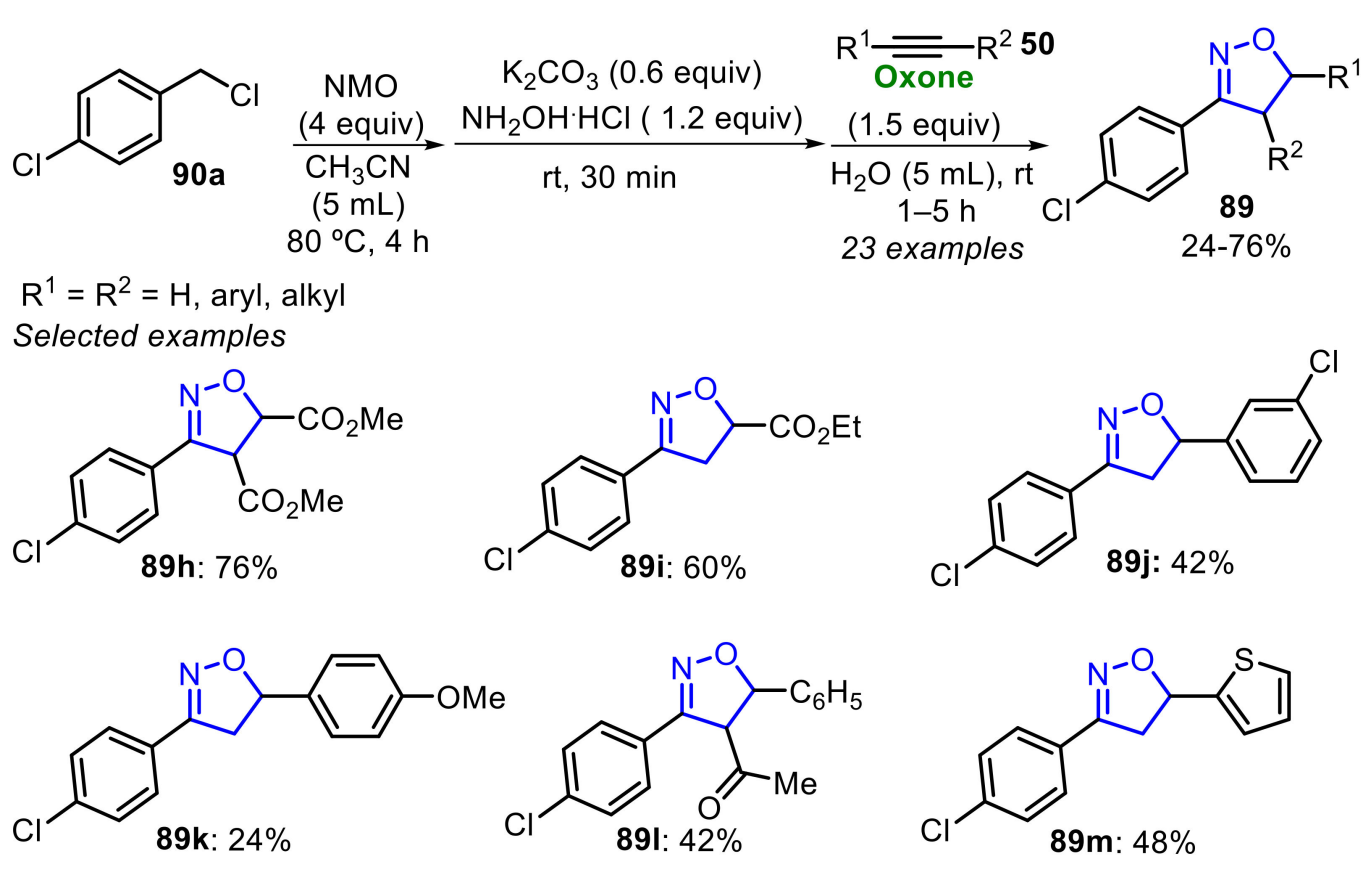
Oxone-mediated synthesis of 3-arylisoxazole-4,5-dicarboxylates **89**, through a *one-pot* process.

The study was extended to different acetylene derivatives **50** in the reaction with 4-chlorobenzyl chloride **90a**, and the desired isoxazoles **89h**–**m** were obtained at moderate to good yields (Scheme 58). As previously observed, a good substrate tolerance was afforded, allowing the application of electron-rich and electron-deficient acetylenes **50**.

**Scheme 59:**
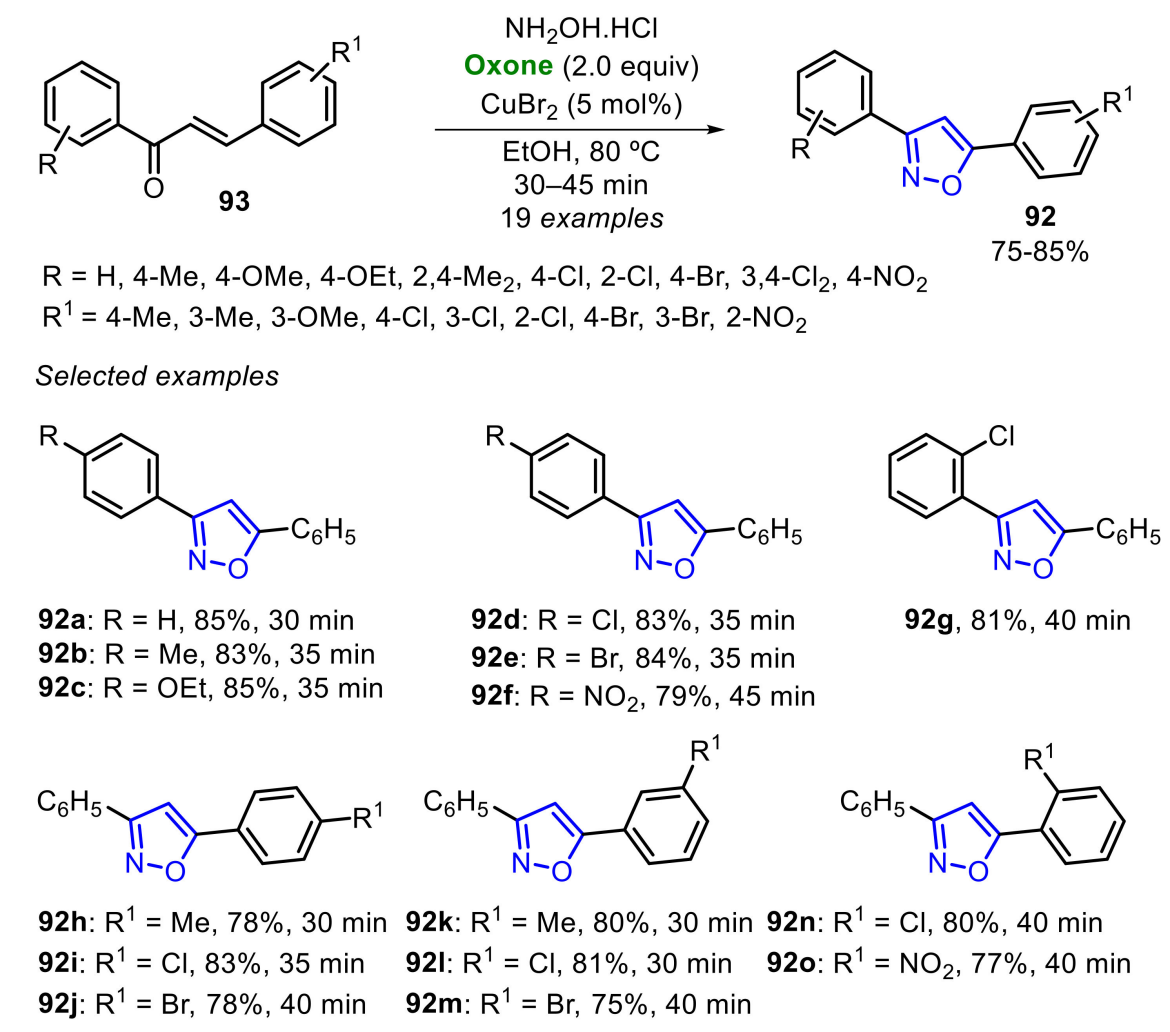
Cu(II)-catalyzed Oxone-mediated *one*-*pot* synthesis of 3,5-diarylisoxazoles **92**.

Bhatt and co-workers reported, in 2019, a Cu(II)-catalyzed Oxone-mediated *one*-*pot* synthesis of 3,5-diarylisoxazoles **92**, starting from *α*,*β*-unsaturated ketones **93** and hydroxylamine hydrochloride, via an oxidative annulation reaction (Scheme 59) [57]. The protocol was successfully applied to a variety of electron-rich and electron-deficient chalcones **93** affording a wide collection of compounds **92a**–**o** in good to excellent yields, just after a few minutes under thermal conditions (refluxing EtOH).

**Scheme 60:**
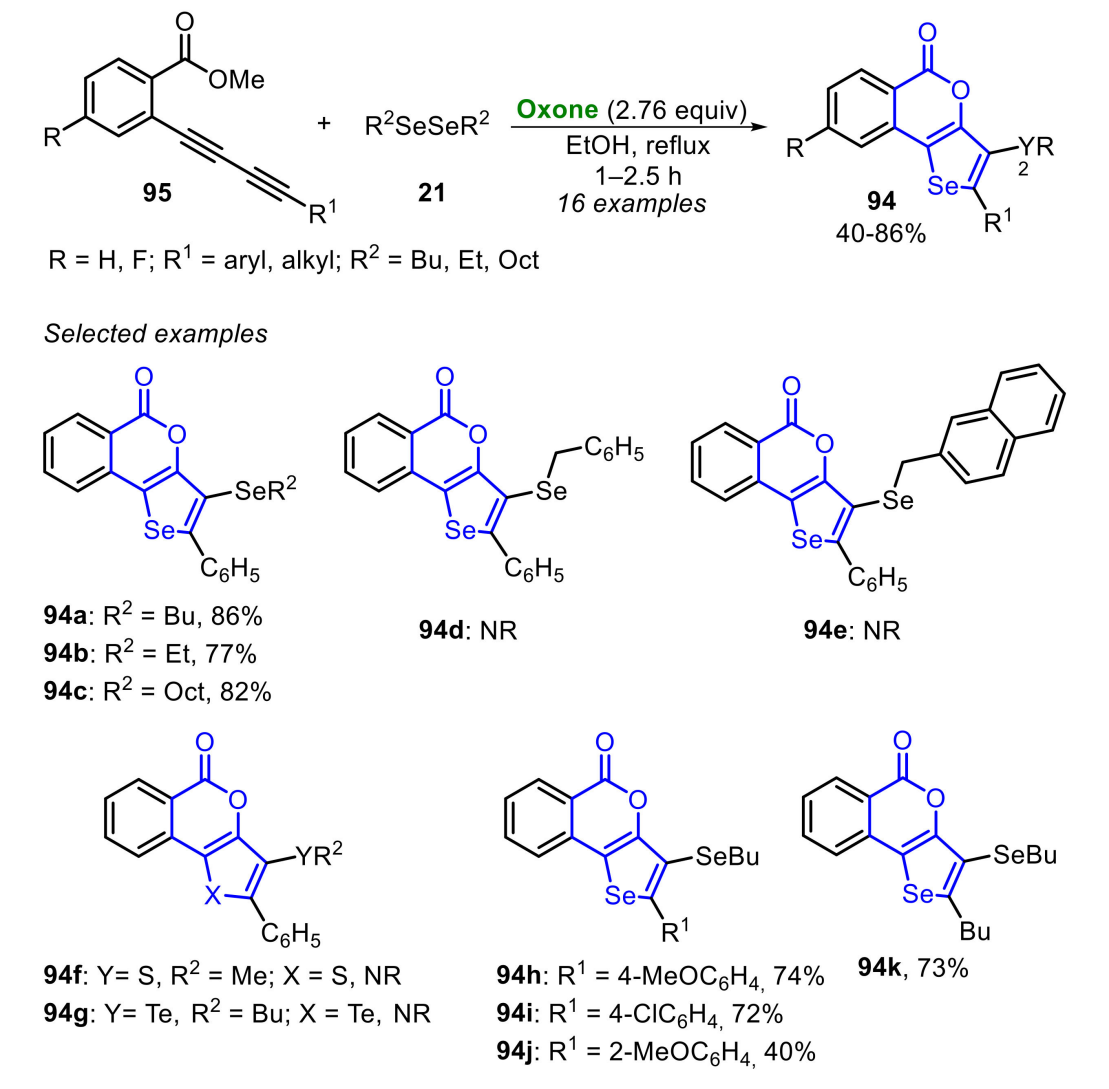
Oxone/dialkyl diselenide-promoted synthesis of 5*H*-selenopheno[3,2-*c*]isochromen-5-ones **94**.

In 2019, some of us reported an Oxone/dialkyl diselenide-promoted synthesis of 5*H*-selenopheno[3,2-*c*]isochromen-5-ones **94**, through a double intramolecular cyclization of methyl 2-(organyl-1,3-diynyl)benzoate **95** (Scheme 60) [58]. Dialkyl diselenides **21** (R^2^ = Et, Bu and Oct) were satisfactorily employed, giving the corresponding isochromenones **94a**–**c** in good yields, whereas dibenzyl diselenide, bis(2-naphthylmethyl)diselenide, dibutyl ditelluride and dimethyl disulfide were not suitable to produce the products **94d**, **94e**, **94f** and **94g**, respectively. Additionally, several substituted 1,3-diynes were efficiently employed as substrates, affording the products **94h**–**k** at moderate to good yields (Scheme 60). 

**Scheme 61:**
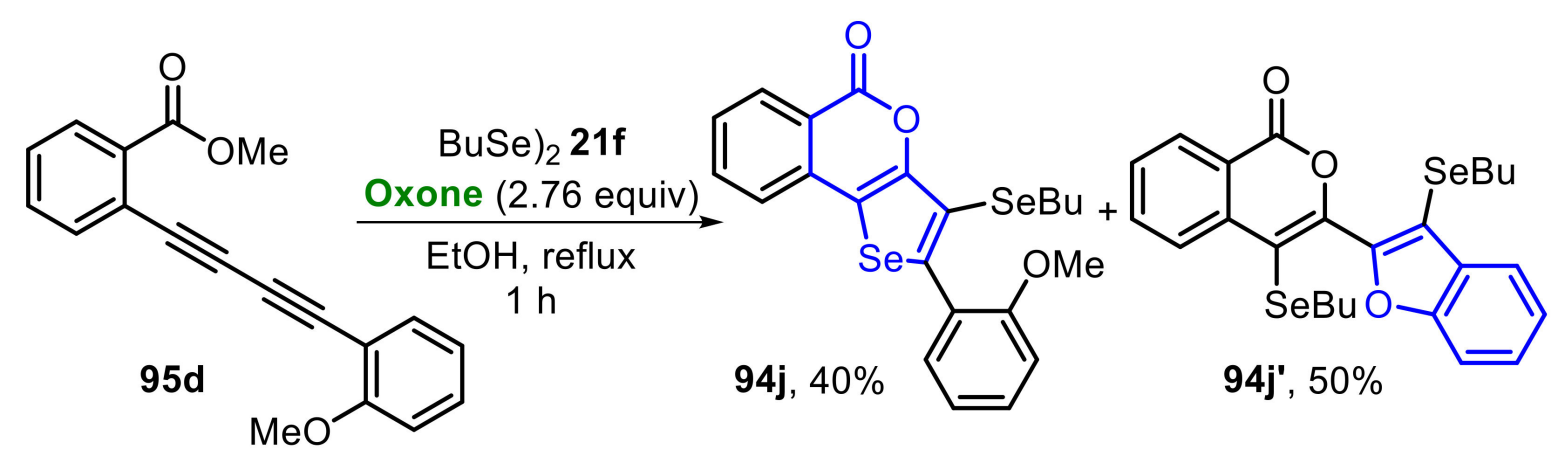
Competition between Se-cyclization and *O*-cyclization of the substrate **95d**.

**Scheme 62:**
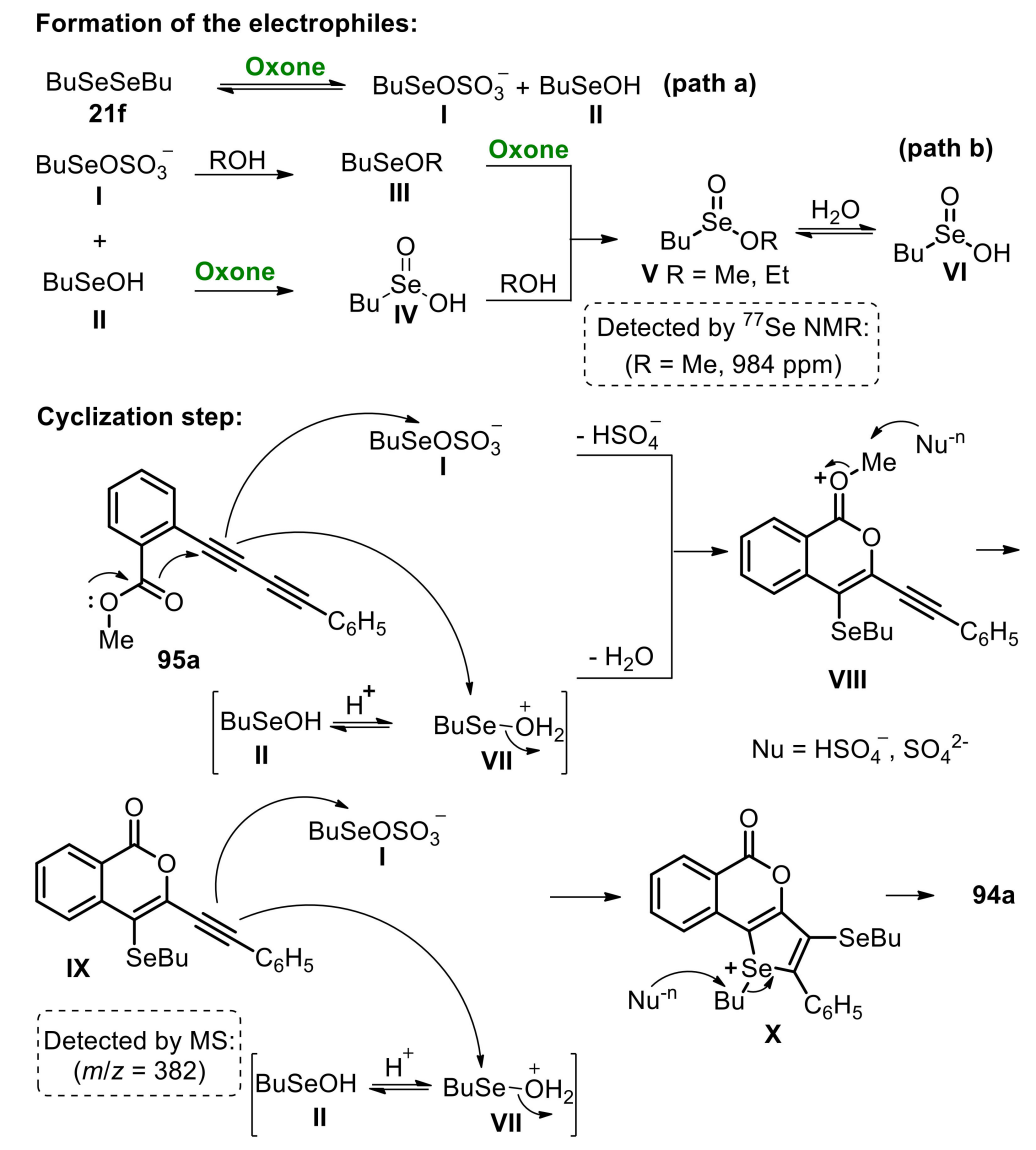
Plausible reaction mechanism for the formation of the products **94**.

Curiously, when 2-(2-methoxyphenylbuta-1,3-diynyl)benzoate **95d** was employed as substrate, in the presence of dibutyl diselenide **21f**, the expected 5*H*-selenopheno[3,2-c]isochromen-5-one **94j** was isolated in only 40% yield. The low efficiency toward **94j** is associated with the parallel formation of benzofuran derivative **94j′**, that was obtained at a 50% yield (Scheme 61). This behavior is due to two competing reactions (Se-cyclization vs. *O*-cyclization) of the intermediate **IX**, generated in the first step of the transformation (see Scheme 62, for the reaction mechanism).

Based on control experiments, which were monitored by ^77^Se NMR and GC/MS analysis, a possible mechanism for the reaction was proposed (Scheme 62). Initially, dibutyl diselenide **21f** reacts with Oxone to form two intermediates: BuSeOSO_3_^–^ **I** and BuSeOH **II** (Scheme 62, path a). The formation of species **I** and **II** was confirmed by the formation of seleninic ester **V** and acid **VI** (Scheme 62, path b). The species **II** can be protonated by the reaction medium, leading to BuSeOH_2_^+^ **VII**. Then, the 1,3-diyne **95a** reacts with **I** and **VII** to form the cyclic intermediate **VIII**, releasing HSO_4_^-^ and H_2_O to the reaction medium. Following, the displacement of the methyl group from **VIII**, by a nucleophile (HSO_4_^-^ and/or SO_4_^2-^) affords the intermediate **IX** (detected by mass spectrometry), that reacts similarly with **I** and **VII**, to produce the fused-selenophene cation intermediate **X**. The displacement of the butyl group finally affords the expected product **94a** (Scheme 62).

**Scheme 63:**
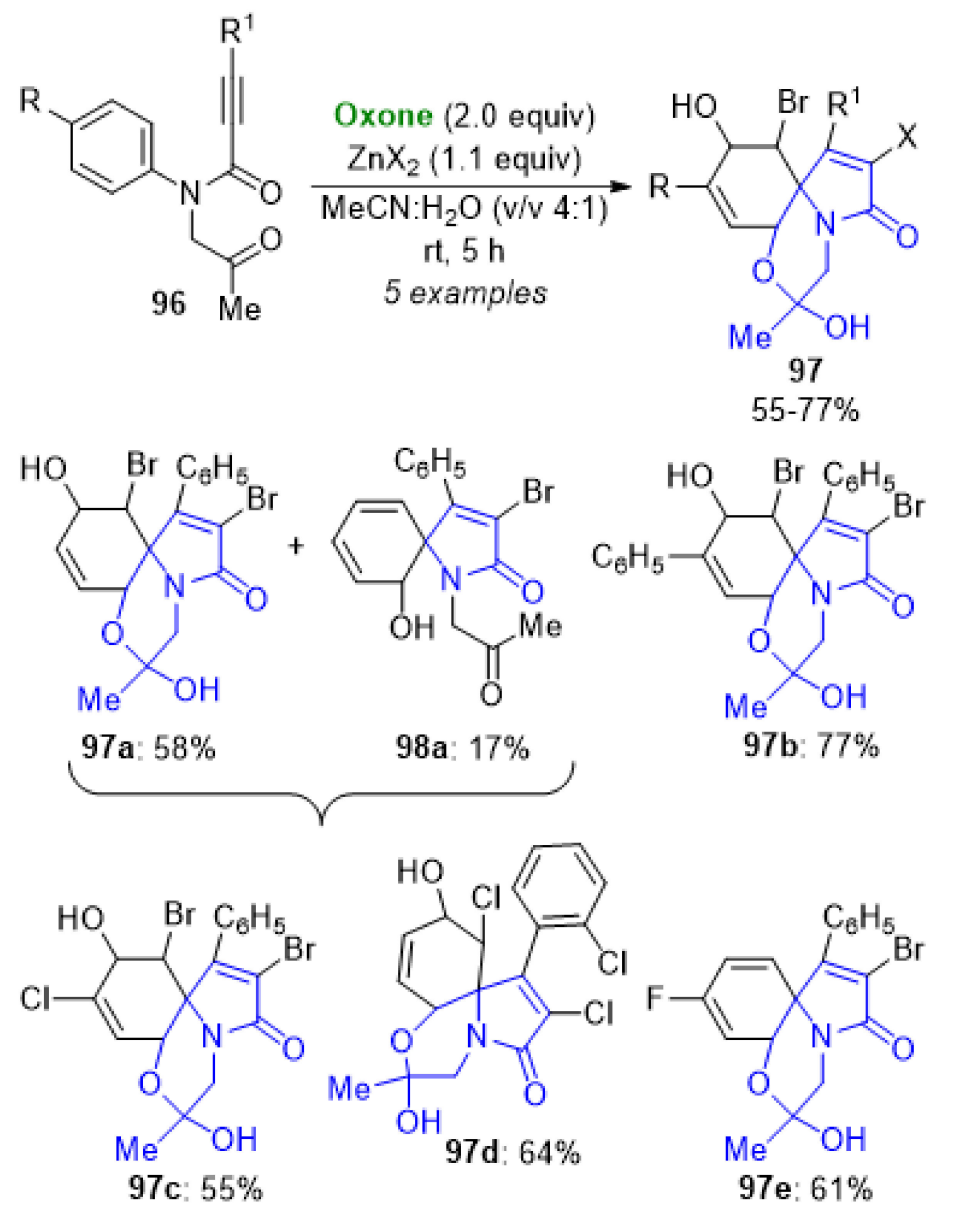
Oxone-mediated *ipso*-cyclization of *N*-arylpropiolamide **93** to access the spirocyclo derivatives **97**.

In 2020, Qiu, Liu and co-workers developed the Oxone-mediated *ipso*-cyclization of *N*-arylpropiolamide **96** to access the spirocyclo derivatives **97** and **98** (Scheme 63). The reaction was conducted in the presence of MeCN:H_2_O (*v*/*v* 4:1) as reaction medium, and the selectivity was achieved by employing different halogen sources (ZnX_2_ and TBAX) [59]. In the presence of ZnX_2_, the spiro-tricycle **97** was formed, through an *ortho*-hydroxylative process, in moderate to good yields. By employing *N*-(2-oxopropyl)-*N*,3-diphenylpropiolamide **96a**, the formation of a mixture of the products **97a** and **98a** was observed. When *para*-substituted substrates **96** (R = C_6_H_5_ and Cl) were used, the expected products **97b**–**c** were obtained at good yields, without formation of the ring-opened derivatives. Interestingly, the fluoro-substituted substrate **96** was suitable to the reaction conditions, giving the respective product **97e** at a 61% yield (Scheme 63).

**Scheme 64:**
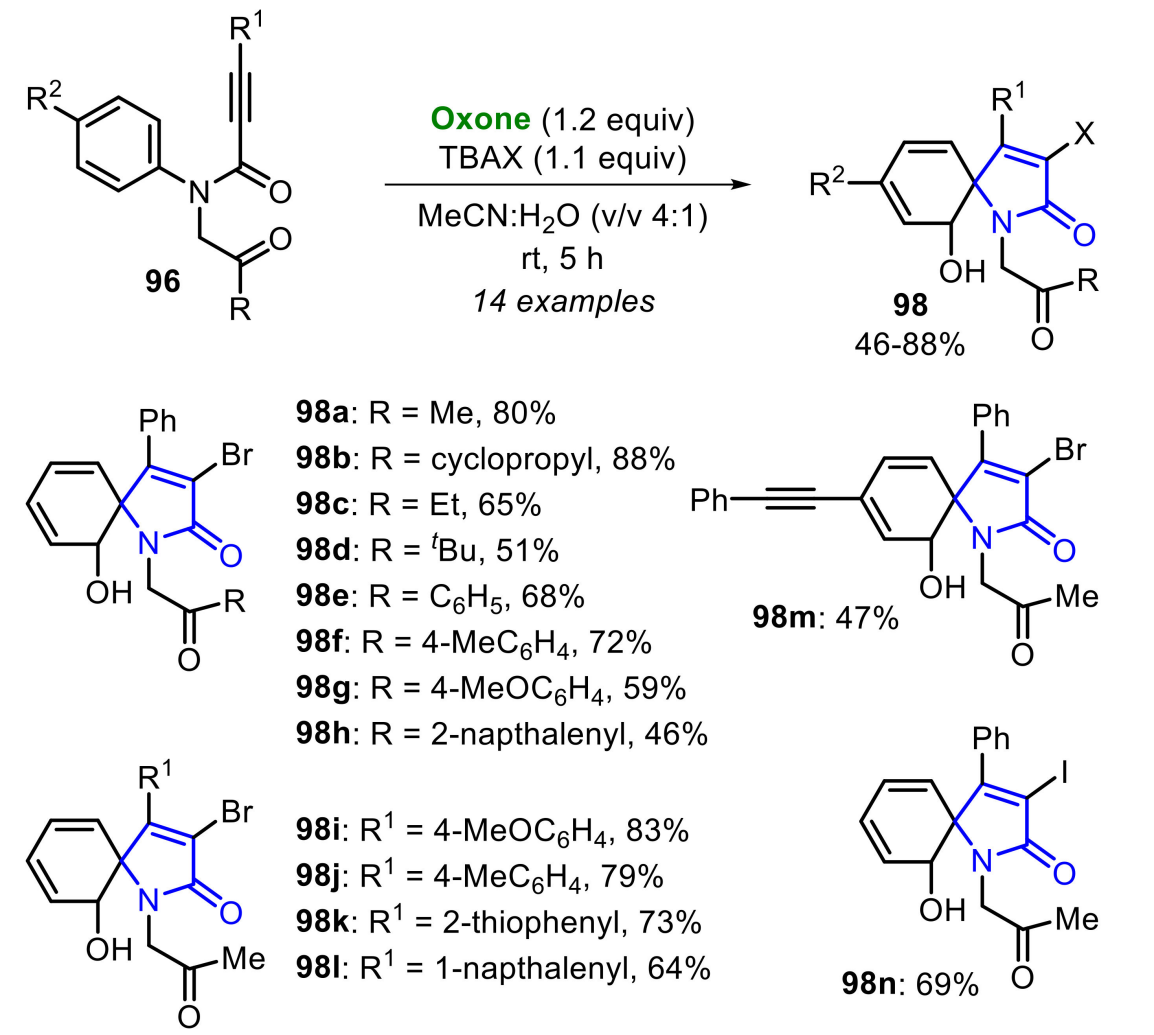
Oxone-mediated *ipso*-cyclization of *N*-arylpropiolamide **96** to access the spirocyclo derivatives **98**.

Due to the poor stability of spiro-tricycles **97**, authors have focused their attention on the synthesis of ring-opened products **98**, that were accessed by employing TBAX as halogen source, and the protocol was extended to *N*-arylpropiolamide **96** bearing different R, R^1^ and R^2^ substituents (Scheme 64). The R group can be replaced by a cyclopropyl ketone, ethyl ketone, *^t^*butyl ketone, and several aryl ketones, giving a range of ortho-hydroxy spirocycles **98a h** in 46–88% yields. Additionally, the substituent R^1^ can be easily replaced by aryl or heteroaryl groups, leading to the respective products **98i**–**l** in good yields. By employing the *para*-alkynyl *N*-arylpropiolamide **96**, the product **98m** was obtained at only a 47% yield. Moreover, TBAI has also demonstrated to be efficient, allowing the synthesis of the compound **98n** at a 69% yield.

**Scheme 65:**
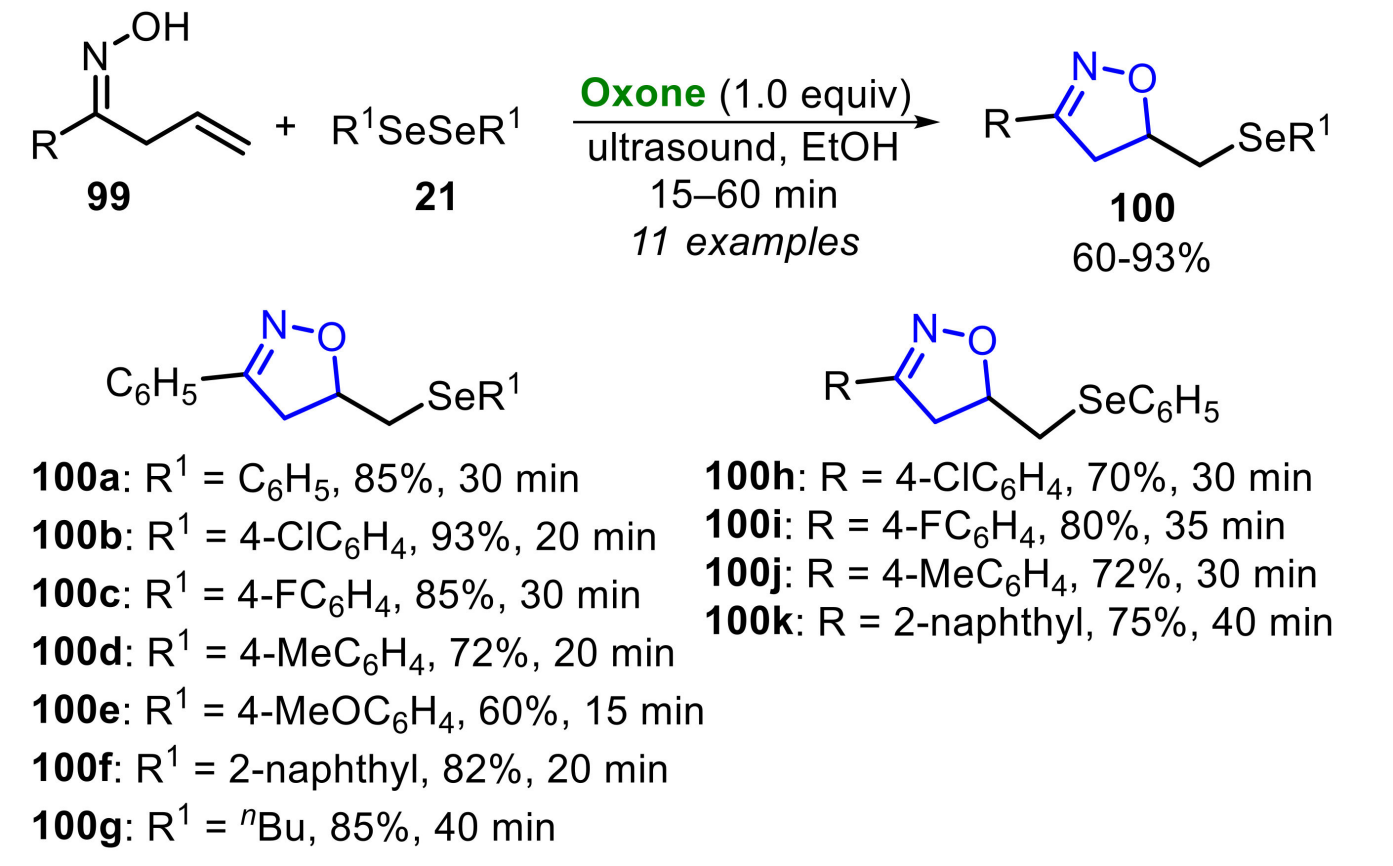
Oxone/diselenide-promoted ultrasound-assisted selective cyclization of unsaturated oximes **99**.

In 2020, some of us described the Oxone/diselenide-promoted ultrasound-assisted selective cyclization of unsaturated oximes **99**, to access 5-methylselanyl-4,5-dihydroisoxazoles **100** (Scheme 65) [60]. The sonication was crucial to drive the process selectively, in short reaction times, giving the products **100** in moderate to excellent yields. Several oximes **99** and diselenides **21**, bearing electron-donating or electron-withdrawing groups, were satisfactorily employed, proving the applicability and substrate tolerance of the method. 

**Scheme 66:**
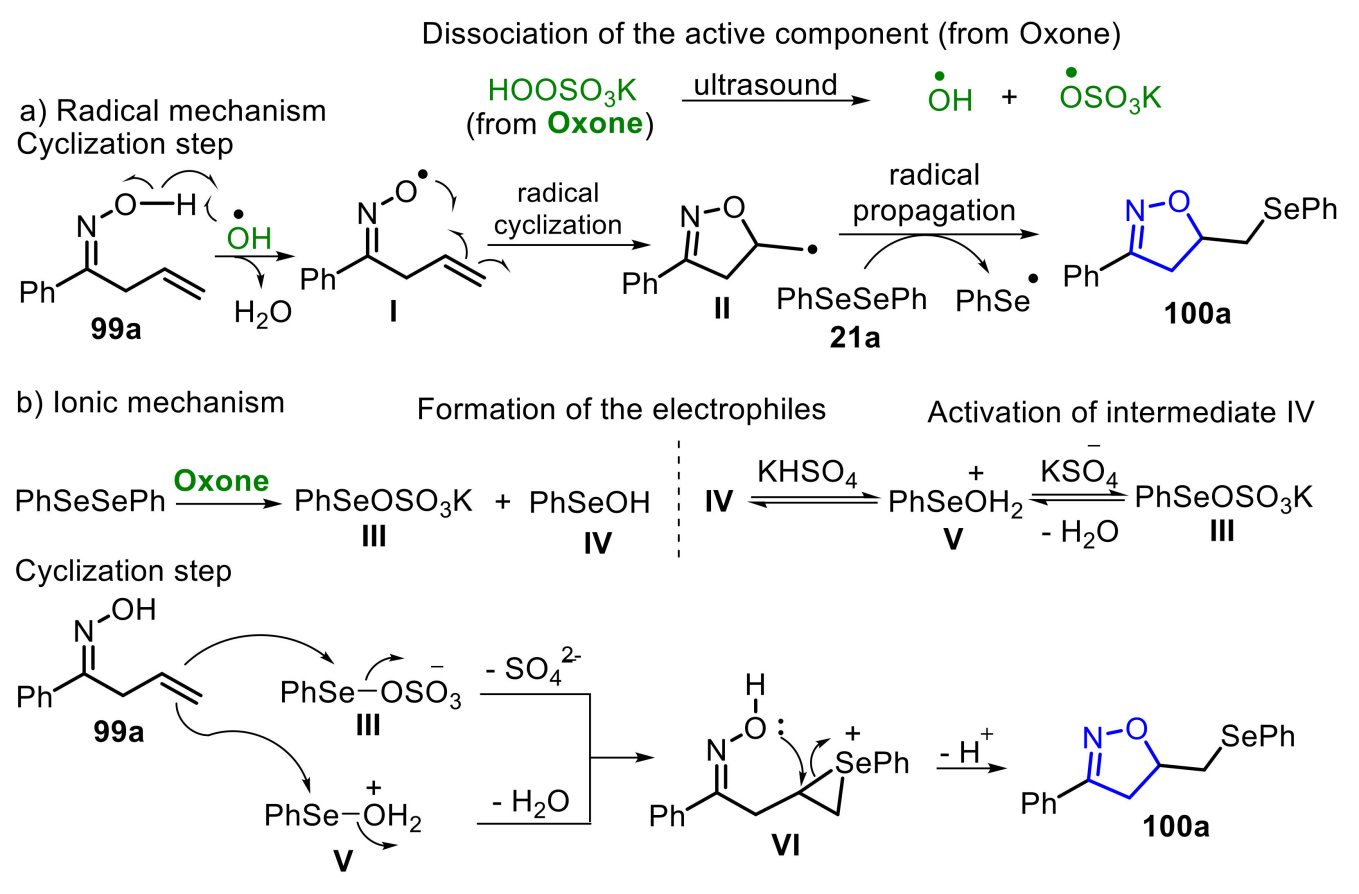
Plausible reaction mechanism for the Oxone/diselenide-promoted ultrasound-assisted selective cyclization of unsaturated oximes **99**. **a**: Radical mechanism. **b**: Ionic mechanism.

A comparison study, employing different energy sources (conventional heating, microwave and ultrasound irradiation) was conducted by the authors. Under ultrasound irradiation the process followed a radical pathway, as verified by control experiments. On the other hand, by employing conventional heating (65 °C) or microwave irradiation, the transformation followed an ionic pathway. Regarding the radical pathway, the first step is the formation of the hydroxyl radical (HO•) and the radical species •OSO_3_K, through the US-promoted Oxone dissociation. Once formed, the hydroxyl radical reacts with oxime **99a** to form H_2_O and the *O*-centered radical intermediate **I**, which undergoes a radical cyclization, delivering the 5-methyl-4,5-dihydroisoxazole radical **II**. Following, the intermediate **II** reacts with diphenyl diselenide **21a** to yield the desired product **100a**, while releasing a Se-centered radical species, that is recovered as diselenide **21** (Scheme 66a). Regarding the ionic mechanism, the first step is the Oxone-promoted formation of the Se-based electrophilic species **III** and **IV**, by the Se–Se bond oxidation. The species **IV** is protonated to produce the most electrophilic species **V**. Then, the oxime **99a** reacts with **III** and/or **V** to be converted to the seleniranium intermediate **VI**, which undergoes an intramolecular cyclization process, followed by deprotonation and formation of the desired product **100a** (Scheme 66b).

**Scheme 67:**
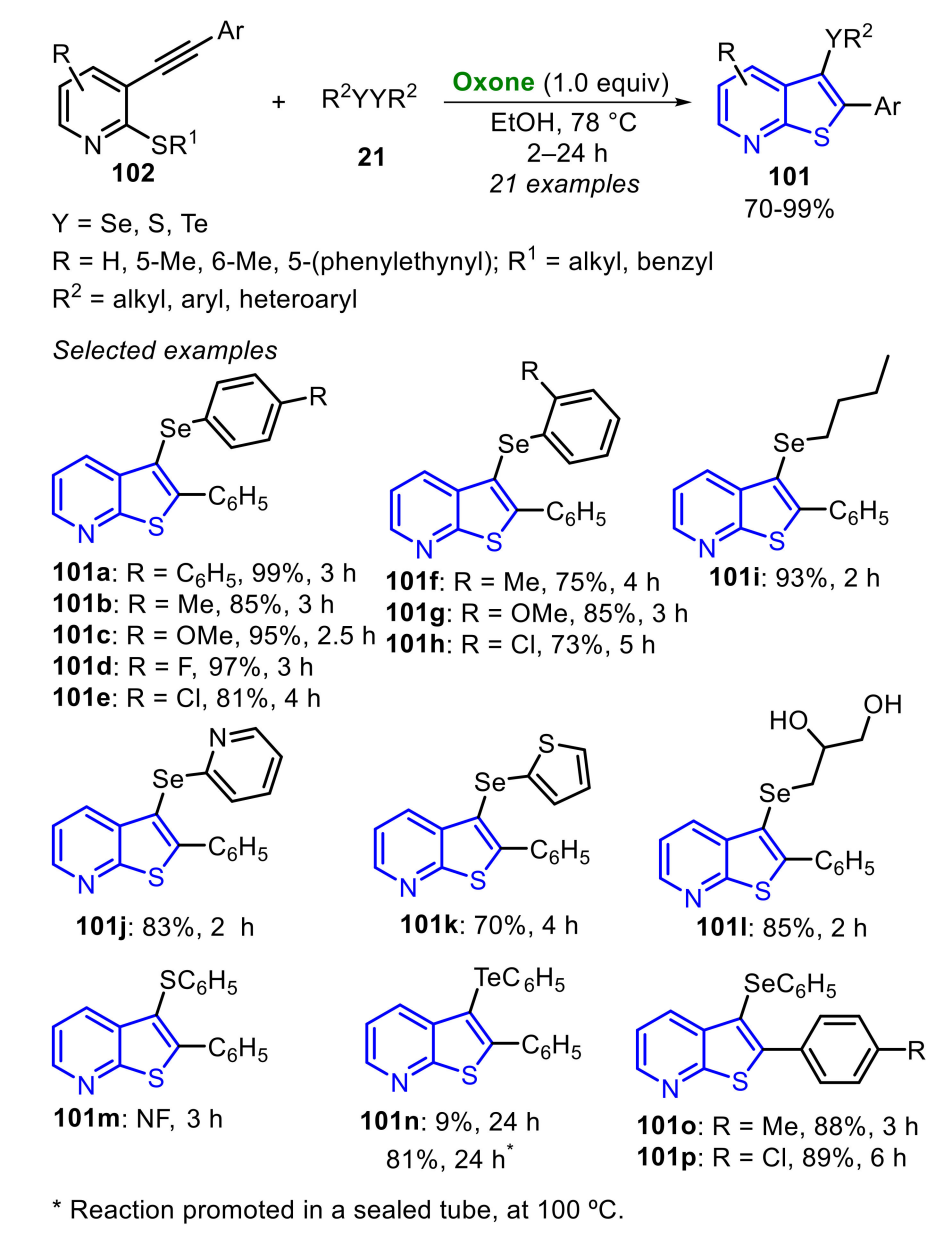
Oxone/diselenide-promoted synthesis of 2-aryl-(3-organocalcogenyl)thieno[2,3-*b*]pyridines **101**.

In 2021, some of us described the Oxone/diselenide-promoted synthesis of 2-aryl-(3-organocalcogenyl)thieno[2,3-*b*]pyridines **101** through the electrophilic cyclization of 3-(arylethynyl)-2-(alkylthio)pyridines **102** (Scheme 67) [61]. A number of electron-rich and electron-deficient diaryl diselenides **21** were adequately employed as substrate, affording the products **101a**–**h** at good to excellent yields. Additionally, alkyl and heteroaryl diselenides **21** were suitable substrates, giving the respective products **101i**–**k** at good to excellent yields. When the glycerol-derived diselenide was used, the unprotected glycol **101l** was obtained at an 85% yield, due to the Oxone-promoted ketal deprotection ability. No reaction was observed using diphenyl disulfide as chalcogen source, whereas diphenyl ditelluride was discretely converted to **101n** at only a 9% yield after 24 h. Aiming to improve this resulted, the reaction was conducted in a sealed tube at 100 °C, yielding the tellurium-containing product **101n** at 81%, after 24 h. Regarding the 3-(arylethynyl)-2-(propylthio)pyridines **102** counterpart, electron-rich (R = 4-MeC_6_H_4_) and electron-deficient (R = 4-ClC_6_H_4_) substrates were satisfactorily used, giving the products **101o** and **101p** at a 88% and 89% yield, respectively (Scheme 67).

**Scheme 68:**
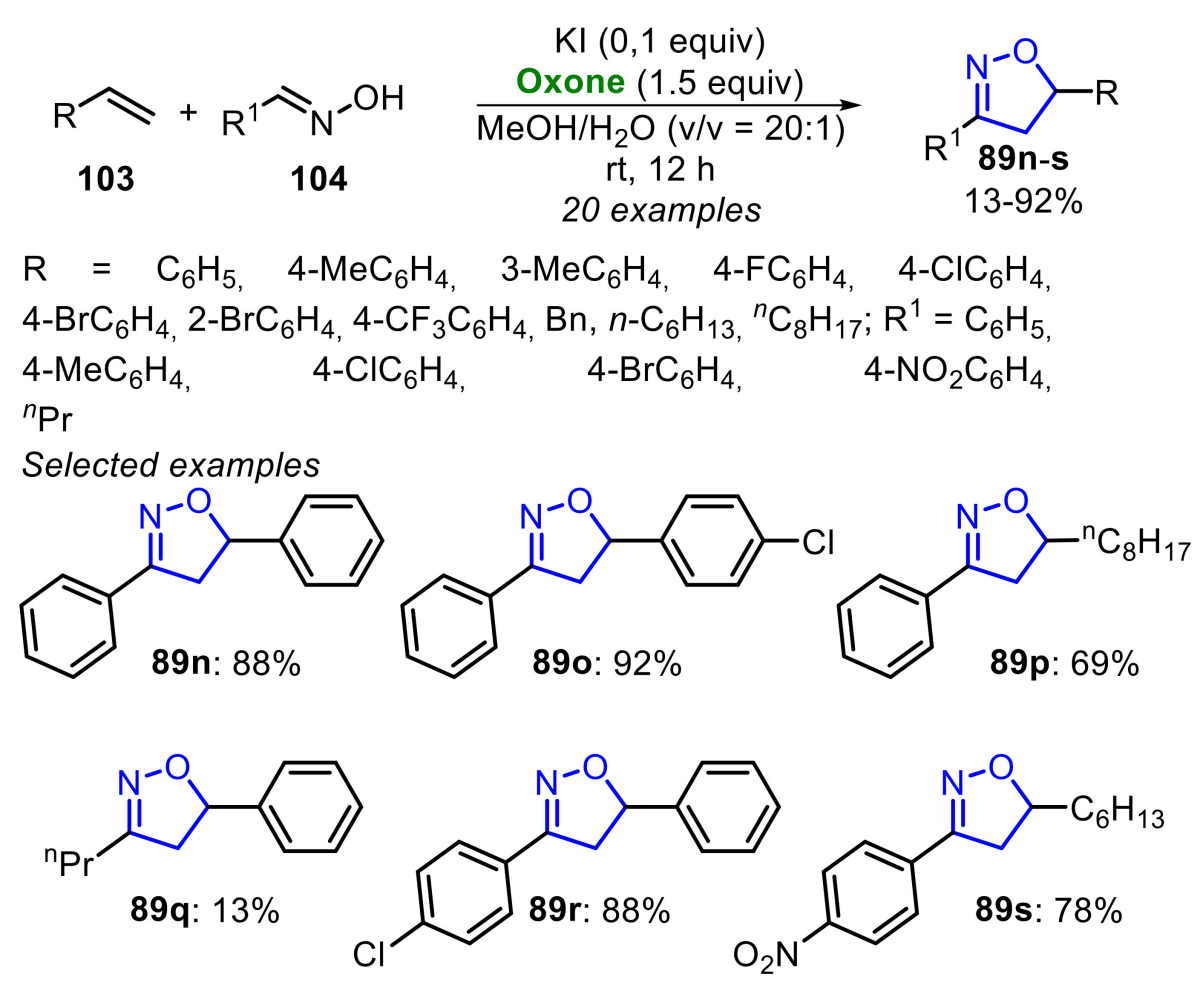
Catalytic cyclization of alkenes **103** with aldoximes **104**.

In 2013, Zhdankin and co-workers [62] described the reaction between alkenes **103** and aldoximes **104** using KI/Oxone catalysis to obtain isoxazolines **89n**–**s** (Scheme 68). The reaction took place at room temperature, using a mixture of MeOH/H_2_O (*v*/*v* = 20:1), and after 12 h of reaction, the expected isoxazolines **89n**–**s** were obtained in yields ranging from 13% to 92%. The method was extended to several alkenes and aldoximes containing electron donating and electron withdrawing substituents attached to the aromatic ring. It was observed that when the aliphatic aldoxime **104d** was used, the desired product **89q** was obtained at only 13% of the yield (Scheme 68).

**Scheme 69:**
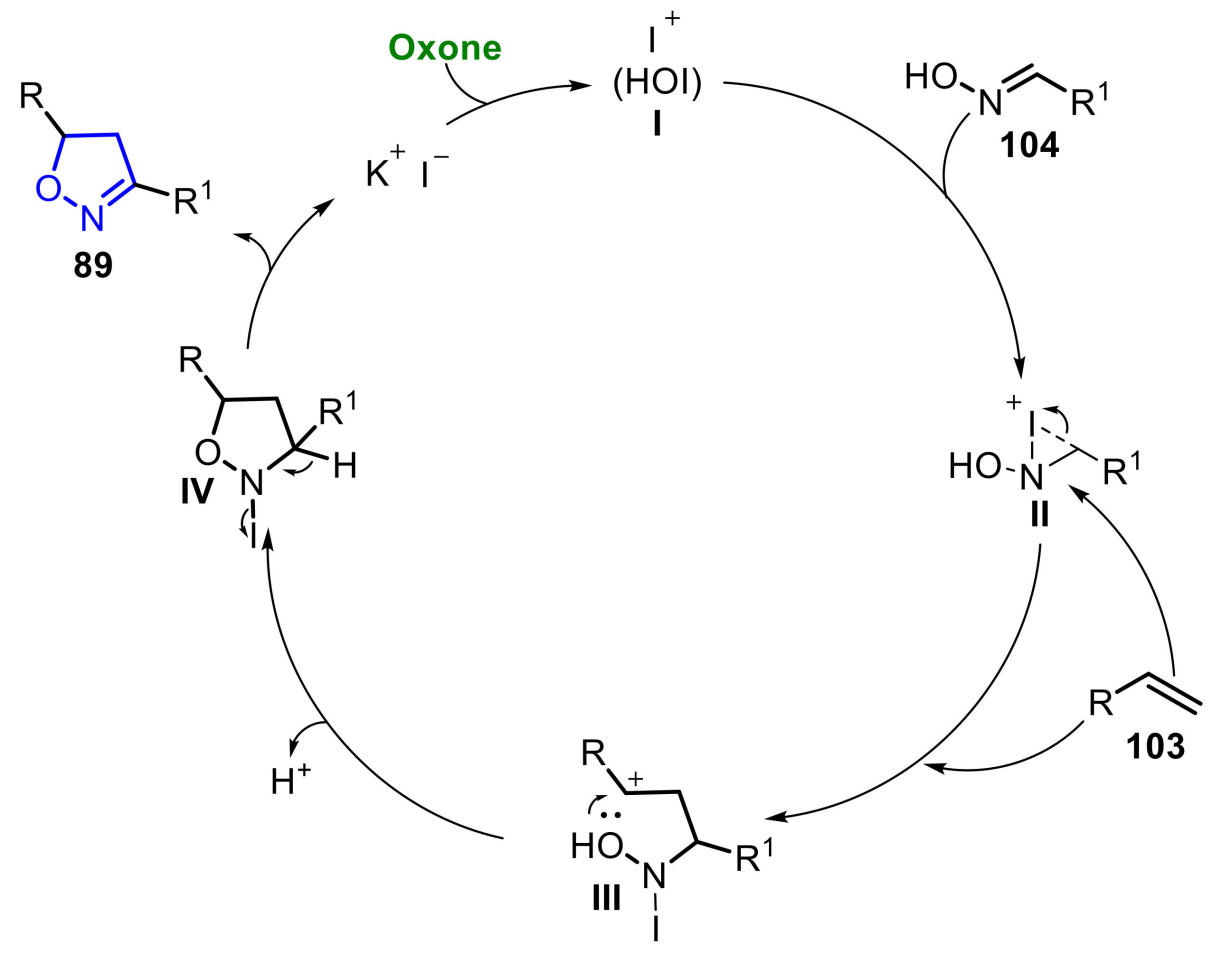
Mechanism proposed for the synthesis of isoxazoles **89n**–**s**.

The proposed reaction mechanism starts with the formation of the active species of iodine (hypoiodous acid) **I**, from the reaction of KI (0.1 equiv) and Oxone (1.5 equiv). This species reacts with aldoxime **104**, forming the iodonium ion intermediate **II**, which is then opened by the alkene **103** to produce the *N*-iodohydroxy intermediate **III**. Finally, a cyclization occurs, forming the *N*-iodo-isoxazolidine intermediate **IV**, which undergoes a *β*-elimination to afford the isoxazoline **89** of interest (Scheme 69).

**Scheme 70:**
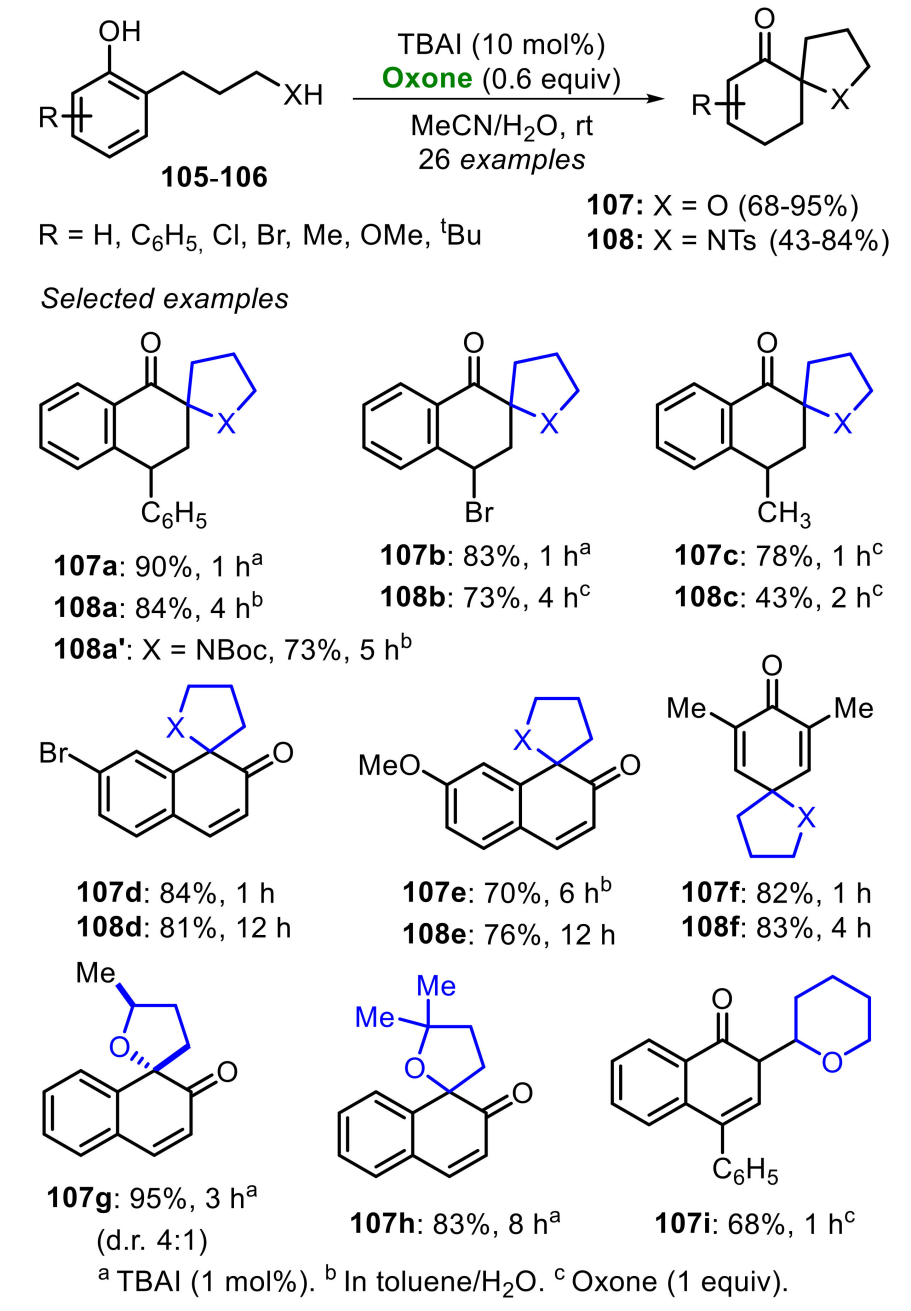
I^+^/Oxone catalyzed oxidative spiroetherification and spiroamination of phenols **105** and **106**.

In 2020, Ishihara and co-workers developed an oxidative spiroetherification and spiroamination of phenol derivatives **105** and **106** using I^+^/Oxone catalysis (Scheme 70) [63]. In the presence of TBAI (10 mol%), Oxone (0.6 equiv), and a mixture CH_3_CN/H_2_O as solvent, arenols tethered to a hydroxy group **105** or a secondary amido group **106** were converted to corresponding spiro adducts **107** or **108** at moderate to excellent yields, at room temperature. The protocol was successfully applied to a variety of arenols **105** and **106**, including 1- and 2-naphthols and phenols, tethered to hydroxy or sulfonamido groups. The oxidative cyclization of secondary and tertiary alcohols **105g** and **105h** was also demonstrated and the corresponding spiroethers **107g** and **107h** were obtained in good to excellent yields. Interesting, six-membered spiroether **107i** could be synthesized at a 68% yield (Scheme 70).

**Scheme 71:**
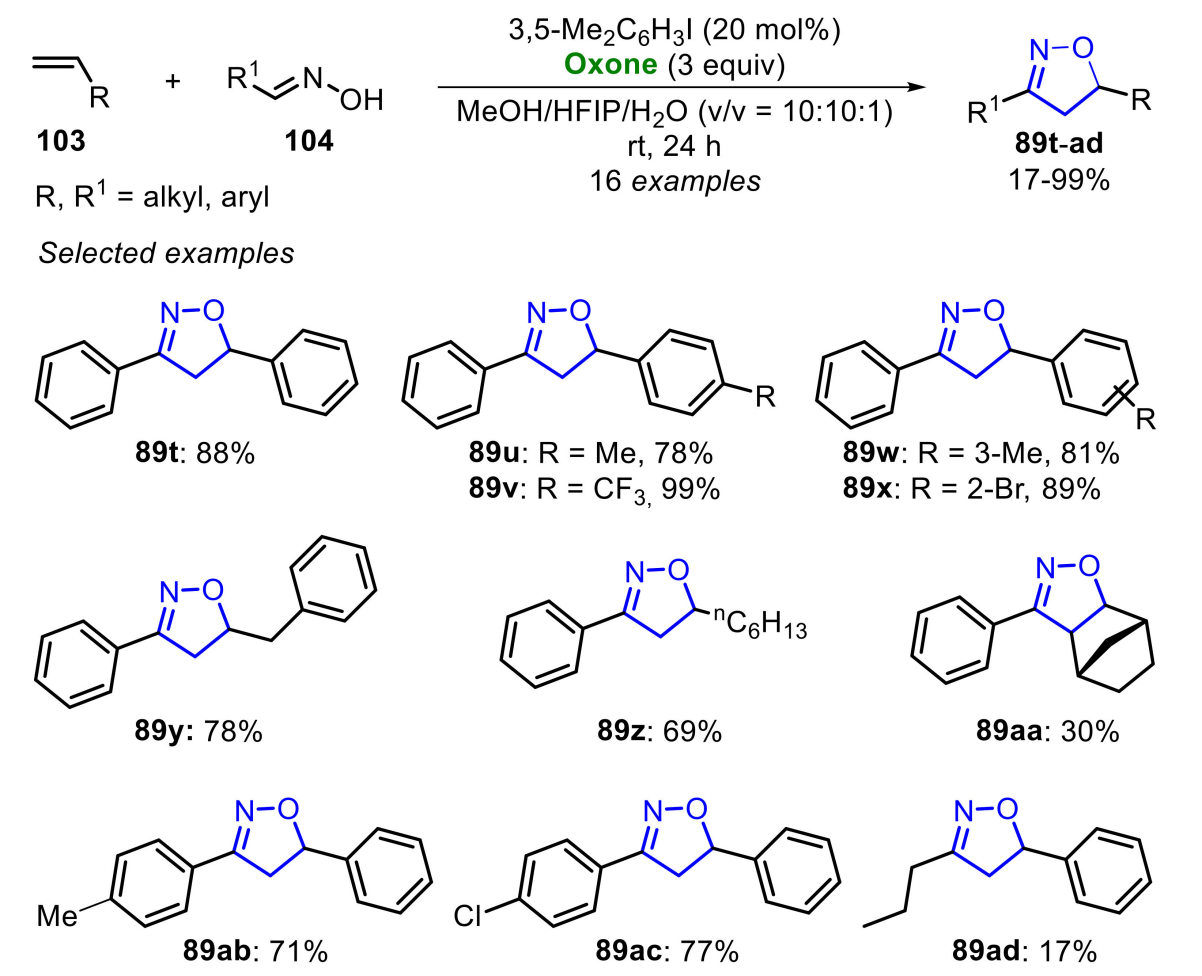
Oxone-mediated synthesis of isoxazolines **89t**-**ad**.

**Scheme 72:**
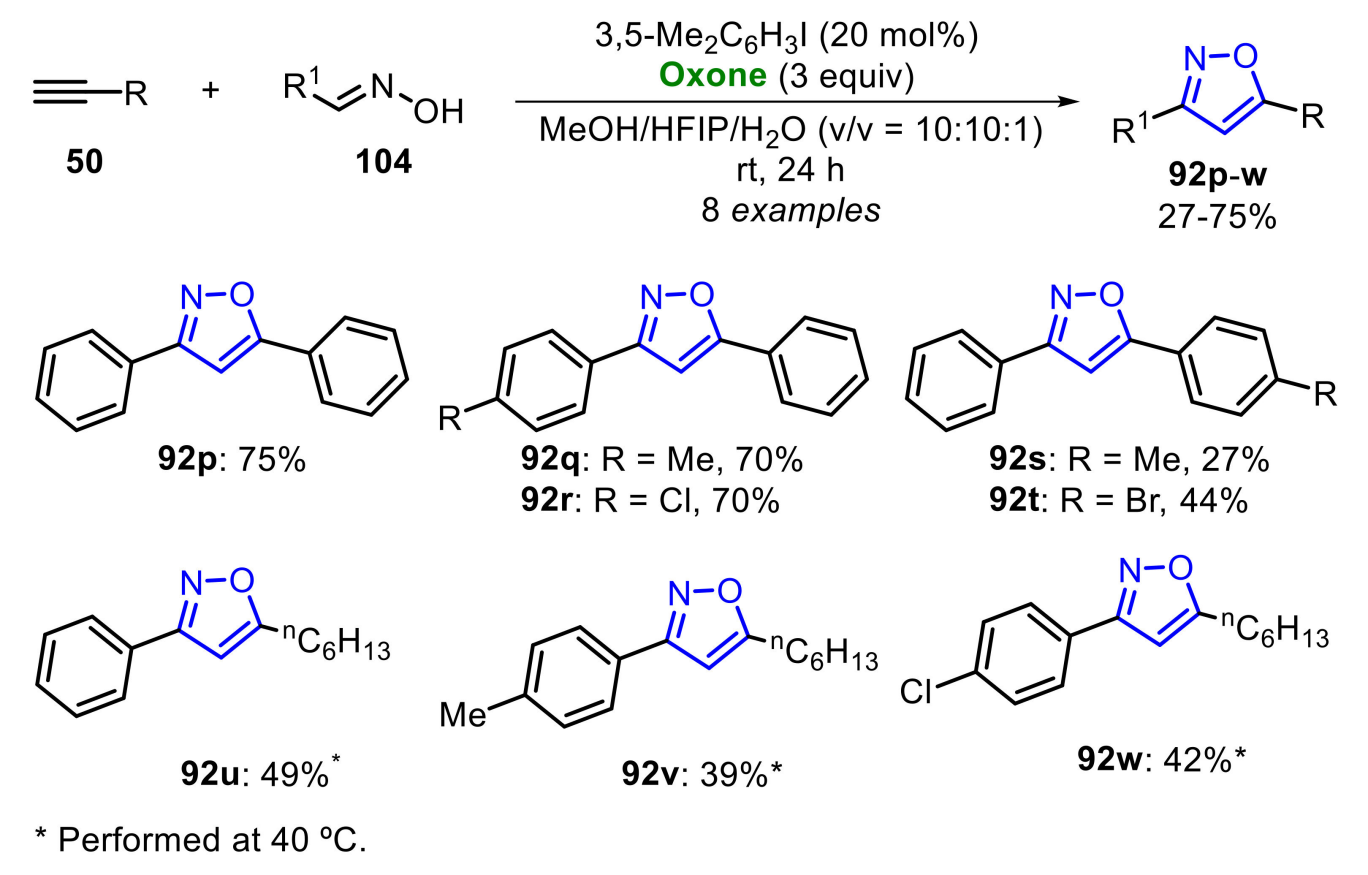
Oxone-mediated synthesis of isoxazoles **92p**–**w**.

In 2013, Yoshimura, Zhdankin and co-workers reported the cyclization of aldoxime **104** and alkene **103** or alkyne **50** using catalytic hypervalent iodine, Oxone as a terminal oxidant, and a mixture of MeOH:HFIP:H_2_O (*v*/*v* = 10:10:1) as solvent at room temperature for 24 h [64]. The reaction involves the initial formation of nitrile oxides, which react with alkenes and alkynes to produce the corresponding isoxazolines **89t-ad** (Scheme 71) and isoxazoles **92p**–**w** (Scheme 72). The protocol was expanded to aldoximes **104** and alkenes **103** or alkynes **50** containing several electron-rich and electron-deficient aryl groups and alkyl groups. By this procedure, a total of sixteen isoxazolines **89** and eight isoxazoles **92** were obtained in poor to excellent yields.

**Scheme 73:**
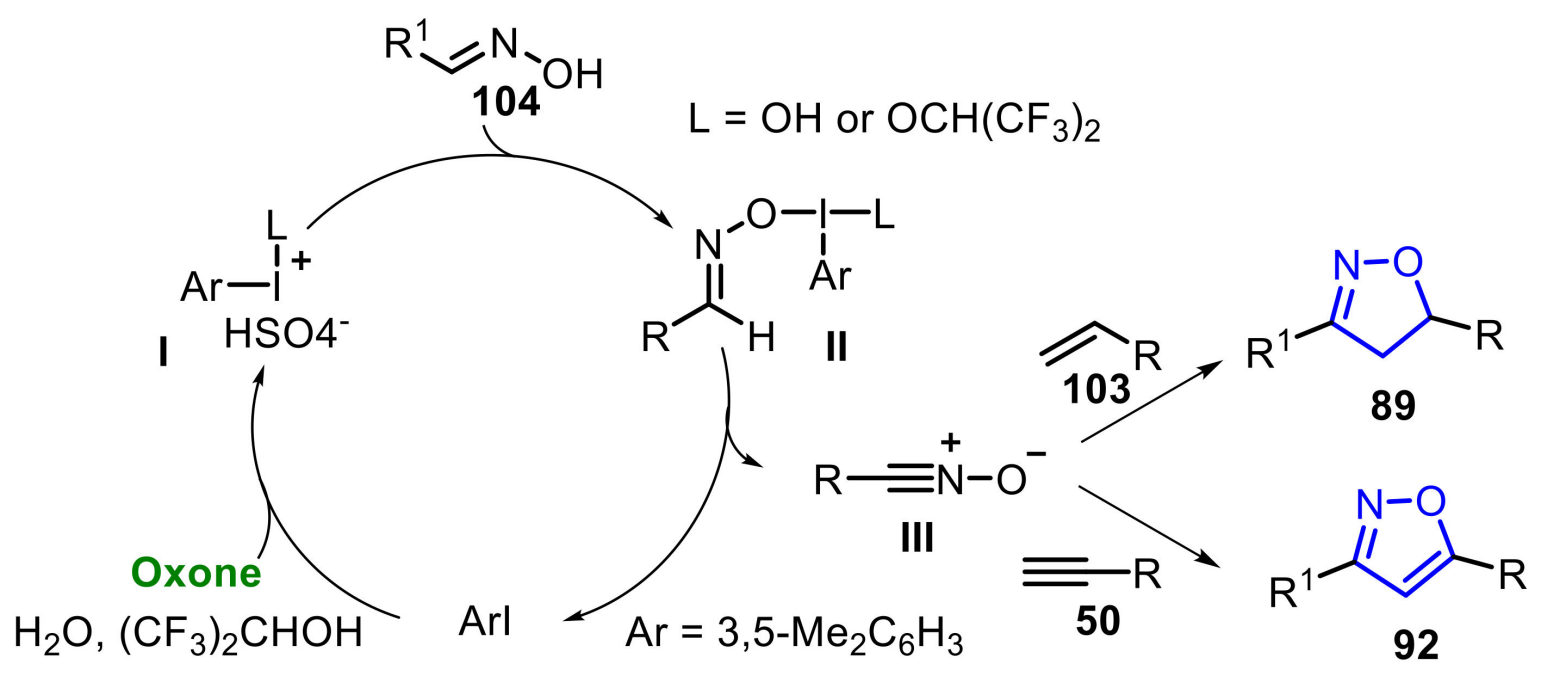
Plausible mechanism for the catalytic cyclization of the substrate **104**.

Based on control experiments, a plausible catalytic cyclization mechanism was proposed (Scheme 73). Initially, the activation of iodoarene by Oxone in aqueous HFIP affords the [ArI(OH)]^+^ species **I**, which reacts with aldoxime **104** to form the hypervalent alkoxy iodane **II**, via ligand exchange. Then, the intermediate **II** undergoes reductive elimination of iodoarene to produce the nitrile oxide **III**, which finally reacts with alkene **103** or alkyne **50** to produce the corresponding isoxazoline **89** or isoxazole **92**. The regenerated iodoarene continues the next catalytic cycle.

## 6. Conclusions

This review highlights the recent advances in the Oxone-mediated synthesis of heterocyclic compounds, bringing mechanistic insights, protocol particularities, and a comprehensive discussion about scope and limitations. Among the synthetic approaches discussed herein, several examples are conducted under mild reaction conditions, an environmentally friendly reaction medium and by using alternative energy sources. However, despite the impressive advances delivered so far, many improvements are still required. The finds reached until now are the base to develop innovative approaches in coming years, employing Oxone as a green oxidant to promote the formation of a diversity of chemical bonds, to construct important structure cores. Additionally, we also see good prospects in the application of ultrasound irradiation as a low-demand energy source, driving the application of Oxone in synthetic chemistry to new horizons by triggering innovative activation pathways. In this context, we hope that this review can be a solid source of information, inspiring those interested to devote their studies to the application of Oxone in organic synthesis, aiming to deliver more efficient, robust and greener synthetic approaches.
